# Comparative genomic characterization of citrus-associated *Xylella fastidiosa *strains

**DOI:** 10.1186/1471-2164-8-474

**Published:** 2007-12-21

**Authors:** Vivian S da Silva, Cláudio S Shida, Fabiana B Rodrigues, Diógenes CD Ribeiro, Alessandra A de Souza, Helvécio D Coletta-Filho, Marcos A Machado, Luiz R Nunes, Regina Costa de Oliveira

**Affiliations:** 1 Núcleo Integrado de Biotecnologia – Universidade de Mogi das Cruzes, Av. Dr. Cândido Xavier de Almeida Souza 200, Mogi das Cruzes, SP 08780-911, Brazil; 2 Centro Interdisciplinar de Investigação Bioquímica – Universidade de Mogi das Cruzes, Av. Dr. Cândido Xavier de Almeida Souza 200, Mogi das Cruzes, SP 08780-911, Brazil; 3 Centro APTA Citros Sylvio Moreira – Instituto Agronômico de Campinas, Caixa Postal 04, Cordeirópolis, SP 13490-970, Brazil

## Abstract

**Background:**

The xylem-inhabiting bacterium *Xylella fastidiosa *(*Xf*) is the causal agent of Pierce's disease (PD) in vineyards and citrus variegated chlorosis (CVC) in orange trees. Both of these economically-devastating diseases are caused by distinct strains of this complex group of microorganisms, which has motivated researchers to conduct extensive genomic sequencing projects with *Xf *strains. This sequence information, along with other molecular tools, have been used to estimate the evolutionary history of the group and provide clues to understand the capacity of *Xf *to infect different hosts, causing a variety of symptoms. Nonetheless, although significant amounts of information have been generated from *Xf *strains, a large proportion of these efforts has concentrated on the study of North American strains, limiting our understanding about the genomic composition of South American strains – which is particularly important for CVC-associated strains.

**Results:**

This paper describes the first genome-wide comparison among South American *Xf *strains, involving 6 distinct citrus-associated bacteria. Comparative analyses performed through a microarray-based approach allowed identification and characterization of large mobile genetic elements that seem to be exclusive to South American strains. Moreover, a large-scale sequencing effort, based on Suppressive Subtraction Hybridization (SSH), identified 290 new ORFs, distributed in 135 Groups of Orthologous Elements, throughout the genomes of these bacteria.

**Conclusion:**

Results from microarray-based comparisons provide further evidence concerning activity of horizontally transferred elements, reinforcing their importance as major mediators in the evolution of *Xf*. Moreover, the microarray-based genomic profiles showed similarity between *Xf *strains 9a5c and Fb7, which is unexpected, given the geographical and chronological differences associated with the isolation of these microorganisms. The newly identified ORFs, obtained by SSH, represent an approximately 10% increase in our current knowledge of the South American *Xf *gene pool and include new putative virulence factors, as well as novel potential markers for strain identification. Surprisingly, this list of novel elements include sequences previously believed to be unique to North American strains, pointing to the necessity of revising the list of specific markers that may be used for identification of distinct *Xf *strains.

## Background

The xylem-inhabiting bacterium *Xylella fastidiosa *(*Xf*) [1,2] has emerged, during the past decades, as an important phytopathogen, specially due to its implication with the development of Pierce's disease (PD) in North American vineyards and citrus variegated chlorosis (CVC), which affects orange trees in South America. PD was first detected in Southern California in 1884, when it destroyed approximately 40,000 acres of grapes in Anaheim, CA, during a 5-year outbreak of the disease (reviewed in [3]). After this devastating experience, PD remained as a minor concern to the West Coast viticulture for decades until the mid-1990s, when a new insect species, the glassy-winged sharpshooter (GWSS) *Homalodisca vitripennis *was accidentally introduced into Southern California and begun spreading northward. This leafhopper, which can serve as a vector to *Xf*, has the capacity to feed in more than 70 different plant species and survive winter temperatures dropping as low as 20°F [4]. Moreover, unlike other *Xf*-carrying insects associated with PD in California, GWSS has a much broader flight range (up to a quarter mile), posing a very serious threat to the wine industry from Southern and Central California [5]. Indeed, since the first identification of GWSS in the California vineyards, programs aimed at controlling the dissemination of this insect as a strategy to prevent PD outbreaks have involved more than US$ 160 million of direct investments [6].

Citrus variegated chlorosis, on the other hand, was originally identified in Brazil in 1987, during an outbreak that affected orange orchards distributed along the Northern and Northwestern regions of State of São Paulo [7], one of the most important areas of citrus production in this country, which turns out to be one of the world's leading producers of concentrated orange juice (reviewed in [8]). Since its initial observation, the disease incidence increased by graft propagation of *Xf*-infected budwood and by the action of many different sharpshooter vectors, becoming widely distributed across all citrus-growing regions in the country, where it is held responsible for damages that may reach US$ 280 – 320 million per year [9] (see also [10] for recent statistics about CVC in Brazil).

Different *Xf *strains have also been obtained from alternative host plants across the Americas and, in many cases, there seems to be a direct correlation between *Xf *infection and the development of diseases [11]. Thus, *Xf *strains are also believed to be responsible for phony peach disease (PP), alfalfa dwarf disease, periwinkle wilt and leaf scorch diseases in plum, elm, maple, oak, sycamore and coffee [12,13]. Up to now, none of these diseases has demonstrated to be as economically damaging as either PD or CVC. Nonetheless, *Xf *is already considered a major agronomical concern in the American continent, given the economic losses already experienced by both citrus and winegrape industries, as well as the widespread distribution of *Xf *strains in so many economically important crops. Thus, this microorganism has been the subject of increasing attention by many research programs after the mid 1990's [14] and, as a consequence of these efforts, one *Xf *strain (9a5c), associated with CVC in Brazil, turned out to be the first plant bacterium to have its complete genome sequenced and annotated [15]. Moreover, the existence of different disease symptoms, observed in a wide range of plant hosts [16] and associated with genetically distinct *Xf *strains, has led researchers to hypothesize that total genome comparisons among these bacteria could help to uncover information regarding genes involved in the interaction with specific hosts and disease development [17]. Thus, sequencing efforts have been extended to other *Xf *strains and subsequently, the genomes of two other strains (Ann-1 and Dixon), obtained from oleander and almond trees had their genomes partially sequenced and annotated [18]. Finally, a fourth strain, Temecula-1, isolated from grapevines and responsible for PD in California has also been sequenced to completion [19]. Thus, *Xf *is one of the best models available to conduct functional and comparative genomic studies.

Genomic sequences from these *Xf *strains have been submitted to extensive *in silico *evaluations, allowing the formulation of virtual metabolomes that provided a comprehensive view of the major biochemical processes that occur in these microorganisms [15]. Additional information regarding the functionality of different gene products and pathogenicity mechanisms in *Xf *have also been obtained by the evaluation of differential gene expression through microarray hybridization approaches and by the generation of gene-knockout mutants [20,21], while genomic comparisons conducted with these four strains allowed the identification and categorization of genome-wide DNA variations, as well as their influence on strain functional divergence [22]. Multiple alignment of chromosomal sequences identified SNPs and INDELs that could be used to estimate the relative similarity between the strains and the rates of genome evolution, which seem to be different for each individual strain [22]. All unique genes have been catalogued and, since their sequences could represent strain-specific markers, primer pairs were designed against these ORFs to assist in PCR-based detection of these four *Xf *strains in the wild [22,23].

The genomic information also established a solid base for the development of epidemiological and phylogenetic studies within the *Xf *group, providing evidence that the bacterial species *X. fastidiosa*, originally characterized from 25 strains (obtained from 10 different hosts), constitute a significantly complex group of plant-associated bacteria [14]. In spite of sharing a considerable amount of both phenotypic and genotypic similarities, as well as an overall 85% DNA sequence identity, as measured from DNA homology studies [2], *X. fastidiosa *strains and strains have been shown to display significant biological variability, which was confirmed by phylogenetic analyses involving sequence comparison of seven chromosomal genes (spanning almost 10 Kb of DNA sequence), sequencing of the ribosomal intergenic spacer (ITS), serological classification and microarray analyses of their genomic profiles [14,24-27].

However, all these studies involved a relatively small number of South American *Xf *strains, hampering a more conclusive evaluation about the biogeographical distribution, phylogenetic history, evolution and taxonomic relationships among this group of strains, which have not been as thoroughly studied as the North American strains. A similar situation is verified when *Xf *strains are analyzed and compared at the genomic level, since three North American strains have been submitted to genomic sequencing, as opposed to only one South American strain. Thus, it is possible that the relative lack of sequence information from other South American strains may have introduced biases to some of the conclusions drawn from recent genomic studies within the group. This situation prompted us to conduct a comprehensive genomic survey involving a total of 6 South American *Xf *strains, all obtained from infected orange trees [28]. We employed a microarray-based approach to compare the genomic profiles of these bacteria with *Xf *strain 9a5c and Suppressive Subtraction Hybridization (SSH) to identify new genes present exclusively in the genomes of these microorganisms. The results obtained from such analyses represent the first genome-wide evaluation regarding genome structure and composition from CVC-associated bacteria, providing additional information about the characteristics of the South American *Xf *gene pool and its relationship with what has been found in North American strains.

## Results

### Genomic comparisons among *Xf*-CVC strains through microarray hybridization analysis

**Figure 1:**
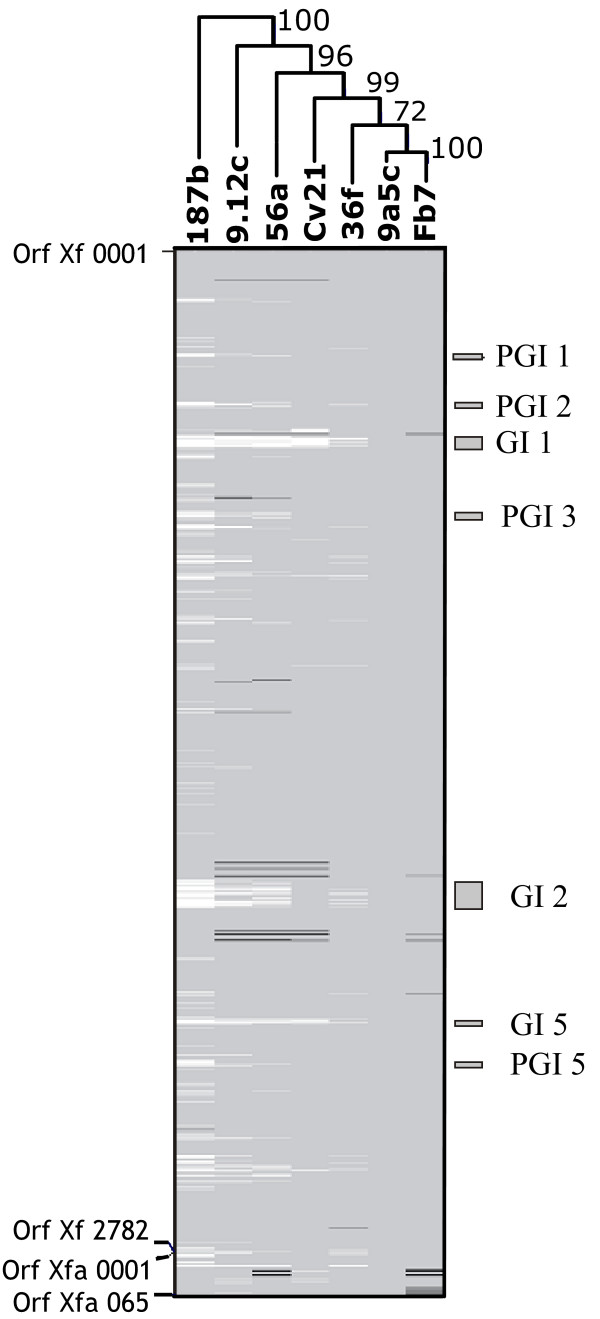
Genomic comparison of citrus-associated *Xylella fastidiosa *(*Xf*) strains by microarray hybridization showing the distribution of ORFs with reference to the genome of *Xf *strain 9a5c. Chromosomal ORFs are linearly represented, from ORF *Xf*0001 to ORF *Xf*2782, followed by ORFs from plasmid p*Xf *5.1 (from ORF *Xf*a0001 to *Xf*a0065). ORFs present in the genome of each tested strain are represented in grey, while missing ORFs are represented in black. ORFs present in greater copy number in the genomes of the tested strains are represented in white [see 26]. The genomic profiles were used to construct a hierarchical grouping of the strains and the robustness of the branching profile was verified by bootstrap analysis, using TMEV (the number next to each branch represents the bootstrap values for 100 permutations). The location of Genomic Islands (GIs) and putative Genomic Islands (pGIs) in the genome of *Xf *strain 9a5c are also shown [see 26].

Microarray hybridization has been widely used to undertake genomic comparisons involving a great number of microorganisms [29] and previous work from our group has established precise criteria to employ this methodology to the study of *Xf *strains, with the aid of an *Xf *9a5c biochip [30]. Thus, we performed similar microarray-based comparisons with 6 different *Xf *strains obtained from citrus plants (Table 1). Four of these *Xf *strains (56a, 9.12c, 187b, and 36f) were obtained from CVC-affected trees and are representatives of the most prevalent *Xf *haplotypes found in sweet orange orchards across the state of São Paulo, while *Xf *strain Cv21 was obtained from a non-symptomatic tree from the same region [28]. *Xf *strain Fb7, on the other hand, was obtained from a sweet orange tree that displayed symptoms of "Pecosita", a disease similar to CVC that occurs in some citrus-growing regions of Argentina [31]. The genomic profiles obtained for these 6 strains are depicted in Figure 1, which displays a linear representation of the *Xf *strain 9a5c chromosome (from ORF *Xf*0001 to *Xf*2782), followed by ORFs present in p*Xf*51, the large 51 kb chromosome present in *Xf *strain 9a5c (from ORF *Xf*a0001 to *Xf*a0064). Surprisingly, these results showed that, contrary to what had been observed during our former comparisons [26], most genomic differences within the citrus strains are not associated with deleted ORFs, but with elements that are present in greater copy number in the tested strains, when compared to *Xf *strain 9a5c (see Table 2, and Additional File 1). Nonetheless, we were able to confirm the same distribution pattern observed before, since most duplicated ORFs are not scattered throughout the genome, but grouped within mobile genetic elements, such as prophages, Genomic Islands (GIs) and putative Genomic Islands (pGIs) previously observed in the genome of *Xf *strain 9a5c [26].

**Table 1:** *Xylella fastidiosa *(Xf) strains used in this study.

*Xf *Strain	Host	Geographical Origin
9.12c	*Citrus sinensis *cv. Pera	Gavião Peixoto, SP – Brazil
56a	*Citrus sinensis *cv. Sanguinelli	Ubarana, SP – Brazil
187b	*Citrus sinensis *cv. Valência	Ubarana, SP – Brazil
36f	*Citrus sinensis *cv. Matidije Navel	Ubarana, SP – Brazil
Cv21	*Citrus sinensis*	Colina, SP – Brazil
Fb7	*Citrus sinensis *cv. Valencia	Bella Vista, Corrientes – Argentina

**Table 2:** Number of ORFs found to be deleted or present in higher copy number in the strains analyzed in this study, using as a reference the genome of *Xylella fastidiosa *strain 9a5c. A complete list of such ORFs is available online as Additional File 1

	187b	36f	56a	9.12c	Cv21	Fb7
Number of ORFs present in higher copy number in the genomes of the strains used in this study	337	46	92	129	49	00
Number of ORFs found to be deleted in the genomes of the strains used in this study	00	01	26	19	15	27

Details regarding differences in ORF composition, detected by the microarray hybridizations with all *Xf *strains analyzed in this study, can be found in Additional File 1. As expected, each strain presents a unique genomic profile, which can be used to characterize all individual strains with high fidelity, as inferred from the high bootstrap values obtained from the cluster analysis shown in Figure 1. Interestingly, the Argentine strain, Fb7, seems to be extremely similar to *Xf *strain 9a5c, as the genomic differences detected between these two strains are restricted to a small number of deleted ORFs, distributed along the main chromosome and plasmid p*Xf*51. All other strains display significantly different profiles, characterized not only by deletions, but also by duplicated ORFs. A strong similarity is observed in the profiles obtained for *Xf *strains 56a and 9.12c, characterized by scattered duplication of ORFs from GI2, and by what seems to be complete duplications of GI1 and GI5. Several ORFs from prophage *Xf*P4 and GI4 are also missing in these two strains. *Xf *strain Cv21 presents a genomic profile that closely resembles *Xf *strains 56a and 9.12c, except for the lack of duplications involving ORFs within GI2. *Xf *strain 36f, on the other hand, displays another type of profile, in which no deletions have been detected along the structures of *Xf*P4 and GI4 and the duplicated ORFs scattered across GI1 and GI5 seem to indicate that duplication of these elements is not complete. Finally, *Xf *strain 187b displays the most divergent genomic profile, characterized by a very large number of duplicated ORFs and mobile elements. Once again, duplications seem to span practically the entire structure of GI1, GI5 and even GI2. Moreover, several other duplicated regions seem to occur throughout the genome of *Xf *strain 187b, pointing to the possible existence of other mobile genetic elements in *Xf*. Interestingly, at least three of these regions seem to map within elements that had been previously identified as putative Genomic Islands (pGIs) [26].

The two largest *Xf *GIs (GI1 and GI2), which were found to be deleted in North American strains are present in all citrus strains analyzed herein and GI1 seems to be completely duplicated in at least four of the tested *Xf *strains (187b, 56a, 9.12c and Cv21), as well as in two previously analyzed citrus *Xf *strains, X1-B14 and SJ [26], indicating that this element seems to display intense transpositional activity among representatives of the *Xf *group. Interestingly, GI1 seems to be specific to strains obtained from citrus and coffee trees from South America and may represent a sinapomorphy for the South American *Xf *strains [26]. GI5, which is also duplicated in other *Xf *strains (56a and 9.12c), displays a region that is similar to the VapE-containing region of GI1, resembling the situation observed with the Vap elements from the pathogenic bacterium *Dichelobacter nodosus *(see below [32]).

### Identification of new ORFs in the Xf-CVC gene pool of citrus-associated *Xf *strains through Suppressive Subtraction Hybridization (SSH)

Since the *Xf *biochip used in the experiments described above was based on the genome of *Xf *strain 9a5c, the hybridization experiments can only provide information regarding genes that are present in this strain. Thus, to gather information concerning additional ORFs present in the gene pool of citrus-associated strains, we employed Suppressive Subtraction Hybridization (SSH), using DNA from *Xf *strain 9a5c as a driver, against DNA from all 6 strains, as described in Methods. Thus, a total of 18 SSH libraries have been constructed (using three different restriction enzymes for each strain) and approximately 9,000 clones have been obtained and sequenced from these libraries (Table 3).

**Table 3:** Number of SSH clones obtained and sequenced for each *Xylella fastidiosa *(*Xf*) strain

*Xf *Strain	Restriction enzyme used in the SSH reactions		
	
	*Rsa *I	*Alu *I	*Dra *I
187b	1.344	384	384
Cv21	688	160	288
56a	480	140	124
9.12c	958	704	424
36f	960	720	192
Fb7	864	144	288

**Figure 2:**
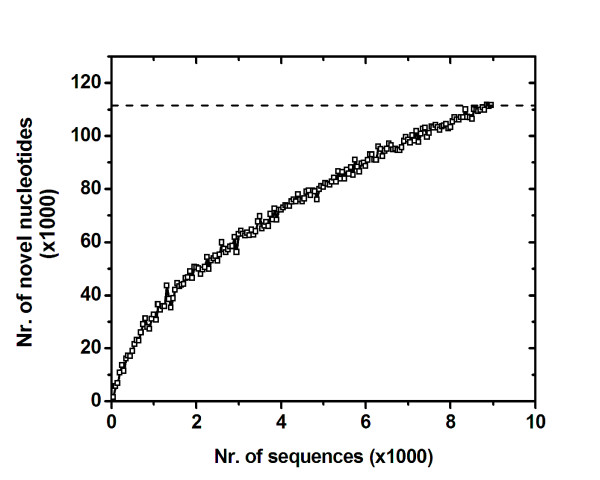
Redundancy analysis of the SSH experiments. To verify the effectiveness of the SSH sequencing approach to continually identify novel sequences in the genomes of the *Xf *strains in this study, increasing numbers of SSH sequences were submitted to clustering analyses with Phred/CAP3 and the consensus sequences obtained through this analysis were filtered against the genomic sequence of *Xf *strain 9a5c. The number of novel nucleotides identified through this approach was plotted as a function of sequenced SSH clones. The inclination of the curve indicates that the SSH approach is still capable of identifying novel sequences in the genomes of the tested strains, although it is likely that most such sequences have already been obtained.

For each of the 6 strains, the sequenced clones have been trimmed, in order to exclude vector sequences and poor quality regions (Phred < 20), and aligned with the aid of CAP3, generating 1,063 contigs, which contained 6,712 sequences overall. These contigs span 217.1 Kb of *Xf *sequences, which is equivalent to approximately 7.7% of the genome of *Xf *strain 9a5c (Table 4). We chose to conduct further analyses only with contigs composed by at least 6 different individual sequences and showed size variation from 400 to 4,500 bp. Thus, a total of 2,534 sequenced clones were excluded from further studies, since they either remained as singlets or grouped into small contigs, composed by a limited number of reads (2 in most cases), resulting in poor quality consensus sequences. Next, the contigs were filtered against the genome of *Xf *strain 9a5c with the aid of the software cross_match [33], allowing the identification of stretches of DNA that were exclusive to the tested strains. Sequencing of approximately 9,000 SSH clones has allowed the identification of 111.53 Kb of DNA sequences that are not present in the genome of *Xf *strain 9a5c (which represents ~4.1 % of total genome size). The rate at which novel sequences were identified, as a function of sequenced SSH clones, has been evaluated as described in Methods, and judging by the inclination of the curve shown in Figure 2, a significant proportion of the novel sequences present in the analyzed genomes is likely to have been identified in this study. These newly identified sequences were analyzed with GeneMark [34], to search for individual elements present in their structure, allowing the identification of 290 new ORFs, scattered throughout the genomes of the 6 strains analyzed herein (see GenBank accession numbers ER935541 to ER935830). This represents an increment of approximately 10.2% in the number of ORFs currently known to belong to the gene pool of citrus-associated *Xf *[15].

**Table 4:** Number and size of contigs obtained for each *Xylella fastidiosa *(*Xf*) strain

*Xf *Strain	Cv21	187b	56a	Fb7	9.12c	36f	Total
Number of contigs assembled by CAP3	97	393	58	125	208	182	1063
Overall number of base pairs in the assembled contigs (in Kb)	37.7	40.7	29.9	31	52.8	25	217.1

**Figure 3:**
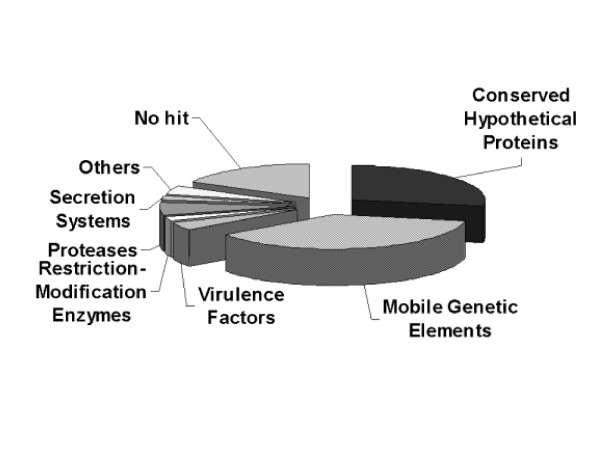
Functional distribution of the 135 Groups of Orthologous Elements (GOEs) that have been identified in the genomes of these strains through SSH analysis. These sequences were obtained after alignment of sequenced SSH clones from each strain. The consensus sequences for each contig were filtered against the genome of *Xf *strain 9a5c and the remaining sequences were analyzed with GeneMark to identify new ORFs. The sequences from such ORFs have been clustered and the resulting contig consensuses (or singlet sequences) have been submitted to Blastx analyses against the GenBank. Functional classification was done according to Simpson and coworkers [15].

However, comparative analyses among these 290 newly identified ORFs has shown that a large proportion of these elements (228 ORFs) seemed to belong to different groups of orthologous proteins, present in the genomes of two or more tested strains. Thus, to reduce the redundancy of this dataset, the predicted protein sequences from these 290 ORFs have been submitted to cluster analysis, as described in Methods, resulting in 135 Groups of Orthologous Elements (GOEs), which are more likely to represent the actual number of new functional genes identified in the gene pool of the tested *Xf *strains (see Additional File 2). Analyses of similarity against the GenBank have then been performed with Blastx, using the consensus sequences from each GOE as input, which allowed the assignment of putative functions for each of these newly identified elements. Their distribution into the different functional categories originally described by Simpson and co-workers is shown in Figure 3[15]. Surprisingly, a relatively small proportion of such sequences has been identified as "no hit" (only 23, which is equivalent to ~17% of the newly identified GOEs). Another large fraction of elements (37) has been identified as conserved hypothetical proteins (~28% of the total), displaying high similarity to ORFs of unknown function, previously identified in the genomes of other microorganisms – particularly in other *Xf *strains (see below). Putative functions could be attributed to 75 newly identified GOEs (~55%) and the majority of them (50) are directly associated with mobile genetic elements, since we identified 2 new phage structural proteins, 8 recombinases/integrases, 14 elements involved with plasmid replication/stabilization and 26 elements that are homologous to conjugation factors belonging to the TraA/TraB/Cag/Vir families, originally described in *Agrobacterium tumafasciens *[35]. The remaining 25 GOEs, (18.5%) encode proteins that are involved with several metabolic processes of the cell, including a group of six new potential virulence factors, which had not been originally identified in the genome of *Xf *strain 9a5c. These include two homologues for the transcription factor AbrB (GOEs #17 and #43 in Additional File 1), a new pilin gene (GOE #76 in Additional File 1), two Lpx acetyltransferases (GOE #26 and #93 in Additional File 1) and a gene encoding the *Zonula Ocludens Toxin *(Zot) from *Vibrio cholerae *(GOE #51 in Additional File 1).

**Figure 4:**
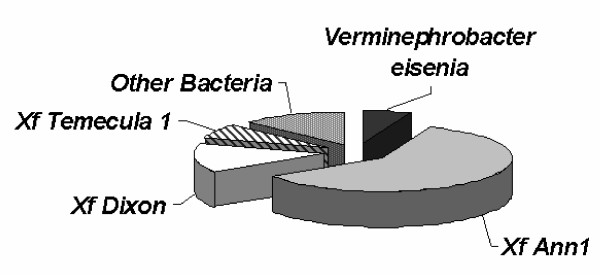
Identification of the microorganisms carrying the closest homologue for each of the 135 Groups of Orthologous Elements (GOEs) that have been identified in the genomes of the tested strains through SSH analysis. These sequences were obtained after alignment of sequenced SSH clones from each strain. The consensus sequences for each contig have been filtered against the genome of *Xf *strain 9a5c and the remaining sequences were analyzed with Genemark to identify new ORFs. The sequences from such ORFs have been clustered and the resulting contig consensuses (or singlet sequences) have been submitted to Blastx analyses against the GenBank. The most significant Blast hit was considered for this analysis.

The Blastx analyses also confirmed that the sequences from all 135 elements described above could not be found in the genome of *Xf *strain 9a5c. Surprisingly, however, these analyses showed that a large proportion of these sequences (67.5%) has already been identified in *Xf *strains from North America – particularly in the case of *Xf *Ann-1, isolated from oleander (Figure 4). As shown in Additional File 3, the consensus sequences from 67 GOEs, showed high similarity to ORFs originally described in *Xf *strain Ann-1, encoding both hypothetical proteins and proteins with assigned functions. Sixteen GOEs showed high similarity with elements identified in the genome of *Xf *strain Dixon (isolated from almond trees) and eight were most similar to sequences found in *Xf *strain Temecula-1 (isolated from grapevines). Such an overlap with genes from the North American *Xf *gene pool was unexpected and suggests that the list of unique ORFs, recently proposed by Doddapaneni and coworkers [22], as potential targets for strain-specific detection of North American *Xf *strains in the wild must be revised [36]. As shown in Table 5, approximately 27.7% of the ORFs, previously believed to be unique to *Xf *strain Ann-1 (23 out of 83) and 5.5% of the ORFs, previously believed to be unique to *Xf *strain Dixon (3 out of 54) have also been found in the citrus-associated strains analyzed in this study. On the other hand, since the consensus sequences from 23 GOEs returned "no hit" when compared to the NCBI databases, these elements might prove useful as targets for PCR-based detection of citrus-associated *Xf *strains (Figure 3).

**Table 5:** List of ORFs originally identified as specific for the North American *Xylella fastidiosa *(*Xf*) strains Ann-1 and Dixon, whose sequences have been found in the genomes of the *Xf *strains used in this study. The presence of sequences related to each ORF in the genomes of the strains analyzed by us is marked by an X.

ORFa	Identified in this study as	Putative function	187b	56a	9.12c	Cv21	Fb7	36f
Ann FY0184	GOE #17	Transcriptional regulator AbrB	X	X			X	
Ann FY0185	GOE #127	Conserved hypothetical protein				X		
Ann FY0934	GOE #19	Putative transposase TnA	X	X				
Ann FY0977	GOE #133	Conjugation TrbI-like protein				X		
Ann FY0978	GOE #23	Conserved hypothetical protein	X		X			
Ann FY0979	GOE #16	Resolvase, N-terminal:Resolvase helix-turn-helix region		X		X		
Ann FY0983	GOE #20	Helix-turn-helix motif	X	X	X	X		
Ann FY0984	GOE #42	Conjugal transfer protein TrbG/VirB9/CagX		X	X	X		
Ann FY0985	GOE #52	Conserved hypothetical protein	X	X		X		
Ann FY0988	GOE #30	VirB8	X	X		X		
Ann FY0989	GOE #02	TrbL/VirB6 plasmid conjugal transfer protein	X	X	X	X		X
Ann FY0992	GOE #63	Plasmid-related exported protein				X		X
Ann FY0993	GOE #26	Transferase hexapeptide repeat	X		X	X		
Ann FY0996	GOE #07	Probable conjugal transfer protein TraL	X	X	X			X
Ann FY0997	GOE #59	Conserved hypothetical protein		X	X	X		X
Ann FY1071	GOE #56	Conserved hypothetical protein	X	X	X	X	X	X
Ann FY2430	GOE #68	Conserved hypothetical protein			X	X		
Ann FY2499	GOE #62	Conserved hypothetical protein			X			X
Ann FY2513	GOE #116	Helix-turn-helix motif:Peptidase S24, S26A and S26B	X					
Ann FY2526	GOE #31	type IV secretory pathway, VirB3 family protein		X	X	X		
Ann FY2545	GOE #12	Conserved hypothetical protein			X	X		
Ann FY2555	GOE #70	Conserved hypothetical protein				X		X
Ann FY3510	GOE #43	SpoVT/AbrB-like		X	X			
Dixon FX0341	GOE #71	Helix-turn-helix motif: Peptidase S24, S26A and S26B			X	X		
Dixon FX2654	GOE #107	Putative plasmid conjugal transfer protein TraJ					X	
Dixon FX3105	GOE #96	Conserved hypothetical protein					X	

^a ^As described by Doddapaneni and coworkers [22].

Altogether, the consensus sequences from only 21 GOEs showed similarity to genes identified in microorganisms that do not belong to the *Xf *group (Figure 4). Interestingly, one third of these elements (7) seem to be homologous to ORFs identified in the genome of *Verminephrobacter eiseniae*, a soil-inhabiting bacterium that infects nematodes [37], while the remaining ones show high similarity to genes found in a great number of microorganisms, such as *E. coli*, *Geobacter metalireducens*, *Salmonella enterica*, *Solibacter usitatus*, *Xanthomonas sp*., *Burkholderia *sp., *Chlorobium tepidum*, among others.

## Discussion

Results obtained from several lines of research, developed during the past decade, have turned the phytobacterium *Xylella fastidiosa *into a unique model of study in the fields of both phytopathology and genomics. First of all, this is due to the fact that several diseases, associated with many distinct bacterial strains have been characterized and some of these diseases are responsible for significant economic losses. Moreover, a significant amount of genomic information has been obtained for four different strains of this microorganism, allowing the development of both functional and comparative genomic analyses within the group. Comparisons performed with the four sequenced *Xf *genomes led Doddapaneni and coworkers to suggest that the Tenecula-1 strain genome is the one that most likely resembles the ancestral *Xf *genome, since it has undergone the fewest genetic changes among the four analyzed strains [22]. Thus, *Xf *strain 9a5c (as well as other South American strains) may have evolved from an ancestral bacterium, carrying a Temecula-1-like genome, through incorporation of a series of horizontally transferred elements, such as prophages, GIs and plasmids [26]. This possibility is reinforced by the microarray-based comparisons undertaken in this study with the citrus-associated strains, since they provide further evidence for intense transpositional activity of mobile elements during the evolution of this subgroup of *Xf*.

So far, most comparative studies among South American *Xf *strains have essentially involved Brazilian strains from both citrus and coffee trees. In general, the available data supports the idea that CVC-associated bacteria found in Brazil have evolved directly from *Xf *strains that cause coffee leaf scorch (CLS), since CVC-associated *Xf *strains have been shown to induce CLS symptoms when experimentally inoculated into coffee trees [38]. Moreover, it is widely known that most areas in which citrus orchards are presently cultivated used to be dedicated to coffee plantations and there have been reports of CVC vectors feeding on coffee trees [39]. Nonetheless, the exact origin of CVC-related *Xf *strains is still a matter of speculation, since very little research has been performed on Pecosita-related *Xf *strains. The origin of this subgroup of *Xf *is an important piece of information regarding the evolutionary history of citrus-associated *Xf *strains, specially considering that Pecosita has been known to occur in Argentina before the first descriptions of CVC in Brazil (reviewed in [31]).

Thus, the present study shows the first genomic-scale comparative evaluation involving a Pecosita-related *Xf *strain (Fb7). It is interesting to verify from these data that, while *Xf *strain Fb7 (isolated in 2000) appears to be very similar to *Xf *strain 9a5c (isolated back in 1987 [7]), significant genomic differences have been observed when these strains are compared to Xf-CVC bacteria isolated in more recent years, specially regarding the activity of horizontally transferred elements. Although it is tempting to speculate from this data that the evolutionary rates among CVC- and Pecosita-related strains might differ, it is clear that further phylogenetic and biogeographical studies have yet to be performed in order to shed more light into our knowledge regarding the evolutionary history of the South American *Xf *strains, as well as their corresponding diseases. An attempt to verify the evolutionary relationships between *Xf *strains 9a5c, Fb7 and the other *Xf *strains has been performed by sequencing the 16S-23S rDNA spacer region of these bacteria, but since no strain-specific mutations have been found in this sequence, such analysis turned out to be inconclusive (data not shown).

As mentioned before, the fact that the overall genomic information obtained for *X. fastidiosa *has been mostly based on sequence information derived from North American strains, was likely to have biased to some of the conclusions obtained so far, specially considering the evolutionary complexity of these bacteria, which have been shown to carry an extremely large and active flexible gene pool [26]. As expected, the SSH experiments described in this work, which constitute the first attempt to undertake a large-scale survey of the genomic composition of South American *Xf *strains, showed that a total of 26 ORFs, originally identified as unique to the North American strains also seem to be present in the citrus strains from South America [22]. Among these ORFs, 23 had been proposed to be exclusive to *Xf *strain Ann-1 and 3 were supposed to be present only in the genome of *Xf *strain Dixon. Thus, sequencing of SSH clones from citrus-associated *Xf *strains resulted in a reduction of approximately 19% in the list of unique genes that could be used as markers for North American strains (this number increases to ~27.7% if we consider only the list of genes believed to be unique to *Xf *strain Ann-1) [22,36]. These findings reinforce the importance of gathering more information regarding the genomic composition of South American strains – including strains isolated from other hosts, such as coffee, to help in understanding the evolutionary history of the *Xf *group and assist in the development of tools for specific identification of pathovars [40] and other variants.

At this point, it is not clear whether the increasing overlap between elements found in South and North American strains are a result of ancestry, or represent lateral transfer events among strains [41]. Although the geographical separation of South and North American strains presents a clear barrier to lateral gene transfer events, the evolutionary history of the *Xf *genome has been shown to be extremely dynamic, highly influenced by the activity of transpositional elements from its large flexible gene pool [26,42]. In fact, direct evidence for the occurrence of genetic exchange between *Xf *strains from the two continents has already been reported after analysis of one *Xf *strain, obtained from plum trees in South America [43]. Regardless of its geographical origin, the genomic profile obtained for this strain clearly indicated that it descended from North American strains and was likely to have been accidentally introduced into the South American continent via infected plant material. However, this strain carried practically all genes from p*Xf *51, a large plasmid found exclusively in South American *Xf *strains, which was likely acquired by conjugation events with South American strains [26].

Thus, given the fact that *Xf *strains are known to infect more than 100 plant species – many of which are commercial crops that are constantly shipped to foreign countries [13] (see also [44]), it is not unlikely to assume that other contamination events might have taken place, allowing the introduction of North American strains into the South American territory, and vice versa. Further conjugation events, for instance, might have introduced genes that were specific to the North American strains into the gene pool of South American strains, which could have been facilitated by the presence of such a large set of transposable elements in the *Xf *genome and the fact that multiple strains have been shown to coexist in the same host – both in the case of infected plants, as well as insect vectors [45,46].

The dynamics of *Xf *genome evolution can also be deduced through evaluation of other mobile elements, particularly in the case of GI1, which seems to be absent from the North American strains, but present in one or more copy numbers in South American strains, as shown in Figure 1. Among all mobile elements identified in *Xf*, GI1 is the one that best fulfills the definition of a Genomic Island, since it displays a higher GC content, altered codon bias, insertion at the 3'end of a tRNA gene (tRNA N) and the presence, at one end, of ORFs that display high similarity to a heterodimeric integrase found in association with an insertion element from *Helicobacter pylori *(ORFs *Xf*0535 and *Xf*0536) [47,48]. More interestingly, GI1 may be significant for the evolution of the *Xf *group, since all studies undertaken so far, involving a total of 10 different South American strains, obtained from both citrus and coffee, indicate that this element may represent a genomic sinapomorphy for the South American *Xf *strains [26]. At this point, it is not possible to determine if elements present in GI1 play any role(s) in mediating adaptation of South American *Xf *strains to their specific hosts, nor if they participate in the process plant infection and colonization. Nonetheless, this element carries a series of ORFs whose products are potentially involved with host adaptation and pathogenicity, resembling the structure of Pathogenicity Islands (PAIs) observed in other bacteria [49]. For instance, GI1 carries a unique fimbrillin gene, represented by ORF *Xf*0487. Fimbrillins are components of bacterial type I fimbriae, which are directly implicated in the process of attachment to different types of substrates and biofilm formation [50]. Novel fimbriae, composed by distinct fimbrillin genes, are believed to be important virulence determinants, allowing colonization of specific hosts and differentiation of virulent clonal groups of pathogenic bacteria, as in the case of *E. coli *strains, in which new fimbrillin isomorphs are also associated with horizontally transferred elements [51]. Interestingly, *Xf *strains that display specificity to different host and vector species also display a variable number of fimbrilling homologues scattered throughout their genomes [15,18,19].

GI1 has also been shown to carry toxin-associated genes, such as ORF *Xf*0513, which encodes a hemolysin-like protein, which is implicated in cytotoxic reactions associated with many pathogenic bacteria [52], and ORF *Xf *0486, involved in the synthesis of the LPS fraction of Gram-negative bacteria that mediates adverse reactions in both human and animal hosts during infection by pathogenic bacteria [53]. In the case of phytobacteria, the O-antigen portion of LPS has been shown to display a strain-specific pattern, which is believed to play a major role in host recognition during the plant colonization process [54]. Finally, GI1 also carries ORF *Xf*0506, which encodes the virulence-associated factor *vap*E from *Dichelobacter nodosus*, the causative agent of ovine footrot disease. In this bacterium, the *vap *genes are present in a family of Pathogenicity Islands called the Vap elements [32]. Interestingly, up to 3 different Vap elements, varying in length and ORF composition, have been mapped throughout the genome of *D. nodosus *and although their exact function(s) are still unknown, their presence has been clearly shown to be associated with the virulence phenotype in this bacterium [32]. Curiously, as mentioned above, the *vapE*-containing region of GI1 is also duplicated in *Xf *strain 9a5c, as a part of GI5, resembling the organization of Vap elements in *D. nodosus*.

As mentioned above, additional virulence factors have now been identified in the genome of citrus-associated strains through the SSH experiments. One of these elements encodes a new form of pilin, associated with type IV fimbriae, whose twitching motility mechanism has been shown to be of capital importance to host colonization in *Xf *[55]. This finding reinforces the importance of fimbriae for the evolutionary divergence of *Xf *strains. Transcriptional regulators, such as the *abr*B activator have also been found through the SSH experiments. This transcription factor is responsible for controlling several genes, specifically activated at the end of bacterial exponential growth phase in *Bacillus subtilis*, and has been shown to be implicated with biofilm formation in this bacterium [56]. Interestingly, biofilm formation is believed to be an important virulence factor in *Xf *during the development of both CVC and PD, since growth in biofilm is likely to participate in the process of bacterial adherence to the xylem vessels, contribute to xylem occlusion and increase bacterial survival against the oxidative burst mediated by infected plant tissues [57,58]. Two different homologues of the *abr*B gene have been found in 4 out of the 6 analyzed *Xf *strains (56a, 9.12c, Fb7 and 187b), while the new pilin gene has been found only in *Xf *strain 9.12c. Two other potential virulence factors found in the *Xf*-CVC gene pool seem to belong to the CysE/LacA/LpxA/NodL family of acetyltransferases, which are characterized by multiple repeats of the sequence [LIV]-G-X(4) [59]. In both cases, the highest similarities found for these newly identified acetyltransferases are with genes *lpx*A and *lpx*D from the North American strains Ann-1 and Temecula-1 [18,19], which are involved in synthesis and modification of the O-antigen fraction of Gram-negative bacteria LPS. As mentioned above, the strain-specific structure of the O-antigen has been show to be an important mediator in plant colonization by symbiotic bacteria, especially in the case of nodule formation during *Rhizobium*-legume interactions [59]. Finally, the genomes of *Xf *strains 187b and 9.12c seem to carry a copy of the gene that encodes the *Zonula Occludens Toxin *(Zot), also found in *Xf *strain Ann-1. *zot*-like genes have been found in association with several bacterial pathogens, such as *Xanthomonas*, *Vibrio *and *Stenotrophomonas*, among others [60] and are likely to be laterally transferred among microorganisms through the action of filamentous phages [61]. This toxin has been originally described as an important virulence factor in *Vibrio cholerae*, and seems to be responsible for the development of severe cases of diarrhea caused by *V. cholerae *strains that do not carry the cholera toxin gene *ctx*A. The activity of Zot as an enterotoxin seems to be associated to its capacity to interfere with tight junctions of the gastrointestinal epithelium, altering its permeability to water and other substances [61,62]. Nonetheless, regardless of its recognized importance as a virulence factor in animal or human infections, there is no direct evidence that Zot plays any role(s) during plant colonization and/or pathogenicity, although this gene has also been found in the genome of *Xanthomonas campestris*, the causative agent of black rot disease, characterized by hyper-hydration of infected plant tissues, associated with wet edged lesions, which may be a result of Zot activity [63].

## Conclusion

The experiments described in the present study represent the first attempt to conduct microarray-based genomic comparisons and a large-scale survey of genes present in the genomes of South American *Xf *strains. The results from the microarray-based comparison provide further evidence concerning the intense transpositional activity of several horizontally transferred elements and reinforce previous studies regarding the importance of lateral gene transfer as a major mediator in the evolution of this important group of phytopathogens. Moreover, comparison of the microarray-based genomic profiles showed similarity between *Xf *strains 9a5c and Fb7, which is unexpected, given the geographical and chronological differences associated with the isolation of these microorganisms. It is clear that a more comprehensive evaluation of both coffee- and Pecosita-related strains is necessary before we can fully understand the evolutionary history of South American *Xf *strains and their associated diseases.

The characterization of approximately 9,000 SSH clones, from six representatives of citrus haplotypes, have now provided a more comprehensive view of the size and composition of the *Xf*-CVC gene pool, allowing us to identify 290 new ORFs – a number that represents an ~10.2% increase in our current knowledge of the South American *Xf *gene pool. These results allowed identification of new putative virulence factors, as well as novel potential markers for strain identification within this subgroup of phytopathogens by molecular-based approaches. Moreover, several sequences previously believed to be unique markers for North American strains have now been found in the genomes of these South American strains [22]. These new data point to the necessity of revising the molecular markers currently accepted as potential targets for identification of distinct *Xf *strains in the wild [22]. Finally, by evaluating the rate at which novel sequences have been identified through the SSH approach, we conclude that the complete composition of the South American *Xf *gene pool can still be stretched, specially if this type of analysis is further extended to strains obtained from alternative hosts, such as coffee, which is known to harbor many different *Xf *strains throughout the South American territory [64].

## Methods

### Strains, growth conditions and DNA extraction

All *Xf *strains used in this work (Table 1) have been isolated by our research group and are deposited at the culture collection of the Centro APTA Citros Sylvio Moreira. Those interested in obtaining samples of these strains and/or more specific information should contact HDCF helvecio@centrodecitricultura.br. Four *Xf *strains (9.12c, 56a, 187b, and 36f) have been previously described and are representatives of the most prevalent *Xf*-CVC haplotypes found out of 360 strains obtained from the northwestern, central, western, and southern regions of the State of São Paulo [28]. *Xf *strain Cv21 was obtained in February 2001 from a non-symptomatic sweet orange tree (*C. sinensis*) in the city of Colina, SP. The tree was grafted onto a *Poncirus trifoliate *rootstock and was present within a highly CVC-infected orchard. *Xf *strain Fb7 was isolated in October, 2000, from a ten-year-old sweet orange tree (*C. sinensis *cv. Valencia) grafted onto a *Poncirus trifoliate *rootstock in the province of Corrientes, Argentina. The tree had several branches carrying leaves that displayed typical Pecosita symptoms [65].

For strain isolation, 3–5-mm-diameter branches were collected, surface disinfected, cut in the middle and the internal ends were squeezed with a pair of pliers. The sap was blotted onto BCYE agar plates [66] and incubated at 28°C for 15 to 20 days. Isolated colonies were selected using a stereo-microscope, streaked onto fresh BCYE agar plates, and incubated at 28°C for 7 days. Identification of *X. fastidiosa *was carried out based on fastidious *in vitro *growth, white color of colonies, and PCR assays using primers specific to CVC-causing strains of *X. fastidiosa *[16]. All strains were have been maintained at -80°C. For this work, the bacteria were recovered on PW agar medium [67] and the plates maintained for 10 days at 28°C. The colonies were transferred once to new plates containing the same medium, grown for 20 days and harvested for DNA extraction using the protocol developed by Wilson [68].

### Microarray fabrication

*Xf *microarrays have been constructed as previously described [26,30]. Briefly, representative sequences from approximately 2200 ORFs from the *Xf *strain 9a5c genome (> 90% coverage) were PCR amplified, purified and spotted onto CMT-GAPS silane-coated slides (Corning), using an Affymetrix 427 arrayer, according to the manufacturer's instructions.

### DNA labeling and hybridization conditions

Labeling reactions and purification were performed as described in Nunes and coworkers [26]. Arrays were hybridized overnight (42°C) in a GeneTac Hybridization Station (Genomic Solutions, Inc – Ann Arbor, MI), in 6 × SSC, 5 × Denhardt's solution, 0.25 mg/ml sheared salmon sperm DNA, 0.5% SDS and 2 μg of each labeled DNA sample. After hybridization, slides were washed twice (42°C) in 0.5 × SSC, 0.01% SDS, followed by two washes in 0.06 × SSC, 0.01% SDS and two final washes in 0.06 × SSC. All washing steps consisted of 1 minute of flow, followed by 5 minutes of incubation. Slides were then dried and submitted to fluorescence detection.

### Image acquisition and analysis

Hybridized arrays were scanned in an Affymetrix 418 Array Scanner and images were analyzed with Affymetrix Jaguar v 2.0 [69]. Quality control of the hybridized spots was automatically performed by the software, based on spot morphology and local signal-to-background ratio, using the Easy Threshold and Variable Circle Size Algorithms [69]. In all experiments, reliable hybridization signals were obtained for more than 90% of the arrayed probes (see [30]). Normalization between the intensities in the two channels was achieved with the Jaguar Control Spots option, using a list of 30 control ORFs that shared sequence identity in the genomes of *Xf *strains 9a5c and Temecula-1. For each pair of strains, two independent hybridizations were performed. Since each microarray carried 2 copies of the arrayed genes, these hybridizations resulted in a total of 4 measurements for each probe in the microarray. These data were consolidated into a GATC database with Affymetrix MicroDB v.2.0 and the averages from all six readings were submitted to scatter plot visualization with Affymetrix Data Mining Tool v.2.0. Statistical validation of fold change variations was performed with the aid of the Significance Analysis of Microarrays (SAM) method proposed by Tusher and coworkers [70]. Spots that showed a Reference/Test ratio < 1:2 were considered to be present in greater copy number in the test over the reference strain, as proposed by Smoot and coworkers [71], while spots that showed an average Reference/Test ratio > 5:1 were considered to be missing in the test strain. The application of these criteria in a direct sequence comparison between *Xf *strains 9a5c and Temecula-1, which have been completely sequenced, provided an estimated error rate below 0.3% [30]. Raw and normalized data from all microarray hybridizations, as well as the microarray complete annotation file have been submitted (in MIAME-compliant format) to NCBI's Gene Expression Omnibus (GEO) and can be assessed through Series number GSE 8493.

Genome comparisons were viewed and compared using TIGR Multi-Experiment Viewer (TMEV), v.4.0 [72]. For the visualization of comparative profiles from each analyzed strain, we applied the method proposed by Smoot and coworkers [71], where ORFs shared by the reference and each test strain were labeled 0, while ORFs exclusive to the reference strain, or present in greater copy number in the test over the reference strain were labeled 1 and -1, respectively.

### Suppressive Subtraction Hybridization

Suppressive subtraction hybridization was performed essentially as described by Agron and coworkers [73]. Briefly, 4 μg of both tester and driver DNA were individually digested with the different restriction endonucleases chosen for this study, in a 200 μL reaction for approximately 16 hs. Next, the reactions were terminated by the addition of 1 μL 0.5 M EDTA, heated to 65°C for 30 minutes and the DNA was purified by phenol-chloroform and concentrated to 10 μL ddH_2_O, after ethanol precipitation. Two small samples of each digested tester DNA (120 ng) were ligated to their specific adaptors 1 and 2 (see [73]), in two separate ligation reactions. Each ligation was performed in a 10-μL final volume, containing 1 μL (200 units) of T4 DNA ligase and 1 μL of 10× ligation buffer (New England BioLabs), at 16°C for 16 hs. The first step of the subtraction hybridization was then performed, mixing 12 ng of each adaptor-ligated tester DNA to 600 ng of digested driver DNA, in a 5 μL reaction volume, containing 250 mM Hepes (pH8.3), 2.5 M NaCl, 1 mM EDTA. These mixtures were denatured by incubation at 98°C for 1.5 min and transferred to 65°C for 1.5 hs. For the second step of the SSH reaction, the two mixtures derived from the first step were mixed and 600 ng of digested driver DNA were added to the solution. The mixture was again denatured by incubation at 98°C for 1.5 min and incubated at 65°C for 14 hs. The resulting solution was diluted in 200 μL of Dilution Buffer (50 mM NaCl, 5 mM Hepes, pH 8.3, 0.2 mM EDTA) and incubated at 65°C for 10 more minutes, to eliminate non-specific hybridizations. One microliter (1 μL) from this final reaction was PCR amplified with the P01 initiator [73], in a 50 μL reaction, using the reagents from the Advantage 2 Polymerase Mix kit (BD Biosciences), according to the manufacturer's instructions. The cycling profile for the PCR reactions included 25 cycles at: 95°C for 30 seconds, 66°C for 30 seconds and 72°C for 1.5 min. The final reaction was then diluted 20 times in 10 mM Tris HCl, pH 7.5 and a 1-μL aliquot of this mixture was submitted to a second PCR reaction with initiators NP01 and NP02 (20 μM each) [73]. The reaction mixture was essentially as described above, but the cycling profile included only 10 cycles at: 95°C for 30 seconds, 68°C for 30 seconds and 72°C for 1.5 min. Finally, 3-μL aliquots from each SSH reaction were ligated to the pGEM-T vector (Promega), according to the manufacturer's instructions and this was used to transform competent *E. coli *cells, in order to generate the SSH libraries. Using 3 different restriction nucleases for the analysis of each strain, a total of 9,246 clones were isolated from 18 SSH libraries (Table 3).

### DNA sequencing and analysis

All SSH clones were selected and submitted to automated DNA sequencing using an ABI 3100 DNA analyzer, according to the manufacturer's instructions. Further analyses were performed for each individual strain separately. Thus, sequences from each group of strain-specific SSH libraries were trimmed, in order to exclude vector sequences and poor quality regions (Phred < 20), and aligned with the aid of CAP3 [74]. Sequences that remained as singlets, or resulted in small contigs with poor quality consensus sequences (Phred < 40) were excluded from further analyses. The consensus sequences from the remaining contigs were filtered against the genome of *Xf *strain 9a5c with the aid of the software cross_match [33], allowing the identification of stretches of DNA that were exclusive to the tested strains. The cross_match parameters used to filter both vector and *Xf *strain 9a5c sequences were minmatch = 15 and minscore = 20. These newly identified sequences were analyzed with GeneMark, [34] to search for new individual ORFs present in their structure. The sequences from all newly identified ORFs were submitted to GenBank and can be accessed through numbers ER935541 to ER935830.

Since some of these ORFs could be present in the genome of more than one tested strain, the redundancy of the dataset was reduced, submitting the predicted aminoacid sequences from all identified ORFs to a clusterization analysis with the aid of Blast_Clust [75] and ClustalW [76], using a Perl script specially developed for this purpose [77]. This allowed the distribution of the 290 ORFs originally identified by GeneMark into 135 Groups of Orthologous Elements (GOEs). Analyses of similarity against the GenBank have next been performed with Blastx, using the consensus sequences from each GOE as input, allowing the assignment of putative functions for each of these newly identified elements, as well as their distribution into the different functional categories originally described by Simpson and coworkers [15].

The rate at which novel nucleotides could be identified in the SSH clones was determined by submitting subsets of SSH sequences to analysis with PhedPhrap/CAP3/cross_match (9a5c), as described above. After processing each subset, the number of novel nucleotides identified in the resulting sequence was determined by counting the nucleotides in the assembled contigs. Each sequence subset was built choosing random sequences within the whole set of *Xf *SSH sequences. A Perl script was developed for such purpose [77].

## Authors' contributions

VSS and FBR carried out the microarray analyses, SSH and sequencing experiments. CSS and DCDR were responsible for sequence alignment and other bioinformatics analyses. AAS, HDCF and MAM were responsible for helping in conceiving the study, as well as *Xf *strain selection, maintenance and DNA extraction. LRN and RCO were responsible for conceiving the project, as well as most data interpretation, general coordination of the study and final manuscript elaboration. All authors read and approved the final manuscript.

## Supplementary Material

Additional File 1 List of ORFs found to be deleted or present in higher copy number in the tested strains, using as a reference, the genome of *Xf *strain 9a5c. Click here for file

Additional File 2 Distribution and sequence of the 290 newly identified ORFs throughout the genomes of the tested *Xf *strains. Click here for file

Additional File 3 Most relevant BLAST hits found for the consensus sequences of each GOE. Click here for file
