# Supplementary material for: Comparative genomic characterization of citrus-associated Xylella fastidiosa strains
Source: BMC Genomics. 2007 Dec 21;8:474. doi: 10.1186/1471-2164-8-474 (PMC2262912; doi:10.1186/1471-2164-8-474)
Supplement: Additional File 1 — List of ORFs found to be deleted or present in higher copy number in the tested strains, using as a reference, the genome of Xf strain 9a5c. [file 1471-2164-8-474-S1.PDF]

ORFs that are either deleted or present in higher copy number among the *Xylella fastidiosa strains*

Update: 17 October, 2007

	187b	36f	56a	912c	Cv21	Fb7
**Number of ORFs present in higher copy number in the genomes of strains used in this study**	306	39	81	109	41	00
**Number of ORFs found to be deleted in genomes of strains used in this study**	00	01	20	18	13	20

187b (higher copy) return to main table

XF0132	Copper resistance protein A precursor-copA
XF0133	COPB; copper resistance protein B precursor
XF0134	VALS OR HI1391; valyl-tRNA synthetase
XF0135	Hypothetical protein
XF0136	DNA polymerase III holoenzyme chi subunit - holC
XF0137	Hypothetical protein
XF0138	PEPA OR XERB OR CARP aminopeptidase A I
XF0140	Conserved hypothetical protein
XF0250	Conserved hypothetical protein
XF0258	4-keto-L-rhamnose reductase -rfbC - dTDP
XF0276	MPL; UDP-N-acetylmuramate-L-alanine ligase
XF0277	Conserved hypothetical protein
XF0294	Hypothetical protein
XF0296	Type I restriction-modification system specificity determinant
XF0297	MJ1220 - type I restriction-modification system DNA methylase
XF0298	Hypothetical protein
XF0299	Hypothetical protein
XF0300	Hypothetical protein
XF0324	Periplasmic iron-binding protein -afuA
XF0422	Exodeoxyribonuclease V gamma chain - RECC
XF0423	Exodeoxyribonuclease V beta chain - RECB OR RORA
XF0424	Hypothetical protein
XF0425	RECD - exodeoxyribonuclease V alpha chain
XF0430	DNAG OR DNAP OR PARB; DNA primase
XF0489	Hypothetical protein
XF0502	Conserved hypothetical protein
XF0503	Hypothetical protein
XF0504	Hypothetical protein
XF0505	Hypothetical protein
XF0506	Virulence-associated protein E
XF0507	Hypothetical protein
XF0508	Phage-related terminase large subunit - gp2
XF0510	Hypothetical protein
XF0511	Hypothetical protein
XF0512	Hypothetical protein
XF0513	Phage-related endolysin - lycV
XF0514	Hypothetical protein
XF0515	Hypothetical protein
XF0516	Hypothetical protein
XF0517	Hypothetical protein

XF0518	Hypothetical protein
XF0519	Hypothetical protein
XF0521	Conserved hypothetical protein
XF0522	Hypothetical protein
XF0523	Hypothetical protein
XF0524	Hypothetical protein
XF0526	Hypothetical protein
XF0527	Hypothetical protein
XF0528	Hypothetical protein
XF0529	Hypothetical protein
XF0535	Transposase OrfA
XF0547	YDGM ferredoxin II
XF0549	METG OR HI1276 methionyl-tRNA synthetase
XF0552	Conserved hypothetical protein
XF0553	Conserved hypothetical protein
XF0554	Conserved hypothetical protein
XF0556	Conserved hypothetical protein
XF0557	Electron transfer protein azurin I - az1
XF0560	SCF55.27 GMP synthase
XF0631	Phage-related integrase - int
XF0633	Hypothetical protein
XF0634	Hypothetical protein
XF0636	Hypothetical protein
XF0639	Hypothetical protein
XF0659	MESJ cell cycle protein
XF0668	FRPC; hemolysin-type calcium binding protein
XF0685	Phage-related protein
XF0687	Hypothetical protein
XF0713	Phage-related portal protein- gp4
XF0714	Hypothetical protein
XF0715	Hypothetical protein
XF0717	Hypothetical protein
XF0718	Conserved hypothetical protein
XF0719	Phage-related baseplate assembly protein- gpV
XF0721	Proteic killer suppression protein- higA
XF0723	Phage-related baseplate assembly protein- gpW
XF0724	Phage-related baseplate assembly protein- gpJ
XF0725	Phage-related tail protein- gpI
XF0726	Hypothetical protein
XF0727	FIR2; phage-related contractile tail sheath protein
XF0733	Phage-related tail protein-gpD
XF0754	Virulence protein - acvB
XF0755	XSEA; exodeoxyribonuclease VII large subunit
XF0757	Conserved hypothetical protein
XF0758	Conserved hypothetical protein
XF0759	AMIC; N-acetylmuramoyl-L-alanine amidase precursor
XF0760	MUTL - DNA mismatch repair protein MutL
XF0770	Hypothetical protein
XF0774	Hypothetical protein
XF0777	Membrane protein- ACTII-3
XF0813	Hypothetical protein
XF0820	Hypothetical protein
XF0830	Hypothetical protein

XF0831	CYSK - cysteine synthase
XF0832	CYSG - siroheme synthase
XF0833	CYSB OR HI1200 - transcriptional regulator (LysR family)
XF0834	Conserved hypothetical protein
XF0835	UBIH OR VISB - 2-octaprenyl-6-methoxyphenol hydroxylase
XF0840	BGA - beta-galactosidase
XF0842	Conserved hypothetical protein
XF0843	Conserved hypothetical protein
XF0844	Hypothetical protein
XF0845	Family 3 glycoside hydrolase - xylA
XF0868	Dihydrolipoamide dehydrogenase - lpdA or lpd
XF0882	YOAA - ATP-dependent helicase
XF0889	Hemagglutinin-like secreted protein- pspA
XF0890	Hypothetical protein
XF0891	Hypothetical protein
XF0892	Hypothetical protein
XF0893	Hypothetical protein
XF0894	Hypothetical protein
XF0895	Hypothetical protein
XF0896	Hypothetical protein
XF0897	Hypothetical protein
XF0928	Conserved hypothetical protein
XF0929	Conserved hypothetical protein
XF0950	RIBD OR RIBG; riboflavin-specific deaminase
XF0995	CYSS OR HI0078; cysteinyl-tRNA synthetase
XF0999	ARGG OR MJ0429; argininosuccinate synthase
XF1000	ARGE; acetylornithine deacetylase
XF1003	ASL; argininosuccinate lyase
XF1004	DR1827; glutamate 5-kinase
XF1005	PROA; gamma-glutamyl phosphate reductase
XF1011	FRPC; hemolysin-type calcium binding protein
XF1012	Conserved hypothetical protein
XF1015	Manganese transport protein- mntH2
XF1062	EDD 6-phosphogluconate dehydratase
XF1063	6-phosphogluconolactonase - pgl
XF1064	GLK glucose kinase
XF1114	Regulator of pathogenicity factors -RPFC
XF1118	MURD OR RV2155C OR MTCY270.13 UDP-N-acetylmuramoylalanine--D-glutamate ligase
XF1120	Bifunctional DGTP-pyrophosphohydrolase thiamine phosphate synthase - mutT or thiE1
XF1121	METF OR AQ_1429 5;10-methylenetetrahydrofolate reductase
XF1126	Conserved hypothetical protein
XF1229	DR0420 - ATP-dependent helicase
XF1241	Aconitate hydratase 1 - ACNA OR CAN
XF1243	Hypothetical protein
XF1252	Conserved hypothetical protein
XF1341	Copper homeostasis protein - cutC
XF1372	Hypothetical protein
XF1383	HRPA - helicase; ATP dependent
XF1423	PURL OR PURI phosphoribosylformylglycinamidine synthetase
XF1437	Thiol disulfide interchange protein - dsbA
XF1470	Conserved hypothetical protein
XF1499	CYSJ NADPH-sulfite reductase; flavoprotein subunit

XF1568	Hypothetical protein
XF1718	Phage-related integrase - int
XF1719	Hypothetical protein
XF1720	Hypothetical protein
XF1721	Hypothetical protein
XF1722	Hypothetical protein
XF1723	Sugar-phosphate dehydrogenase - yrpG
XF1726	LINC - 2;5-dichloro-2;5-cyclohexadiene-1;4-diol dehydrogenase
XF1727	ADH - NADP-alcohol dehydrogenase
XF1728	Transport protein - f451
XF1729	DR1890 - phenylacetaldehyde dehydrogenase
XF1730	YAFC - transcriptional regulator (LysR family)
XF1732	NAD(P)H-dependent 2-cyclohexen-1-one reductase - ncr
XF1733	AF0343 - tryptophan repressor binding protein
XF1735	Conserved hypothetical protein
XF1736	Hypothetical protein
XF1737	Conserved hypothetical protein
XF1738	Hypothetical protein
XF1739	Outer membrane protein - romA
XF1740	YLII - glucose dehydrogenase B
XF1741	Daunorubicin C-13 ketoreductase - dnrU
XF1742	Conserved hypothetical protein - drp35
XF1743	Esterase - est
XF1744	Oxidoreductase
XF1745	Conserved hypothetical protein
XF1747	Conserved hypothetical protein
XF1748	MJ0671 - 5-amino-6-(5-phosphoribosylamino)uracil reductase
XF1750	Conserved hypothetical protein
XF1751	Hypothetical protein
XF1753	Hypothetical protein
XF1754	Conserved hypothetical protein
XF1755	Conserved hypothetical protein
XF1756	Hypothetical protein
XF1758	Hypothetical protein
XF1759	Conserved hypothetical protein
XF1760	Hypothetical protein
XF1761	Hypothetical protein
XF1762	Conserved hypothetical protein
XF1764	Hypothetical protein
XF1766	Hypothetical protein
XF1767	Hypothetical protein
XF1768	YCJZ - transcriptional regulator (LysR family)
XF1769	Hypothetical protein
XF1770	Hypothetical protein
XF1771	Hypothetical protein
XF1772	Hypothetical protein
XF1773	Hypothetical protein
XF1774	HPAIIM DNA methyltransferase
XF1775	IS629 - reverse transcriptase
XF1776	DNA topoisomerase III - topB
XF1777	Hypothetical protein
XF1779	Single-stranded DNA binding protein- ssb
XF1780	Hypothetical protein

XF1783	Hypothetical protein
XF1784	Hypothetical protein
XF1785	SOJ; chromosome partitioning related protein
XF1786	Hypothetical protein
XF1787	Hypothetical protein
XF1788	Hypothetical protein
XF1929	Hypothetical protein
XF1931	PLSB OR HI0748-glycerol-3-phosphate acyltransferase
XF1934	HETI HetI protein
XF2035	Hypothetical protein
XF2036	Hypothetical protein
XF2038	PAIB; transcriptional regulator
XF2043	Hypothetical protein
XF2067	Conserved plasmid protein -yacB
XF2068	Hypothetical protein
XF2084	Component of multidrug efflux system - mexE
XF2108	Hypothetical protein
XF2109	Hypothetical protein
XF2112	Hypothetical protein
XF2114	Hypothetical protein
XF2115	Hypothetical protein
XF2117	Hypothetical protein
XF2118	Hypothetical protein
XF2119	Hypothetical protein
XF2120	Hypothetical protein
XF2121	Virulence-associated protein E - vapE
XF2123	Hypothetical protein
XF2125	Hypothetical protein
XF2132	Hypothetical protein
XF2179	Conserved hypothetical protein
XF2207	SC1C3.02; cationic amino acid transporter
XF2214	HISF - cyclase
XF2215	phosphoribosylformimino-5-aminoimidazole carboxamide ribotide isomerase
XF2216	HISH OR HI0472 amidotransferase
XF2217	HISB - imidazoleglycerolphosphate dehydratase histidinol-phosphate phosphatase bifunctional enzyme
XF2218	HISC - histidinol-phosphate aminotransferase
XF2219	HISD - histidinol dehydrogenase
XF2220	HISG - ATP phosphoribosyltransferase
XF2221	Conserved hypothetical protein - yecD
XF2223	THRC - threonine synthase
XF2224	THRB - homoserine kinase
XF2225	THRA OR THRA1 OR THRA2 - bifunctional aspartokinase homoserine dehydrogenase I
XF2233	DNAJ; DnaJ protein
XF2271	Hypothetical protein
XF2273	Conserved hypothetical protein
XF2276	Hypothetical protein
XF2290	Phage-related protein
XF2291	Phage-related protein
XF2292	Hypothetical protein
XF2294	KILA phage-related protein
XF2302	HEML glutamate-1-semialdehyde 2;1-aminomutase
XF2317	Hypothetical protein

XF2385	YEGN - acriflavin resistance protein D
XF2409	Conserved hypothetical protein
XF2410	Hypothetical protein
XF2417	LSPA lipoprotein signal peptidase
XF2418	ILES OR ILVS isoleucyl-tRNA synthetase
XF2419	RIBF riboflavin biosynthesis protein
XF2455	Heme ABC transporter ATP-binding protein - ccmA
XF2456	Heme ABC transporter membrane protein - ccmB
XF2457	Heme ABC transporter membrane protein - ccmC
XF2459	CYCJ c-type cytochrome biogenesis protein
XF2460	CYCK c-type cytochrome biogenesis membrane protein
XF2461	DSBE OR CCMG c-type cytochrome biogenesis protein thioredoxin
XF2462	CYCL c-type cytochrome biogenesis protein (16.3 kDa)-orf 2462
XF2463	Hypothetical protein
XF2470	CBBZC phosphoglycolate phosphatase
XF2471	UBIG OR PUFX 3-demethylubiquinone-9 3-methyltransferase
XF2472	Conserved hypothetical protein
XF2473	EFP elongation factor P
XF2474	Conserved hypothetical protein
XF2475	HI0380 tRNA rRNA methyltransferase
XF2477	BIOD dethiobiotin synthetase
XF2483	Conserved hypothetical protein
XF2486	Hypothetical protein
XF2487	Hypothetical protein
XF2488	Phage-related baseplate assembly protein - gpJ
XF2492	Phage-related baseplate assembly protein - gpV
XF2493	Conserved hypothetical protein
XF2494	Hypothetical protein
XF2497	Hypothetical protein
XF2522	Phage-related protein
XF2523	Phage-related protein
XF2538	PILC - fimbrial assembly protein
XF2549	Hypothetical protein
XF2551	Conserved hypothetical protein - At2g47390
XF2556	DNA ligase
XF2705	Conserved hypothetical protein
XF2721	Type I restriction-modification system endonuclease - hsdR1
XF2722	MJ1218; type I restriction-modification system specificity determinant
XF2723	Type I restriction-modification system DNA methylase - hsdM
XF2724	Type I restriction-modification system- hsdM
XF2734	Hypothetical protein
XF2735	Hypothetical protein
XF2740	Hypothetical protein
XF2741	Type I restriction-modification system specificity determinant - hsdS
XF2742	Type I restriction-modification system DNA methylase - hsdM
XF2744	Hypothetical protein
XF2745	Hypothetical protein
XF2745	Hypothetical protein
XF2747	Hypothetical protein
XF2748	Hypothetical protein
XF2762	Hypothetical protein
XF2764	Conserved hypothetical protein
XF2767	Hypothetical protein

XF2773	Hypothetical protein
XF2775	Hemagglutinin-like secreted protein - pspA
XF2777	Hypothetical protein

36f (higher copy) return to main table

XF0279	Hypothetical protein
XF0424	Hypothetical protein
XF0425	RECD - exodeoxyribonuclease V alpha chain
XF0508	Phage-related terminase large subunit - gp2
XF0510	Hypothetical protein
XF0514	Hypothetical protein
XF0516	Hypothetical protein
XF0518	Hypothetical protein
XF0523	Hypothetical protein
XF0526	Hypothetical protein
XF0757	Conserved hypothetical protein
XF0832	CYSG - siroheme synthase
XF0835	UBIH OR VISB - 2-octaprenyl-6-methoxyphenol hydroxylase
XF0845	Family 3 glycoside hydrolase - xylA
XF0889	Hemagglutinin-like secreted protein- pspA
XF0890	Hypothetical protein
XF0896	Hypothetical protein
XF1005	PROA; gamma-glutamyl phosphate reductase
XF1118	MURD OR RV2155C OR MTCY270.13 UDP-N-acetylmuramoylalanine--D-glutamate ligase
XF1744	Oxidoreductase
XF1752	Transcriptional regulator (LysR family)
XF1753	Hypothetical protein
XF1756	Hypothetical protein
XF1769	Hypothetical protein
XF1769	Hypothetical protein
XF1776	DNA topoisomerase III - topB
XF1785	SOJ; chromosome partitioning related protein
XF2037	Conserved hypothetical protein - bioF2
XF2114	Hypothetical protein
XF2121	Virulence-associated protein E - vapE
XF2456	Heme ABC transporter membrane protein - ccmB
XF2475	HI0380 tRNA rRNA methyltransferase
XF2487	Hypothetical protein
XF2492	Phage-related baseplate assembly protein - gpV
XF2723	Type I restriction-modification system DNA methylase
XF2735	Hypothetical protein
XF2736	Hypothetical protein
XF2742	Type I restriction-modification system DNA methylase - hsdM
XF2775	Hemagglutinin-like secreted protein - pspA

36f (deleted) return to main table

XF2663	Hypothetical protein

56a (higher copy) return to main table

XF0138	PEPA OR XERB OR CARP aminopeptidase A I
XF0298	Hypothetical protein

XF0422	Exodeoxyribonuclease V gamma chain - RECC
XF0425	RECD - exodeoxyribonuclease V alpha chain
XF0490	Hypothetical protein
XF0503	Hypothetical protein
XF0504	Hypothetical protein
XF0505	Hypothetical protein
XF0506	Virulence-associated protein E
XF0508	Phage-related terminase large subunit - gp2
XF0510	Hypothetical protein
XF0511	Hypothetical protein
XF0513	Phage-related endolysin - lycV
XF0514	Hypothetical protein
XF0516	Hypothetical protein
XF0517	Hypothetical protein
XF0518	Hypothetical protein
XF0519	Hypothetical protein
XF0521	Conserved hypothetical protein
XF0522	Hypothetical protein
XF0523	Hypothetical protein
XF0524	Hypothetical protein
XF0526	Hypothetical protein
XF0527	Hypothetical protein
XF0528	Hypothetical protein
XF0529	Hypothetical protein
XF0556	Conserved hypothetical protein
XF0715	Hypothetical protein
XF0718	Conserved hypothetical protein
XF0723	Phage-related baseplate assembly protein- gpW
XF0730	Phage-related tail protein-gpT
XF0759	AMIC; N-acetylmuramoyl-L-alanine amidase precursor
XF0879	Lipopolysaccharide biosynthesis protein - rfbU
XF0889	Hemagglutinin-like secreted protein- pspA
XF1012	Conserved hypothetical protein-YFHC
XF1723	Sugar-phosphate dehydrogenase - yrpG
XF1728	Transport protein - f451
XF1730	YAFC - transcriptional regulator (LysR family)
XF1736	Hypothetical protein
XF1737	SCM11.20c - conserved hypothetical protein
XF1738	Hypothetical protein
XF1741	Daunorubicin C-13 ketoreductase - dnrU
XF1742	Conserved hypothetical protein - drp35
XF1743	Esterase - est
XF1744	Oxidoreductase
XF1745	Conserved hypothetical protein
XF1747	Conserved hypothetical protein
XF1751	Hypothetical protein
XF1752	Transcriptional regulator (LysR family)
XF1753	Hypothetical protein
XF1756	Hypothetical protein
XF1763	Phage-related protein
XF1766	Hypothetical protein
XF1767	Hypothetical protein
XF1768	YCJZ - transcriptional regulator (LysR family)

XF1772	Hypothetical protein
XF1774	HPAIIM DNA methyltransferase
XF1776	DNA topoisomerase III - topB
XF1777	Hypothetical protein
XF1780	Hypothetical protein
XF1785	SOJ; chromosome partitioning related protein
XF2112	Hypothetical protein
XF2114	Hypothetical protein
XF2115	Hypothetical protein
XF2119	Hypothetical protein
XF2120	Hypothetical protein
XF2121	Virulence-associated protein E - vapE
XF2222	Histidyl-tRNA synthetase - hisS
XF2290	Phage-related protein
XF2417	LSPA lipoprotein signal peptidase
XF2479	Phage-related protein
XF2483	Conserved hypothetical protein
XF2485	FIR2 phage-related contractile tail sheath protein
XF2486	Hypothetical protein
XF2488	Phage-related baseplate assembly protein - gpJ
XF2492	Phage-related baseplate assembly protein - gpV
XF2522	Phage-related protein
XF2525	Phage-related DNA polymerase - dpoL
XF2538	PILC - fimbrial assembly protein
XF2735	Hypothetical protein
XF2775	Hemagglutinin-like secreted protein - pspA

56a (deleted) return to main table

XF0078	MRKD - fimbrial adhesin precursor
XF0497	Conserved hypothetical protein - Rv2514c
XF0667	Hypothetical protein
XF1160	RPMC - 50S ribosomal protein L29
XF1250	Arginine deaminase -rocF
XF1646	LPXD OR FIRA; UDP-3-O-(R-3-hydroxymyristoyl)-glucosamine N-acyltransferase
XF1664	Hypothetical protein
XF1665	Hypothetical protein
XF1707	Hypothetical protein
XF1708	Conserved hypothetical protein
XF1851	Serine protease
XF1859	Hypothetical protein
XF1861	Hypothetical protein
XF1863	Hypothetical protein
XF1880	Hypothetical protein
XF1884	Hypothetical protein
XF1885	Hypothetical protein
XFa0003	Topoisomerase I
XFa0004	Hypothetical protein
XFa0012	Conjugal transfer protein

912c (higher copy) return to main table

XF0133	COPB; copper resistance protein B precursor
XF0297	MJ1220 - type I restriction-modification system DNA methylase

XF0423	Exodeoxyribonuclease V beta chain - RECB OR RORA
XF0424	Hypothetical protein
XF0430	DNAG OR DNAP OR PARB; DNA primase
XF0489	Hypothetical protein
XF0490	Hypothetical protein
XF0503	Hypothetical protein
XF0504	Hypothetical protein
XF0505	Hypothetical protein
XF0506	Virulence-associated protein E
XF0507	Hypothetical protein
XF0508	Phage-related terminase large subunit - gp2
XF0510	Hypothetical protein
XF0511	Hypothetical protein
XF0513	Phage-related endolysin - lycV
XF0514	Hypothetical protein
XF0515	Hypothetical protein
XF0516	Hypothetical protein
XF0517	Hypothetical protein
XF0518	Hypothetical protein
XF0521	Conserved hypothetical protein
XF0522	Hypothetical protein
XF0523	Hypothetical protein
XF0524	Hypothetical protein
XF0526	Hypothetical protein
XF0527	Hypothetical protein
XF0528	Hypothetical protein
XF0715	Hypothetical protein
XF0719	Phage-related baseplate assembly protein- gpV
XF0723	Phage-related baseplate assembly protein- gpW
XF0754	Virulence protein - acvB
XF0755	XSEA; exodeoxyribonuclease VII large subunit
XF0757	Conserved hypothetical protein
XF0758	Conserved hypothetical protein
XF0759	AMIC; N-acetylmuramoyl-L-alanine amidase precursor
XF0832	CYSG - siroheme synthase
XF0835	UBIH OR VISB - 2-octaprenyl-6-methoxyphenol hydroxylase
XF0840	BGA - beta-galactosidase
XF0842	Conserved hypothetical protein
XF0843	Conserved hypothetical protein
XF0879	Lipopolysaccharide biosynthesis protein - rfbU
XF0882	YOAA - ATP-dependent helicase
XF0889	Hemagglutinin-like secreted protein- pspA
XF0928	Conserved hypothetical protein
XF0950	RIBD OR RIBG; riboflavin-specific deaminase
XF1002	AF2071; N-acetyl-gamma-glutamyl-phosphate reductase
XF1003	ASL; argininosuccinate lyase
XF1241	Aconitate hydratase 1 - ACNA OR CAN
XF1383	HRPA - helicase; ATP dependent
XF1719	Hypothetical protein
XF1723	Sugar-phosphate dehydrogenase - yrpG
XF1735	Conserved hypothetical protein
XF1737	Conserved hypothetical protein
XF1738	Hypothetical protein

XF1741	Daunorubicin C-13 ketoreductase - dnrU
XF1743	Esterase - est
XF1744	Oxidoreductase
XF1745	Conserved hypothetical protein
XF1750	Conserved hypothetical protein
XF1751	Hypothetical protein
XF1753	Hypothetical protein
XF1755	Conserved hypothetical protein
XF1756	Hypothetical protein
XF1769	Hypothetical protein
XF1772	Hypothetical protein
XF1773	Hypothetical protein
XF1774	HPAIIM DNA methyltransferase
XF1775	IS629 - reverse transcriptase
XF1776	DNA topoisomerase III - topB
XF1779	Single-stranded DNA binding protein- ssb
XF1780	Hypothetical protein
XF1784	Hypothetical protein
XF1785	SOJ; chromosome partitioning related protein
XF2112	Hypothetical protein
XF2114	Hypothetical protein
XF2115	Hypothetical protein
XF2120	Hypothetical protein
XF2121	Virulence-associated protein E - vapE
XF2123	Hypothetical protein
XF2125	Hypothetical protein
XF2207	SC1C3.02; cationic amino acid transporter
XF2223	THRC - threonine synthase
XF2224	THRB - homoserine kinase
XF2227	Conserved hypothetical protein
XF2416	Drug tolerance protein - lytB
XF2418	ILES OR ILVS isoleucyl-tRNA synthetase
XF2455	Heme ABC transporter ATP-binding protein - ccmA
XF2456	Heme ABC transporter membrane protein - ccmB
XF2469	Hypothetical protein
XF2481	Phage-related tail protein - gpX
XF2482	Phage-related protein
XF2486	Hypothetical protein
XF2494	Hypothetical protein
XF2497	Hypothetical protein
XF2522	Phage-related protein
XF2523	Phage-related protein
XF2538	PILC - fimbrial assembly protein
XF2705	Conserved hypothetical protein
XF2733	Hypothetical protein
XF2735	Hypothetical protein
XF2736	Hypothetical protein
XF2738	Hypothetical protein
XF2775	Hemagglutinin-like secreted protein - pspA
XFa0020	Hypothetical protein
XFa0027	plasmid maintenance protein
XFa0028	Hypothetical protein
XFa0033	Hypothetical protein

XFa0060	Plasmid replication protein

912c (deleted) return to main table

XF0078	MRKD - fimbrial adhesin precursor
XF0497	Conserved hypothetical protein
XF0665	Phosphohydrolase - orfU1
XF0666	Hypothetical protein
XF1167	RPLF - 50S ribosomal protein L6
XF1250	Arginine deaminase -rocF
XF1646	LPXD OR FIRA; UDP-3-O-(R-3-hydroxymyristoyl)-glucosamine N-acyltransferase
XF1664	Hypothetical protein
XF1665	Hypothetical protein
XF1707	Hypothetical protein
XF1708	Conserved hypothetical protein
XF1851	Serine protease
XF1859	Hypothetical protein
XF1861	Hypothetical protein
XF1863	Hypothetical protein
XF1880	Hypothetical protein
XF1884	Hypothetical protein
XF1885	Hypothetical protein

Cv21 (higher copy) return to main table

XF0485	Phage-related protein
XF0487	fimbrillin
XF0489	Hypothetical protein
XF0490	Hypothetical protein
XF0492	Hypothetical protein
XF0493	Hypothetical protein
XF0503	Hypothetical protein
XF0506	Virulence-associated protein E
XF0508	Phage-related terminase large subunit - gp2
XF0510	Hypothetical protein
XF0511	Hypothetical protein
XF0512	Hypothetical protein
XF0513	Phage-related endolysin - lycV
XF0514	Hypothetical protein
XF0515	Hypothetical protein
XF0516	Hypothetical protein
XF0517	Hypothetical protein
XF0518	Hypothetical protein
XF0519	Hypothetical protein
XF0521	Conserved hypothetical protein
XF0522	Hypothetical protein
XF0523	Hypothetical protein
XF0524	Hypothetical protein
XF0527	Hypothetical protein
XF0528	Hypothetical protein
XF0532	Hypothetical protein
XF0790	Conserved hypothetical protein
XF0889	Hemagglutinin-like secreted protein- pspA
XF1118	MURD OR RV2155C OR MTCY270.13 UDP-N-acetylmuramoylalanine--D-glutamate ligase

XF2112	Hypothetical protein
XF2114	Hypothetical protein
XF2115	Hypothetical protein
XF2118	Hypothetical protein
XF2119	Hypothetical protein
XF2120	Hypothetical protein
XF2123	Hypothetical protein
XF2494	Hypothetical protein
XFa0004	Hypothetical protein
XFa0022	Conserved hypothetical protein
XFa0027	Plasmid maintenance protein
XFa0028	Hypothetical protein

Cv21 (deleted) return to main table

XF0078	MRKD - fimbrial adhesin precursor
XF0497	Conserved hypothetical protein
XF1646	LPXD OR FIRA; UDP-3-O-(R-3-hydroxymyristoyl)-glucosamine N-acyltransferase
XF1664	Hypothetical protein
XF1665	Hypothetical protein
XF1707	Hypothetical protein
XF1708	Conserved hypothetical protein
XF1851	Serine protease
XF1859	Hypothetical protein
XF1861	Hypothetical protein
XF1863	Hypothetical protein
XF1880	Hypothetical protein
XF1885	Hypothetical protein

Fb7 (deleted) return to main table

XF0497	Conserved hypothetical protein
XF1707	Hypothetical protein
XF1863	Hypothetical protein
XF1880	Hypothetical protein
XF1885	Hypothetical protein
XF2040	Hypothetical protein
XFa0001	Transcriptional regulator
XFa0002	Conjugal transfer protein
XFa0003	Topoisomerase I
XFa0004	Hypothetical protein
XFa0012	Conjugal transfer protein
XFa0047	Nnickase
XFa0048	Hypothetical protein
XFa0051	Hypothetical protein
XFa0055	Conserved hypothetical protein
XFa0059	Plasmid replication partition protein
XFa0060	Plasmid replication protein
XFa0062	Hypothetical protein
XFa0063	Hypothetical protein
XFa0064	Hypothetical protein
