# Supplementary material for: Comparative genomic characterization of citrus-associated Xylella fastidiosa strains
Source: BMC Genomics. 2007 Dec 21;8:474. doi: 10.1186/1471-2164-8-474 (PMC2262912; doi:10.1186/1471-2164-8-474)
Supplement: Additional File 2 — Distribution and sequence of the 290 newly identified ORFs throughout the genomes of the tested Xf strains. [file 1471-2164-8-474-S2.PDF]

Distribution of the 135 newly identified GOEs throughout the genomes of the *Xylella fastidiosa* strains used in this study

Update: 17 October 2007

GOE #	Presumed Function	187b	56a	912c	Cv21	Fb7	36f
01	Plasmid encoded RepA protein	X	X		X		X
02	TrbL/VirB6 plasmid conjugal transfer protein	X	X	X	X	X	X
03	TrbL/VirB6 plasmid conjugal transfer protein		X	X			
04	TRAG protein	X				X	
05	CagE, TrbE, VirB component of type IV transporter system	X	X	X			X
06	CagE, TrbE, VirB component of type IV transporter system	X	X		X		
07	Probable conjugal transfer protein TraL	X	X	X		X	X
08	Phage-related integrase	X			X		X
09	Conjugal transfer protein TrbG/VirB9/CagX		X			X	X
10	CagE, TrbE, VirB component of type IV transporter system			X			X
11	Putative IS element transposase	X				X	
12	Conserved hypothetical protein			X	X		
13	Probable conjugal transfer protein TraL		X	X	X		X
14	Conjugation TrbI-like protein		X		X		
15	Conserved hypothetical protein		X	X			
16	Resolvase, N-terminal:Resolvase helix-turn-helix region		X		X		
17	Transcriptional regulator AbrB	X	X			X	
18	TRAG protein			X	X		X
19	Putative transposase TnA	X	X				
20	Helix-turn-helix motif	X	X	X	X	X	
21	No hit	X		X			
22	Adenylate kinase subfamily	X		X			X
23	Conserved hypothetical protein	X		X			
24	Plasmid-related exported protein			X		X	
25	Plasmid encoded RepA protein			X	X		
26	Lpx-Transferase hexapeptide repeat	X		X	X		
27	Conserved hypothetical protein			X	X		
28	No hit	X		X	X		
29	Helix-turn-helix motif:Peptidase S24, S26A and S26B			X			X
30	VirB8	X	X		X		
31	type IV secretory pathway, VirB3 family protein		X	X	X		
32	Type II secretion system protein E	X	X	X	X	X	X
33	Phage-related integrase			X			X
34	Phase-related integrase	X	X	X	X	X	
35	CagE, TrbE, VirB component of type IV transporter system	X	X	X		X	X
36	Plasmid encoded RepA protein	X		X			
37	CopG-like DNA-binding protein	X		X			
38	Type II secretion system protein E	X			X		X
39	No hit			X		X	
40	CagE, TrbE, VirB component of type IV transporter system	X	X	X	X	X	X

41	Type II secretion system protein E		X	X	X		X
42	Conjugal transfer protein TrbG/VirB9/CagX		X	X	X		
43	SpoVT/AbrB-like		X	X			
44	Conserved hypothetical protein				X		X
45	Methyltransferase, type III restriction-modification system	X	X			X	X
46	BRO, N-terminal			X		X	
47	Conserved hypothetical protein	X		X			
48	conjugation TrbI-like protein	X	X			X	X
49	No hit	X	X	X		X	
50	Conserved hypothetical protein	X	X	X		X	
51	Zonula occludens toxin	X		X			
52	Conserved hypothetical protein	X	X		X		
53	TRAG protein			X	X		
54	Conserved hypothetical protein			X	X		X
55	Conserved hypothetical protein			X	X		
56	Conserved hypothetical protein	X	X	X	X	X	X
57	No hit	X	X	X	X		X
58	No hit				X		X
59	Conserved hypothetical protein		X	X	X		X
60	Conserved hypothetical protein		X	X			X
61	Conserved hypothetical protein			X			X
62	Conserved hypothetical protein			X			X
63	Plasmid-related exported protein				X		X
64	Conserved hypothetical protein	X			X		
65	Plasmid-related exported protein	X	X				
66	Conjugation TrbI-like protein			X			X
67	Resolvase N-terminal domain	X		X			
68	Conserved hypothetical protein			X	X		
69	No hit			X	X		
70	Conserved hypothetical protein				X		X
71	Helix-turn-helix motif:Peptidase S24, S26A and S26B			X	X		
72	Conjugal transfer protein TrbG/VirB9/CagX	X		X			
73	TRAG protein		X				
74	RepB/MobA-like protein			X			
75	Conserved hypothetical protein			X			
76	Fimbrial protein pilin			X			
77	Putative transposase			X			
78	Conserved hypothetical protein			X			
79	No hit			X			
80	No hit			X			
81	No hit			X			
82	Filamentous phage Cf1c related protein			X			
83	Replication initiator-like protein			X			
84	No hit			X			
85	Conserved hypothetical protein			X			
86	Conserved hypothetical protein			X			
87	Conserved hypothetical protein			X			
88	Conserved hypothetical protein			X			
89	No hit			X			
90	No hit			X			
91	No hit			X			
92	Phage integrase			X			

93	Lpx-Acyl-[ACP]-UDP-N-acetylglucosamine			X			
94	VirB8			X			
95	ParB-like partition protein					X	
96	Conserved hypothetical protein					X	
97	Conjugal Transfer protein TrbA					X	
98	Type II secretion system protein E					X	
99	Relaxase/mobilization nuclease domain					X	
100	Helix-turn-helix motif:Peptidase S24, S26A and S26B					X	
101	Plasmid encoded RepA protein					X	
102	Conserved hypothetical protein					X	
103	Similar to Uncharacterized phage-associated protein					X	
104	No hit					X	
105	Conserved hypothetical protein					X	
106	Plasmid-related exported protein					X	
107	Putative plasmid conjugal transfer protein - TraJ					X	
108	Relaxase/mobilization nuclease domain					X	
109	No hit					X	
110	Conserved hypothetical protein					X	
111	Conserved hypothetical protein						X
112	Conserved hypothetical protein						X
113	Phage/plasmid primase P4, C-terminal						X
114	No hit						X
115	No hit						X
116	Helix-turn-helix motif:Peptidase S24, S26A and S26B	X					
117	No hit	X					
118	Methyltransferase, type III restriction-modification system	X					
119	No hit	X					
120	Peptidase S24, S26A and S26B		X				
121	No hit		X				
122	Conserved hypothetical protein		X				
123	No hit		X				
124	Helix-turn-helix motif:Peptidase S24, S26A and S26B		X				
125	TRAG protein		X				
126	Type I restriction system specificity protein				X		
127	Conserved hypothetical protein				X		
128	Conserved hypothetical protein				X		
129	Phage integrase				X		
130	Conserved hypothetical protein				X		
131	Conserved hypothetical protein				X		
132	Conserved hypothetical protein				X		
133	Conjugation TrbI-like protein				X		
134	No hit				X		
135	Conserved hypothetical protein				X		

GOE #01

return to main table

```
SSH33_56a        ------------------------------------------------------------
SSH40_Cv21       MFLGAGCWRGVRRDRVPRPRPRYSVVAAEVHGETPAITEAPGKENKSEHKKITAQESRLI 60
SSH21_187b       ----------------------------------------------------------LI 2
SSH16_36f        ----------------------------------------------------------LI 2
```

```
SSH33_56a           ------------------------------------------------------------
SSH40_Cv21          KTAAEIAMERPGDDDRAYMHSIMCQVGLPRSKVEGNSFERVSGAAALLIEAGKLWDGKRF 120
SSH21_187b          KTAAEIAMERPGDDDRAYMHSIMCQVGLPRSKVEGNSFERVSGAAALLIEAGKLWDGKRF 62
SSH16_36f           KTAAEIAMERPGDDDRAYMHSIMCQVGLPRSKVEGNSFERVSGAAALLIEAGKLWDGKRF 62


SSH33_56a           ---------MPRLILAWMNTYAVRCNTPVIPIGDSASEFLKILGKTPNGGVRGAFTTFKK 51
SSH40_Cv21          VQQSIPYGPMPRLILAWMNTYAVRCNTPVIPIGDSASEFLKILGKTPNGGVRGAFTTFKK 180
SSH21_187b          VQQSIPYGPMPRLILAWMNTYAVRCNTPVIPIGDSASEFLKILGKTPNGGVRGAFTTFKK 122
SSH16_36f           VQQSIPYGPMPRLILAWMNTYAVRCNTPVIPIGDSASEFLKILGKTPNGGVRGAFTTFKK 122
                         ***************************************************


SSH33_56a           QIQALSACRMTLGFNTNGHAHTYEGKPIKHFDAWLSGKEEQRPLWPGTVTFSDDYYQTLK 111
SSH40_Cv21          QIQALSACRMTLGFNTNGHAHTYEGKPIKHFDAWLSGKEEQRPLWPGTVTFSDDYYQTLK 240
SSH21_187b          QIQALSACRMTLGFNTNGHAHTYEGKPIKHFDAWLSGKEEQRPLWPGTLTFSDDYYPTLK 182
SSH16_36f           QIQALSACRMTLGFNTNGHAHTYEGKPIKHFDAWLSGKEEQRPLRPG--TCPGGRSNSVV 180
                     ******************************************** **  * ...   ::


SSH33_56a           LHAVPLDLRAYMELKRPRPRPRYFERPPGQVLDVSMM 148
SSH40_Cv21          LHAVPLDLRAYMELKRPRPRPRNGCLYMGRTTPIPH- 276
SSH21_187b          LHAVPLDLRAYIAWS----RPRYFM----EIESITA- 210
SSH16_36f           AAEVP-ELHKNAEKCD-------------NIRNLMTH 203
                    ** :*:                    .   :
```

GOE #02

return to main table

```
SSH18_36f           ---MDESAPITWLIDEINKIVSSGADAAASAIATTVTPLASICFGIYILLICLNYMRGAE 57
SSH15_Cv21          ---MDESAPITWLIDEINKIVSSGADAAASAIATTVTPLASICFGIYILLICLNYMRGAE 57
SSH02_56a           ---MDESAPITWLIDEINKIVSSGADAAASAIATTVTPLASICFGIYILLICLNYMRGAE 57
SSH16_187b          ---MDESAPITWLIDEINKIVSSGADAAASAIATTVTPLASICFGIYILLICLNYMRGAE 57
SSH28_Fb7           ---MDESAPITWLIDEINKIVSSGADAAASAIATTVTPLASICFGIYILLICLNYMRGAE 57
SSH57_912c          ---MDESAPITWLIDEINKIVSSGADAAASAIATTVTPLASICFGIYILLICLNYMRGAE 57
SSH22_912c          MDEMDESAPITWLIGEINKIVSSGADAAASTIATTITPLVSICFGIYILLICVNYMRGAE 60
                   ***********.***************:****:***.************:*******


SSH18_36f           TEPVIDFGIRCAGFAVIIGLGLNAANYTSLVIPLVTGVGSDLASAISGGHANVGTLDQLA 117
SSH15_Cv21          TEPVIDFGIRCAGFAVIIGLGLNAANYTSLVIPLVTGVGSDLASAISGGHANAGTLDQLA 117
SSH02_56a           TEPVIDFGIRCAGFAVIIGLGLNAANYTSLVIPLVTGVGSDLASAISGGHANAGTLDQLA 117
SSH16_187b          TEPVIDFGIRCAGFAVIIGLGLNAANYTSLVIPLVTGVGSDLASAISGGHANAGTLDQLA 117
SSH28_Fb7           TEPVIDFGIRCAGFAVIIGLGLNAANYTSLVIPLVTGVGSDLASAISGGHANAGTLDQLA 117
SSH57_912c          TEPVIDFGIRCAGFAVIIGLGLNAANYTSLVIPLVTGVGSDLASAISGGHANAGTLDQLA 117
SSH22_912c          TEPVIDFGIRCAGFAVVIGLGLNAANYTSLVIPMVTGIGSDLASAISGGSANAGTLDQLA 120
                    ***************:***************:***:*********** **.*******


SSH18_36f           LHYFNILDESYQSVKALKFPGSVGAMLIYGLKAIFILVGLIPFLVAATLCLIVADVGSVM 177
SSH15_Cv21          LHYFNILDESYQSVKALKFPGSVGAMLIYGLKAIFILVGLIPFLVAATLCLIVADVGSVM 177
SSH02_56a           LHYFNILDESYQSAQALKFPGSVGAMLIYGLKAIFILVGLIPFPAAG----------FRA 167
SSH16_187b          LHYFNILDESYQSVKALKFPGSVGAMLIYGLKAIFILVGLIPFLVAATLCLIVADVGSVM 177
SSH28_Fb7           LHYFNILDESYQSVKALKFPGSVGAMLIYGLKAIFILVGLIPFLVAATLCLIVADVGSVM 177
SSH57_912c          LHYFNILDESYQSVKALKFPGSVGAMLIYGLKAIFILVGLIPFLVAATLCLIVADVGSVM 177
SSH22_912c          LHYLNILDKSYKSLEKIYFPSNVAPLIIYAIKSIFILVGLIPFRAAAR-------AGTLS 173
                     ***:****:**:* : : **..*..::**.:*:********** .*.


SSH18_36f           VAMVGPMYFAFLIFPATRQYFSSWLNTAFSYALMPIFVAVIATISVGLSKQMFSANGTLN 237
SSH15_Cv21          VAMVGPMYFAFLIFPATRQYFSSWLNTAFSYALMPIFVAVIATISVGLSKQMFSANGTLR 237
SSH02_56a           AALAGTIRNLNCIH---------------------------------------------- 181
SSH16_187b          VAMVGPMYAVSSQK---------------------------------------------- 191
SSH28_Fb7           VAMVGPMYLGRDHAR--------------------------------------------- 237
SSH57_912c          VAMVGPMYLGRDHAISS---DASVFLIMAQYGIQTSAATTLFERPPGQVRFYVGRQQLYN 234
SSH22_912c          LPMLAL------------------------------------------------------ 179
                     .: .


SSH18_36f           D-----------------------------ISFTSAATTLFERPPGQVFRCSSRCSRMVQK 269
SSH15_Cv21          DRGRGKAVFLASMGNLTLLFLLKQVGAMASSLSAGGINVSMPGSANTLRHAAQAARLGAK 297
SSH02_56a           ------------------------------------------------------------
SSH16_187b          --------------------------------------------------ATRLSVPSYP 201
SSH28_Fb7           ----------------------------------------------------RLANLVFS 277
SSH57_912c          TR----------------------------------------------KGKAAGRSAFRFLT 250
SSH22_912c          ------------------------------------------------------------
```

```
SSH18_36f          HC------------------------------ 271
SSH15_Cv21         GIQQGGRTAINAGKAAGRWAENKFNSIRKAG 328
SSH02_56a          -------------------------------
SSH16_187b         AKRPCHASQ---------------------- 210
SSH28_Fb7          SLFSIIPP----------------------- 285
SSH57_912c         SRHSRVGA----------------------- 258
SSH22_912c         -------------------------------
```

GOE #03 return to main table

```
SSH74_912c      MPIFVAVIATISVGLSKQMFSANGTLNDISFKAVFLASMGNLTLLFLLKQVGAMASSLSA 60
SSH01_56a       MPIFVAVIATISVRLSKQMFSANGTLNDISFKAVFLASMGNLTLLFLLKQVGAMASSLSA 60
SSH23_912c      MPIFVAVIATISVGLSKAMLSTNGTLDNISFRSVFLASMGNLTLLFLLRQVGAMASSLSA 60
                ************* *** *:*:****::***::***************:***********

SSH74_912c      GGINVSMPGSANTLRHAAQAARLGAKGFE---RPPGQVPPSMLARLLVDGQRTNLIRSGK 117
SSH01_56a       GGINVSMPGSANTLRHAAQAARLGAKGIQQGGRTSAATTLLAWSRPRYENDPRWTNATGA 120
SSH23_912c      GGINVSMPGSANTLRNAAEATKLGAKILQQ--GGRASINAGKAAGRWAQNTFNSIRKAG- 117
                **************:**:*::**** ::       .       :    :.       :*

SSH74_912c      QAKL----------------------------- 121
SSH01_56a       VVQLEGLQECWPTKPISTATTDHDCLDRKGYKR 153
SSH23_912c      ---------------------------------
```

GOE #04 return to main table

```
SSH35_187b      MNKKKIAIAVLVLLLLAMVSVLVMLYMSSGLLVKYLNLGDLTPHLSLPLDLAKFGTKKEK 60
SSH25_Fb7       MNKKKIAIAVLVLLLLAMVSVLVMLYMSSGLLVKYLNLGDLTPHLSLPLDLAKFGTKKEK 60
                ************************************************************

SSH35_187b      GFGIIAMVVSVGLPVMLFGIIGYAALAPKKRELHGSARFATRRELVKSGLLQSDKPDDQY 120
SSH25_Fb7       GFGIIAMVVSVGLPVMLFGIIGYAALAPKKRELHGSARLATRRRLVQSGLLPSDKP---- 116
                **************************************:****.**:**** ****

SSH35_187b      PSILVGKQDKDFLFFRGQQFMFLAAPTRSGKGVGIVIPNLLHYRDSVVVLDIKGENFEIT 180
SSH25_Fb7       ------------------------------------------------------------


SSH35_187b      SGFRASVVAAEVRKD 195
SSH25_Fb7       ---------------
```

GOE #05 return to main table

```
SSH36_56a       MNLIIAVAAAAPAAMRPLPTATGYRFDSVVRPARAAASNSRGRGGRERLRAAARAGLEEM 60
SSH15_36f       ------------------------------------------------------------
SSH12_187b      -----------------------------------------------------------L 1
SSH50_912c      ------------------------------------------------------------


SSH36_56a       QIIVTQTLASYECEVLSIYDHNGHQFSQFYEFIAYLYNGFWERVPVTSLPLFQVVQTSAL 120
SSH15_36f       ---------------------------------------------------VVQTSAL 7
SSH12_187b      QSDLIVLIPSQPVDTLAWSRPR------------YLYNGFWERVPVTSLPLFQVVQTSAL 49
SSH50_912c      ------------------------------------------------------------


SSH36_56a       HHGYKLLETRFPNGGNRYSAFFDLKDFPEPTTRGKFNPLLELPFPFVMCLSFTFINTADS 180
SSH15_36f       HHGYKLLETRFPNGGNRYSAFFDLKDFPEPTTRGKFNPLLELPFPFVMCLSFTFINTADS 67
SSH12_187b      HHGYKLLETRFPNGGNRYSAFFDLKDFPEPTTRGKFNPLLELPFPFVMCLSFTFINTADS 109
SSH50_912c      ------------------------------------------------------------


SSH36_56a       IRLINQALNKMESAGDEATEQMKDMDFGKGAIMAGEVYFGELHGALAVYLPG----LKSR 236
SSH15_36f       IRLINQALNKMESAGDEATEQMKDMDFGKGAIMAGEVYFGELHGALAVYLGRDHAIAWSR 127
SSH12_187b      IRLINQALNKMESAGDEATEQMKDMDFGKGAIMAGEVYFGELHGALAVYLPG-------R 162
SSH50_912c      ----------MESAGDEATEQMKDMDFGKGAIMAGEVYFGETSAATTLFE---------R 41
                *******************************  .* :::          *

SSH36_56a       PKIGALRL------------------------ 244
SSH15_36f       PRY-MVRLPRSRKHLARKTNQSIKKLPPKNPA 158
SSH12_187b      PLIRAAARAGTGQNQP--TPNQWRNCSP---- 188
SSH50_912c      PPGQVQ-------------------------- 47
```

*

GOE #06

return to main table

```
SSH17_Cv21          MAFILKAKGLPFEMTSDNVLENQYDELNGLFLSLAKSTGSRLAVWAHIDHYAKTLKSNYE 60
SSH36_187b          ------------------------------------------------------------
SSH37_56a           ------------------------------------------------------------


SSH17_Cv21          FSYEWIRLFTEKYMARFEGESIFENSFYLTFILKPGMNDSLEECIRELEEMQIIVTQTLA 120
SSH36_187b          ------------------------------------------------------------
SSH37_56a           ------------------------------------------------------------


SSH17_Cv21          SYECEVLSIYDHNGHQFSQFYEFIAYLYNGFWERVPVTSLPLFQVVQTSALHHGYKLLET 180
SSH36_187b          ------------------------------------------------------------
SSH37_56a           ------------------------------------------------------------


SSH17_Cv21          RFPNGGNRYSAFFDLPPGQVKDFPEPTTRGKFNPLLELPFPFVMCLSFTFINTADSIRLI 240
SSH36_187b          ------------------------------------------------------------
SSH37_56a           ------------------------------------------------------------


SSH17_Cv21          NQALNKMESAGDEATEQMKDMDFGKGAIMAGEVYFGELHGALAVYGKTEKQAENRGATAL 300
SSH36_187b          ------------------------------------------------------------
SSH37_56a           ------------------------------------------------------------


SSH17_Cv21          ASLSGSCATRFVPATISAPETFFSMFPGNVKRRPRPMPKTTRNFLGLFSMNTYNSGKQYG 360
SSH36_187b          -------------------------------------------------MNTYNSGKQYG 11
SSH37_56a           ------------------------------------MPKTTRNFLGLFSMNTYNSGKQYG 24
                                                                 ***********

SSH17_Cv21          NPVGDGSAIMPLQTPAHGVYHLNFHYSPPDLDSHGEKVAGHLLITGATGVGKTTVQTVAL 420
SSH36_187b          NPVGDGSAIMPLQTPAHGVYHLNFHYSPPDLDSHGEKVAGHLLITGATGVGKTTVQTVAL 71
SSH37_56a           NPVGDGSAIMPLQTPAHGVYHLNFHYSPPDLDSHGEKVAGHLLITGATGVGKTTVQTVAL 84
                    ************************************************************

SSH17_Cv21          TFCSRWGNKLFAVDKDGSMRGFIEAVGGTYFRLASGEPTGL-------PRPRPR- 467
SSH36_187b          TFCSRWGNKLFAVDKDGSMRGFIEAVGGTYLR----------------PRPR--- 107
SSH37_56a           TFCSRWGNKLFAVDKDGSMRGFIEAVGGTYFRLASGELTGYSDRDHANPAARPRD 139
                    ******************************:*                * .*
```

GOE #07

return to main table

```
SSH07_56a           ------------------------------------------------------------
SSH23_36f           -------------LKIY---------------------PTEKVSEL-------------- 12
SSH30_187b          ------------------------------------------VYLP-------------- 4
SSH20_Fb7           ------------------------------------------------------------
SSH04_912c          MPKTTRNFLGLFSMNTYNSGKQYGNPVGDGSAIMPLQTPAHGVYHLNFHYSPPDLDSHGE 60


SSH07_56a           ------------------------------------------------------------
SSH23_36f           --------------AKTLAESPALPLKFISH--------SETIAKFERPPGQVPLSNYLI 50
SSH30_187b          --------------GRPLEHAPG---RHGRG--------NSVVA------AEVPLSNYLI 33
SSH20_Fb7           ------------------------------------------------------------
SSH04_912c          KVAGHLLITGATGVGKTTVQTVALTFCSRWGNKTCPGGRSNSSGRPARSSSFVPLSNYLI 120


SSH07_56a           ------MLKESGRNVFIHSVLTGGQALMDTLAGFKSLAEQADTNNIVIWLNEYFGAIEAN 54
SSH23_36f           ENNAISMLKESGRNVFIHSVLTGGQALMDTLAGFKSLAEQADTNNIVIWLNEYFGAIEAN 110
SSH30_187b          ENNAISMLKESGRNVFIHSVLTGGQALMDTLAGFKSLAEQADTNNIVIWLNEYFGAIEAN 93
SSH20_Fb7           ------MLKESGRNVFIHSVLTGGQALMDTLAGFKSLAEQADTNNIVIWLNEYFGAIEAN 54
SSH04_912c          ENNAISMLKESGRNVFIHSVLTGGQALMDTLAGFKSLAEQADTNNIVIWLNEYFGAIEAN 180
                      ******************************************************

SSH07_56a           GKTFTEMKVYTENAHKVRGIVPRP-RPR-YVRKGYGSDGIKKAYVFERPSGQVLTSL 109
SSH23_36f           GKTFTEMKVYTENAHKVRGIVPRP-RPR-YVRKGYGSDGIKKAYVFERPPGQVLTSL 165
SSH30_187b          GKTFTEMKVYTENAHKVRGIVPRP-RPR-YVRKGYGSDGIKKAYVF----------- 137
SSH20_Fb7           GKTFTEMKVYTENAHKVRGIVPRP-RPR-YVRKGYGSDGIKKAYVFRSSERY----- 104
SSH04_912c          GKTFTEMKVYTENAHKVRGIVRIIGRNQDTFGKDMEVMASKKTYVFRSSERY----- 232
```

```
                     *********************    * :  . *.    . **:***
```

GOE #08 [return to main table]

```
SSH01_187b          ------------------------MEARSGAHERGTIGHVITHYLRSTDFQQLAPRTQED 36
SSH31_Cv21          VLEIPRKAVAVAGPSALLSDLHSIMEARSGAHERGTIGHVITHYLRSTDFQQLAPRTQED 60
SSH10_36f           ------------------------MEARSGAHERGTIGHVITHYLRSTDFQQLAPRTQED 36
                                        ************************************

SSH01_187b          YHRQVEIVRSYKTTMGMTLDRLHIARMSPAIIQRIVEKIAVGNGEIKGKPTKANHLFRFL 96
SSH31_Cv21          YHRQVEIVRSYKTTMGMTLDRLHIARMSPAIIQRIVEKIAVGNGEIKGKPTKANHLFRFL 120
SSH10_36f           YHRQVEIVRSYKTTMGMTLDRLHIARMSPAIIQRIVEKIAVGNGEIKGKPTKANHLFRFL 96
                    ************************************************************

SSH01_187b          RLVFSWGIRHGHCMDNPAKGVRQAPERKRDGMPTLEAFAAILQFATRRGRLKAHTAGSVP 156
SSH31_Cv21          RLVFSWGIRHGHCMDNPAKGVRQAPERKRDGMPTLEAFAAILQFATRRGRLKAHTAGSVP 180
SSH10_36f           RLVFSWGIRHGHCMDNPAKGVRQAPERKRDGMPTLEAFAAILQFATRRGRLKAHTAGSVP 156
                    ************************************************************

SSH01_187b          RYIAPLMYLGRDHAIPAAVAAGSVRSRSVDSLRAGCPLTHNSFSLIKSLICPHCYGEDVW 216
SSH31_Cv21          RYIAPLMYLP---------------GRPLEMLNC-------------------------- 199
SSH10_36f           RYIAPLSSGRP--------------ARYDATVAS--RQTLRQLPDVTELAG-------RA 193
                    ******                   .*    : .

SSH01_187b          VSILDTVNTVGILIDSE 233
SSH31_Cv21          -----------------
SSH10_36f           MPKLQRSNDGNSL---- 206
```

GOE #09 [return to main table]

```
SSH19_36f           ------------------------------------------------------------
SSH30_56a           ------------------------------------------------------------
SSH29_Fb7           MIRWICIRTPGRMAAGLAWSRPRYVLEIPRKAVAVAGPSALLSDLHSIMEARSGAHERGT 60


SSH19_36f           ------------------------------------------------------------
SSH30_56a           ------------------------------------------------------------
SSH29_Fb7           IGHVITHYLRSTDFQQLAPRTQEDYHRQVEIVRSYKTTMGMTLDRLHIARMSPAIIQRIV 120


SSH19_36f           ------------------------------------------------------------
SSH30_56a           ---------------------------------------------------VFFKALPDG 9
SSH29_Fb7           EKIAVGNGEIKGKPTKANHLFRFLRLVFSWGIRHGHCMAARFRAAARAGLPVFFKVLPDG 180


SSH19_36f           ----LNSNIDPEHKQTVVLHEVIRTVRARLGDQVIEIINRAYKLPKFNETGTSVPGAVRA 56
SSH30_56a           TEALLNSNIDPEHKQTVVLHEVIRTVRARLGDQVIEIINRAYKLPKFNETGTSVPGAVRA 69
SSH29_Fb7           TEALLNSNIDPEHKQTVVLHEVIRTVRARLGDQVIEIINRAYKLPKFNETGTSVPGAVRA 240
                    ********************************************************

SSH19_36f           DRDI 60
SSH30_56a           DRDI 73
SSH29_Fb7           DRDI 244
                    ****
```

GOE #10 [return to main table]

```
SSH04_36f           LFDVSVVAAEVLAMDAADFKYLEAAKAQAGTHPDQWVPVYKALRKKGRENVTQPVTVKE 59
SSH28_912c          ----MADDIAVLAMDAADFKYLEAAKAQAGTHPDQWVPVYKALRKKGRENVTQPVTVKE 55
                     .        *************************************************
```

GOE #11 [return to main table]

```
SSH31_187b          -------------------------------MVGLDAGVTKLATLSDGTVYHPVNSFKAN 29
SSH13_Fb7           VTYGHIAWSRPRYVSIQTEYEVADPVHNAESMVGLDAGVTKLATLSDGTVYHPVNSFKAN 60
                                           *****************************

SSH31_187b          QRKLAILQRQLSRKVKFSTNWQKQKRKIQNLHSHIANIRRDYLHKLTSEISKNHAMIVIE 89
SSH13_Fb7           QRKLAILQRQLSRKVKFSTNWQKQKRKIQNLHSHIANIRRDYLHKLTSEISKNHAMIVIE 120
                    ************************************************************

SSH31_187b          DLKVSNMSRSAKGTCPGGRSTMG---ESMLPAVWPRDSSVGPAEVLATTAIRCDDLFLLL 146
```

```
SSH13_Fb7           DLKVSNMSRSAIERPPGQVLKIGLIRVAQLTSVR-AAARAGTTDRQRNRCITATNSYRQK 179
                    ***********    **   .:*    : *.:*    : .*.::    . .* . : :

SSH31_187b          HERLM- 151
SSH13_Fb7           RYKSKQ 185
```

GOE #12

return to main table

```
SSH15_912c      ------------------------------------------MHFIKNYDARQITKMISE 18
SSH23_Cv21      LKIYPTEKVSELAKTLAESPALPLKFISHSETIAKTSKHIRDMHFIKNYDARQITKMISE 60
                                                          ******************

SSH15_912c      NGIKITLKEIKSILQNKEKKSQ 40
SSH23_Cv21      NGIKITLKEIKRILQNKEKKSQ 82
                *********** **********
```

GOE #13

return to main table

```
SSH08_56a           ------------------------------------------------------------
SSH05_912c          ------------------------------------------------------------
SSH46_Cv21          MLKESGRNVFIHSVLTGGQALMDTLAGFKSLAEQADTNNIVIWLNEYFGAIEANGKTFTE 60
SSH24_36f           ------------------------------------------------------------


SSH08_56a           --------------------------------------------------MAKQRIKTVQ 10
SSH05_912c          --------------------------------------------------MAKQRIKTVQ 10
SSH46_Cv21          MKVYTENAHKVRGIVRIIERNQDTFGKDMEVMASKKLTFSEVLSGTDFTLMAKQRIKTVQ 120
SSH24_36f           --------------------------------------------------MAKQRIKTVQ 10
                                                                  **********

SSH08_56a           RELNEQLDAIGF 22
SSH05_912c          RELNEQLDAIGF 22
SSH46_Cv21          RELNEQLDAIGF 132
SSH24_36f           RELNEQLDAIGF 22
                    ************
```

GOE #14

return to main table

```
SSH14_56a           LKPELASNNASLESDLIEKKKAEIKKKQEEEQAQIKAIEEAQAQARIQAETLAAANAAKR 60
SSH34_Cv21          LKPELASNNASLESDLIEKKKAEIKKKQEEEQAQIKAIEEAQAQARIQAETLAAANAAKR 60
                    ************************************************************

SSH14_56a           AQGDSAQVQQQGNQPPRPPTPMERKMAGSVLLENTGAKPKTDAPLSENTSSSEKEQQQRE 120
SSH34_Cv21          AQGDSAQVQQQGNQPPRPPTPMERKMAGSVLLENTGAKPKTDAPLSENTSSSEKEQQQRE 120
                    ************************************************************

SSH14_56a           VQARMAAMGMNQGGVQQLTPVGQSEGTDGLAGRLKPTVLQARLAAKLPNLDYLLKRGTTI 180
SSH34_Cv21          VQARMAAMCMNQGGVQQITPVDQSEVTDELAGRLKHTVLQARFGCKAA------------ 168
                    ******** ********:***.*** ** ****** ******:..* .

SSH14_56a           PCALKTGIDTTLPFVILAWSRPRYPAYTQWCKESGVYALSKVRFLGELERCVPKFGKKVT 240
SSH34_Cv21          ------------------------------------------------------------


SSH14_56a           KETVGAGKRREFTVIQGIELTAADL 265
SSH34_Cv21          -------------------------
```

GOE #15

return to main table

```
SSH41_56a       MPVFIAQLLTGLFSLLKTKLGYFIMAAFVWLGINWGSYHFVITPIVKQIYIYMDNVGVAE 60
912c26          -------------------LAAFAYHDCVRESDGQDYINRIERPPRQVIYIYMDNVGVAE 41
                                *. *     *  . . .  : :  *  : ************

SSH41_56a       GSLGEMAMSGLAILNFDRAVSMIISAYVARFAVLNGRLYLFKRGFGATPGAAP 113
912c26          GSLGEMAMSGLAILNFDRAVSMIISAYVARFAVLNGRLYLFKRGFGATPGAAP 94
                *****************************************************
```

GOE #16

return to main table

```
SSH28_Cv21          MSTLTVTARGQVTFRKEVLQHLGIKPGERIELDLLPDGRFERPPGQVLTVWRLDRLGRSM 60
SSH19_56a           -----------------------------------------------------------M 1
                                                                       *
```

```
SSH28_Cv21          TELVAIVNDLASRGVTFESLTEHIDTSSASGKFAFHLFSALAEFERNTIKERTRAGLAAA 120
SSH19_56a           TELVAIVNDLVSRGVTFESLTEHIDTSSASGKFAFHLFSALAEFERNTIKERTRAGLAAA 61
                    **********.*************************************************

SSH28_Cv21          RARGRMGGRPAKVTPKAKREMKALYTSQEVSVKDICTRYNITRSTFYRVVLERDYTANGK 180
SSH19_56a           RARGRMGGRPAKVTPKAKREMKALYTSQEVSVKDICTRYNITRSTFYRVVLERDYTANGK 121
                    ************************************************************

SSH28_Cv21          A 181
SSH19_56a           A 122
                    *
```

GOE #17 return to main table

```
SSH18_56a           MSTLTVTARGQVTFRKEVLQHLGIKPGERIELDLLPDGRAELKAAQPKAHSRNAGVPLAA 60
SSH14_Fb7           MSTLTVTARGQVTFRKEALQHLGIKPGERIELDLLPDGRAERGRGRGLAWIIQPLTTPRV 60
SSH34_Fb7           MSTLTVTARGQVTFRKEVLQHLGIKPGERIELDLLPDGRAACSRPLGRGD---------- 50
SSH28_187b          MSTLTVTARGQVTFRKEVLQHLGIKPGERIELDLLPDGRAELKAAQPKGSFQELRGFLKG 60
                    *****************.**********************        .

SSH18_56a           EIRAAARAGTPSTLSMSRPVTKRSPIK- 87
SSH14_Fb7           KTMLPAHQVAARFRAAARAGTT------ 82
SSH34_Fb7           ----------------------------
SSH28_187b          KTNGARLSIEEINDAIAEAGTLAGSGDA 88
```

GOE #18 return to main table

```
SSH20_912c      --------VVARAG---------------------LYDETGAQTMLTNMALQVAYAPRDD 31
SSH09_36f       MTEEGIFVVVAWSRPRYNMRLLLIFQSKSQIKDRKLYDETGAQTMLTNMALQVAYAPRDD 60
SSH35_Cv21      ----MRLLLIFQSK--------------SQIKDRKLYDETGAQTMLTNMALQVAYAPRDD 42
                        ::  :                      *************************

SSH20_912c      DDAKDYSEMIGYMTEKGLSKSRQLGLKSGRSESESDQRRAVLLPQEVKAIGQFKEIISME 91
SSH09_36f       DDAKDYSEMIGYMTEKGLSKSRQLGLKSGRSESESDQRRAVLLPQEVKAIGQFKEIISME 120
SSH35_Cv21      DDAKDYSEMIGYMTEKGLSKSRQLGLKSGRSESESDQRRAVLLPQEVKAIGQFKEIISME 102
                ************************************************************

SSH20_912c      NMAPALVDKIFWYQEPIFQARANLPHPDVPDQSDLVNTIEPDLGRDHANPA------AKR 145
SSH09_36f       NMAPALVDKIFWYQEPIFQARANLPHPDVPDQSDLVNTIEPAPPSPTLDFA------ERP 174
SSH35_Cv21      NMAPALVDKIFWYQEPIFQARANLPHPDVPDQSDLVNTIEPAPPSPTLDFAGAIEEQALP 162
                *****************************************       : *

SSH20_912c      PGACRAFE-----RPPGQVSRTDRDRST-------- 168
SSH09_36f       PGQVRARNPQKGCRGRGTVCTTFRSAFHHGSKVWRP 210
SSH35_Cv21      TGATFEVG-FSPKRNALEVVMSFVLGARLNYWWLHT 197
                .*           *    *  :
```

GOE #19 return to main table

```
SSH19_56a       --MHVHLVFV----AKYR--------RKVFDLDAIEKLRSYFASVCVDFDVELVEMDGEC 46
SSH20_187b      VIAGVSPCTCPGGRSKSRGQGGREHGRKVFDLDAIEKLRSYFASVCVIFDVELVEMDGEC 60
                    *         :* *        ********************* ************

SSH19_56a       DHVHLLINYPPKLAISNLVNSLKGVSSRLLRRDRPDIALHYYYKGVLWTPSYFASSCGGA 106
SSH20_187b      DHVHLLINYPPKLAISNLVNSLKGVSSRLLRRDRPDIALHYYYKGVLWTPSYFASSCGGA 120
                ************************************************************

SSH19_56a       PISIIRQYIQQQQTPS 122
SSH20_187b      PISIIRQYIQQQQTPS 136
                ****************
```

GOE #20 return to main table

```
SSH46_187b      MSEKMTVSCGNVFEDLGFPPDEAAAMLARETLLIALEKELRKRGKKQQELADELGVPRTR 60
SSH43_Cv21      MSEKMTVSCGNVFEDLGFPPDEAAAMLARETLLIALEKELRKRGKKQQELADELGVPRTR 60
SSH39_56a       MSEKMTVSCGNVFEDLGFPPDEAAAMLARETLLIALEKELRKRGKKQQELADELGVPRTR 60
SSH60_912c      MSEKMTVSCGNVFEDLGFPPDEAAAMLARETLLIALEKELRKRGKKQQELADELGVPRTR 60
SSH41_Fb7       MSEKMTVSCGNVFEDLGFPPDEAAAMLARETLLIALSFNHR--------LLFSLSVVAAE 52
                ************************************. : *        *  .*.*  :.

SSH46_187b      ISEVMHLKTDRFSVDKLVSLLHRAGNSSGRPGRYKLAHPSVKTPAKSDSLKPELASNNAS 120
```

```
SSH43_Cv21      ISEVMHLKTDRFSVDKLVSLLHRAGNSGGR-GR----------------------GTSGS 97
SSH39_56a       ISEVMHLKTDRFSVDKLVSLLHRAGKRVEIRVR--------------------------- 93
SSH60_912c      ISEVMHPQTDRFSVDKLVSLLHRAVERPPGQVLLY--------------------FLAIA 100
SSH41_Fb7       VHGVC------------------------------------------------------- 57
                :  *

SSH46_187b      LESDLIEKKKAEIKKKQEEEQAQIKAIEEAQAQARIQAETLAAANAAKRAQGDSAQVQQQ 180
SSH43_Cv21      FE---------------------------------------------------------- 99
SSH39_56a       ------------------------------------------------------------
SSH60_912c      L----------------------------------------------------------- 101
SSH41_Fb7       ------------------------------------------------------------


SSH46_187b      GNQPPRPPTPMERKMAGSVLLENTGAKPKTDAPLSENTSSSEKEQQQREVQARMAAMGMN 240
SSH43_Cv21      ------------------------------------------------------------
SSH39_56a       ------------------------------------------------------------
SSH60_912c      ------------------------------------------------------------
SSH41_Fb7       ------------------------------------------------------------


SSH46_187b      QGGVQQLTPVGQSEGTDGLAGRLKPTVLQARLAAKLPNLDYLLKRGTTSVVAAEVHQLQR 300
SSH43_Cv21      ------------------------------------------------------------
SSH39_56a       ------------------------------------------------------------
SSH60_912c      ------------------------------------------------------------
SSH41_Fb7       ------------------------------------------------------------


SSH46_187b      SWT 303
SSH43_Cv21      ---
SSH39_56a       ---
SSH60_912c      ---
SSH41_Fb7       ---
```

GOE #21 [return to main table]

```
SSH55_912c          MNSEIQVSLRVDWKANSQVATKENAFSRFVTELVEVFSLGIGHSGSARCTCPGGRSNAAR 60
SSH26_187b          MNSEIQVSLRVDWKANSQVATKENAFSRFVTELVEVFSLGIGHSGSARCTCPGGRSNSVV 60
                    **********************************************************:.

SSH55_912c          A------- 61
SSH26_187b          AAEVQEKR 68
                    *
```

GOE #22 [return to main table]

```
SSH28_36f           VLVERIAGRAQAESREDDTPDAVRKRLQVYNDCTAPVIGFYQQRGILAWARPRYLYKIDG 60
SSH52_912c          ------------------------------------------------------------
SSH06_187b          ------------------------------------------------------------


SSH28_36f           LADWAKKPEILAAFPYVGQITEDAGKKEQQHGVKLTSVGWEAKGLDN 107
SSH52_912c          LADWAKKPEILAAFPYVGQITEDAGKKEQQHGVKLTSVGWEAKGLDN 47
SSH06_187b          LADWAKKPEILAAFPYVGQITEDAGKKEQQHGVKLTSVGWEAKGPDN 47
                    ******************************************** **
```

GOE #23 [return to main table]

```
SSH51_912c          VPRRITQTYTEVDGKFYAKDSNRVMFEDKGEKLATSTTNKDAVADMVAYAKAKQWDSLKL 60
SSH42_187b          VPRRITQTYTEVDGKFYAKDSNRVMFEDKGEKLATSTTNKDAVADMVAYAKAKQWDSLKL 60
                    ************************************************************

SSH51_912c          SGSQEFRREAWLQAESQGIKTQGYTPKQTDLAALETLRQERSTNSITPLQERKTERQAST 120
SSH42_187b          SGSQEFRREAWLQAESQGIKTQGYTPKQTDLAALETLRQERSTNSITPLQERKTERPPST 120
                    ******************************************************** .**

SSH51_912c          SAATTL------------------------------------------------------ 126
SSH42_187b          AAAPRHDMNKNQAAMHVEASKFIATNMQALQNQPNMADKSVEDLTKLAYWRGIVAEENKL 180
                    :**.

SSH51_912c          ----------------------------------------------------
SSH42_187b          QPKAVQDEAIARFDKQAADPQFLKRLNQETEPKIHDRTTERVQQRDTHEQSL 232
```

GOE #24

return to main table

```
SSH18_Fb7      ------------------------------------------------------------
SSH29_912c     MSFTGILKKTRVPISAFVFIFAGTAYAQTFQVYDAANISTSIQNHVESIAKWKQQFEQLK 60


SSH18_Fb7      ------------------------------------VTVLSDVKKTSAYATERSKYPTSG 24
SSH29_912c     QQIDQHQKQYEAITGSRGMGALLDNSALKASLPSDWKQVLSDVKKTSAYATERSKYPTSG 120
                                                     **********************

SSH18_Fb7      NLQKTNAMYDVIASQDVIMSDLYSKANRRLNLIQSLTAQIDSANDPAAKADL-ANRLINE 83
SSH29_912c     NLQKTNAMYDVIASQDVIMSDLYSKANRRLNLIQSLTAQIDSANDPAAKDLPGATTLINE 180
               *************************************************    *.  ****

SSH18_Fb7      QNAIQANQNLVTILQAKQKQGLEIASQAAVEELSCKEFKRSGC 126
SSH29_912c     QNAIQANQNLVTILQAKQKQELEIASQAAVEELSCKEFKRSGC 223
               ******************** **********************
```

GOE #25

return to main table

```
SSH41_Cv21     ----------------------------------------------------VLYWSNLR 8
SSH18_912c     VTLLETSAATTLSPGRVRSTCVHIWPGQVSALAMDVYIWAAQRLYRIEGRPVVLYWSNLR 60
                                                                   ********

SSH41_Cv21     NQFGQEYTGKEADKNFKKKFLPALRAMLAVYPEARIKQVTGGIMLIASPPPIPFKPS 65
SSH18_912c     NQFGQEYTGKEADKNFKKKFLPALRAMLAVYPEARIKQVTGGIMLIASPPPIPFKPS 117
               *********************************************************
```

GOE #26

return to main table

```
SSH64_912c     --MKIVSLAPEVPRPRPRYFNYRFSEIAPAKNWINPDGIVGGIVATSADIHPSAWILRNT 58
SSH30_Cv21     LYLGRDHAIRAAARAGTGIFNYRFSEIAPAKNWINPDGIVGGIVATSADIHPSAWILRNT 60
SSH37_187b     ----LAAEIRAAARAGTGIFNYRFSEIAPAKNWINPDGIVGGIVATSADIHPSAWILRNT 56
                          ..*.  . *****************************************

SSH64_912c     VIYPDVIIGKRVYIGHKTTIKQCAIINEDTHITDSCRIGVCASIGGRSNIGSHSDINDAV 118
SSH30_Cv21     VIYPDVIIGKRVYIGHKTTIKQCAIINEDTHITDSCRIGVCASIGGRSNIGSHSDINDAV 120
SSH37_187b     VIYPDVIIGKRVYIGHKTTIKQCAIINEDTHITDSCRIGVCASIGGRSNIGSHSDINDAV 116
               ************************************************************

SSH64_912c     SIGESVSIGDFVKIFNNAVLGKNARIRDTVLIGEHVTIGAEAIVDHKAKIEHGASIGERA 178
SSH30_Cv21     SIGESVSIGDFVKIFNNAVLGKNARIRDTVLIGEHVTIGAEAIVNHKAKIEHGASIGERA 180
SSH37_187b     SIGESVSIGDFVKIFNNAVLGKNARIRDTVLIGEHVTIGAEAIVNHKAKIEHGASIGERA 176
               ********************************************:***************

SSH64_912c     VIECYVPRPRPRYSV---VAAEV------------------------------------- 198
SSH30_Cv21     VIECYVPRPRPRYSV---VAAEVRLQRAS----------FDVI-------------NHVR 214
SSH37_187b     VIECYVPRPRPRYHAPGRMAAGFRAAARAGTFVASWGWLFSLIGVPMILFVKIICTNDDK 236
               ************* .   :** .

SSH64_912c     ----------------------------------------------------
SSH30_Cv21     STYSLAKPAKWKAYHQTSWPMSWQ--KLSPSSSNFLGKFRPLQPFG--SVF- 261
SSH37_187b     AIRILMLEVKWSLLKALNGNAKYHGGTMAIAPTTYGRKLKNVKRYFKKTVCG 288
```

GOE #27

return to main table

```
SSH47_Cv21     ---------------------MNKLDENAATLAHDARYAQHTRSELAKAAYFRGFHEKAS 39
SSH12_912c     LICACSRPRPGGRYHAIRAAARAGLDENAATLVHDARYAQHTRSELAKAAYFRGFHEKAS 60
                                       ********.***************************

SSH47_Cv21     EFKGIAPDFSKYDATVASRQTLRQLPDVTELAGRAMPKLQRSNDGNSL 87
SSH12_912c     EFKGIAPDFSKYDATVASRQTLRQLPDVTELAGRAMPKLQRSNDGNSL 108
               ************************************************
```

GOE #28

return to main table

```
SSH06_Cv21         --------VVAAEV-YEVALEVLGQSRQPFMQAIYEEKQKSVPSQVFIRYCENRLAALDE 51
SSH01_912c         MLDLGINSVDAAEV-YEVALEVLGQSRQPFMQAIYEEKQKSVPSQVFIRYCENRLAALDE 59
SSH49_187b         ---MLDLGINSGSISYEVALEVLGQSRQPFMQAIYEEKQKSVLSQVFIRYCENRLAALDE 57
                           : :..: *************************** *****************

SSH06_Cv21         IQETLQPTDLATIERILTKGDLVFKVQ 78
```

```
SSH01_912c          IQETLQPTDLATIERILTKGDLVFKVQ 86
SSH49_187b          IQETLQPTDLATIERILTKGDLVFKVQ 84
                    ***************************
```

GOE #29 return to main table

```
SSH54_912c          MATAGTTLAGICRRYRQNLTTAPELLVPRPRPRYSVVAAEVLRIVSDNQDKDSEGRRIYQ 60
SSH33_36f           -----------------------------------VVAAEVLRIVSDNQDKDSEGRRIYQ 25
                                                       *************************

SSH54_912c          DEVVPPKEMGTVCVLGRVIMKIGDGGL 87
SSH33_36f           DEVVPPKEMDTVCVLGRVIMKIGDGGL 52
                    *********.*****************
```

GOE #30 return to main table

```
SSH19_187b          ---------MIEWGRASTSFPITALQCTCQGGRSKSRGQAAGSMVRAAAR---------- 41
SSH24_56a           -------------------------------------------LVRAAAR---------- 7
SSH14_Cv21          MSTKEQAPVLNKLARLPQTFVEAAVDFEKSKIEEIKRSRKIAWIIASVATVICSVSILAF 60
                                                           :: :.*

SSH19_187b          ------------------AGTGATTVLRSVRDTKDHYDEVVSVVAAESQYVRTCEGYDWF 83
SSH24_56a           ------------------AGT--------------------------------CEGYDWF 17
SSH14_Cv21          LVALLTRSEPEPTILQVDKSTGATTVLRSVRDTKDHYDEVVVKY-WLAQYVRTCEGYDWF 119
                                   .*                                    *******

SSH19_187b          TINDQFNACKLMSDGDVAKEYDSKVDAPGSPLKVLADKGKIVVGIVSIAFLGDTAQVRFT 143
SSH24_56a           TINDQFNACKLMSDGDVAKEYDSKVDAPGSPLKVLADKGKIVVGIVSIAFLGDTAQVPRP 77
SSH14_Cv21          TINDQFNACKLMSDGDVAKEYDSKVDAPGSPLKVLADKGKIVVGIVSIAFLGDTAQVRFT 179
                    *********************************************************  .

SSH19_187b          TEKLSASGENLDNSPVRKWIATIAFQFKPGLMTEQQRLINPLGFKVATYRVDPEVIQ 200
SSH24_56a           RPR------------------------------------------------------ 80
SSH14_Cv21          TEKLSASGENLDNSPVRKWIATIAFQFKPGLMTEQQRLINPLGFKVATYRVDPEVIQ 236
```

GOE #31 return to main table

```
SSH18_Cv21          ------------------------------------------------------------
SSH35_912c          ------------------------------------------------------------
SSH05_56a           VGVGAIAQAIQQVKYLQQQLTQMKNQLDSMNGDRGMAGLLSGQNRNYLPTDWNSAMNMLN 60


SSH18_Cv21          ------------------------------------------------------------
SSH35_912c          ------------------------------------------------------------
SSH05_56a           SGGGGSFGSLASAAQQIKQAQSVLSSSDLSRLSPQMQQYLDQVRNVSASQQALGQQAYAT 120


SSH18_Cv21          ----------MTDEQS----------------PEEYISYNGLGRSP-------------M 21
SSH35_912c          ----------MTDEQS----------------PEEYISYNGLGRSP-------------M 21
SSH05_56a           ASQRVNLLQTLTNQISSAIDPKAVWDLQARIQSEQLQLQNDQSRLPSVTQLTQAQSVATS 180
                              :*:: *                .*:    *. .* *

SSH18_Cv21          IWGIPYMAGLAI---MCLSLLGGLLLGTFVASWGWLFSLIGVPMILFVKIICTNDDKAIR 78
SSH35_912c          IWGIPYMAGLAI---MCLSLLGGLLLGTFVASWGWLFSLIGVPMILFVKIICTNDDKAIR 78
SSH05_56a           KWPTNYVRKPAVPRPLAAGFRAAARAGTFVASWGWLFSLIGVPMILFVKIICTNDDKAIR 240
                     *   *:   *:   :. .: ..   **********************************

SSH18_Cv21          ILMLEVKWSLLKALNGNAKYHGGTMAIAPTTYGRKLKNVR--AAARAGKKTVCG---- 130
SSH35_912c          ILMLEVKWSLLKALNGNAKYHGGTMAIAPTTYGRKLKNVKRYFTCPGGRSNSSGRPGR 136
SSH05_56a           ILMLEVKWSLLKALNGNAKYHGGTMAIAPTTYGRKLKNVK-----RYFKKTVCG---- 289
                    ***************************************:        :.. .*
```

GOE #32 return to main table

```
SSH10_Fb7           MRMKPDHIFITELRGDETWDYLMALKSGHSGSVTSIHANDCRGALYKIGSYIKQSEVGQT 60
SSH35_56a           MRMKPDHIFITELRGDETWDYLMALKSGHSGSVTSIHANDCRGALYKIGSYIKQSEVGQT 60
SSH37_36f           MRMKPDHIFITELRGDETWDYLMALKSGHSGSVTSIHANDCRGALYKIGSYIKQSEVGQT 60
SSH29_187b          MRMKPDHIFITELRGDETWDYLMALKSGHSGSVTSIHANDCRGALYKIGSYIKQSEVGQT 60
SSH47_912c          MRMKPDHIFITELRGDETWDYLMALKSGHSGSVTSIHANDCRGALYKIGSYIKQSEVGQT 60
SSH08_Cv21          ----------------------MALKSGHSGSVTSIHANDCRGALYKIGSYIKQSEVGQT 38
                                          **************************************
```

```
SSH10_Fb7          LDFNYIMQEVITTIDVVVFFEKTHLKELYLGRDHVIRAAARAGTSG-------------- 106
SSH35_56a          LDFNYIMQEVITTIDVVVFFEKTHLKELYLR--------PR------------------- 93
SSH37_36f          LDFNYIMQEVITTIDVVVFFEKTHLKELYLGRDHAIRAAARAGTVAGKEPRNSIAASGYR 120
SSH29_187b         LDFNYIMQEVITTIDVVVFFEKTHLKELYLGRDHAIPAAVAAGS---------------- 104
SSH47_912c         LDFNYIMQEVITTIDVVVFFEKTHLKELYLGRDHAKRHSSTFEE---------------- 104
SSH08_Cv21         LDFNYIMQEVITTIDVVVFFERPPGQNCTLTP---------------------------- 70
                   *********************:.   ::   *

SSH10_Fb7          ------------------------------------------------------------
SSH35_56a          ------------------------------------------------------------
SSH37_36f          IRQSRGFFVPVRFHGGRVTDKKPRKGEEVRLSGCRFLASRLLRVRRLETSPRGHLDQPGD 180
SSH29_187b         ------------------------------------------------------------
SSH47_912c         ------------------------------------------------------------
SSH08_Cv21         ------------------------------------------------------------


SSH10_Fb7          ------------------------------------------------------------
SSH35_56a          ------------------------------------------------------------
SSH37_36f          IFMSQSIIPFDFHSHAVRVVMRDGNPWFVATDVMGALDYAATSNPARVTEHIPSEWKGVN 240
SSH29_187b         ------------------------------------------------------------
SSH47_912c         ------------------------------------------------------------
SSH08_Cv21         ------------------------------------------------------------


SSH10_Fb7          ------------------------WPVANLRV-- 114
SSH35_56a          ----------------------------------
SSH37_36f          PIHTLGGEQKLLCLAEPGLYLPGLRAAARAGTFC 274
SSH29_187b         ------------------------RAAARAGTE- 113
SSH47_912c         -------------------------PPQFLE--- 110
SSH08_Cv21         ----------------------------------
```

GOE #33

return to main table

```
SSH66_912c         MTLTEANATEHGILSNRRKRSRDNITRWTPRLRQAWAELLAHRAAVLKRNCLPQRLHPQD 60
SSH03_36f          MTLTEANATEHGILSNRRKRSRDNITRWTPRLRQAWAELLAHRAAVLKRNCLPQRLHPQD 60
                   ************************************************************

SSH66_912c         RYLVLAESGHLLTKSGLDTAWQRLMALAIQEKVILDKQRFTLHGIKHRGITDSTCPGGRS 120
SSH03_36f          RYLVLAESGHLLTKSGLDTAWQRLMALAIQEKVILDKQRFTLHGIKHRGITDSTA----- 115
                   ******************************************************.

SSH66_912c         KGRYSRYGAWTALAEA 136
SSH03_36f          ----------------
```

GOE #34

return to main table

```
SSH72_912c         MTLTEANATEHGILSNRRKRSRDNITRWTPRLRQAWAELLAHRAAVLKRNCLPQRLHPQD 60
SSH03_36f          MTLTEANATEHGILSNRRKRSRDNITRWTPRLRQAWAELLAHRAAVLKRNCLPQRLHPQD 60
                   ************************************************************

SSH72_912c         RYLVLAESGHLLTKSGLDTAWQRLMALAIQEKVILDKQRFTLHGIKHRGITDSTCPGGRS 120
SSH03_36f          RYLVLAESGHLLTKSGLDTAWQRLMALAIQEKVILDKQRFTLHGIKHRGITDSTA----- 115
                   ******************************************************.

SSH72_912c         KGRYSRYGAWTALAEA 136
SSH03_36f          ----------------
SSH72_912c         MTLTEANATEHGILSNRRKRSRDNITRWTPRLRQAWAELLAHRAAVLKRNCLPQRLHPQD 60
SSH03_36f          MTLTEANATEHGILSNRRKRSRDNITRWTPRLRQAWAELLAHRAAVLKRNCLPQRLHPQD 60
                   ************************************************************

SSH72_912c         RYLVLAESGHLLTKSGLDTAWQRLMALAIQEKVILDKQRFTLHGIKHRGITDSTCPGGRS 120
SSH03_36f          RYLVLAESGHLLTKSGLDTAWQRLMALAIQEKVILDKQRFTLHGIKHRGITDSTA----- 115
                   ******************************************************.

SSH72_912c         KGRYSRYGAWTALAEA 136
SSH03_36f          ----------------
```

GOE #35

return to main table

```
SSH38_187b         ------------------------------------------------------------
SSH06_56a          ------------------------------------------------------------
SSH49_912c         MILFVKIICTNDDKAIRILMLEVKWSLLKALNGNAKYHGGTMAIAPTTYGRKLNSVVAAE 60
```

```
SSH16_Fb7           --------------MATLNITAEQWRLLQQLTVVN------------------SKMLSAI 28
SSH07_36f           --------------MATLNITAEQWRLLQQLTVVN------------------SKMLSAI 28


SSH38_187b          -----------------------------MAFILKAKGLPFEMTSDNVLENQYDELNGLF 31
SSH06_56a           -----------------------------MAFILKAKGLPFEMTSDNVLENQYDELNGLF 31
SSH49_912c          VKKQFAGEEQLPPYNYHVRDTIVSLEDGRMAFILKAKGLPFEMTSDNVLENQYDELNGLF 120
SSH16_Fb7           LKKQFAGEEQLPPYNYHVRDTIVSLEDGRMAFILKAKGLPFEMTSDNVLENQYDELNGLF 88
SSH07_36f           LKKQFAGEEQLPPYNYHVRDTIVSLEDGRMAFILKAKGLPFEMTSDNVLENQYDELNGLF 88
                                                 *******************************

SSH38_187b          LSLAKSTGSRLAVWAHIDHYAKTLKSNYEFSYEWIR---LFTEKYLGR------------ 76
SSH06_56a           LSLAKSTGSRLAVWAHIDHYAKTLKSNYEFSYEWIR---LFTEKYLGR------------ 76
SSH49_912c          LSLAKSTGSRLAVWAHIDHYAKTLKSNYEFSYEWIR---LFTEKYMARFEGESIFENSFY 177
SSH16_Fb7           LSLAKSTGSRLAVWAHIDHYAKTLKSNYEFSYEWIRPGRHGRGISSGR------------ 136
SSH07_36f           LSLAKSTGSRLAVWAHIDHYAKTLKSNYEFSYEWIR---LFTSVVAAE------------ 133
                    ************************************          ..

SSH38_187b          -DHAIRAAALAGLAVLMAHARQMLIMTVSPMMS--------------------------- 108
SSH06_56a           -DHAT------------------------------------------------------- 80
SSH49_912c          LTFILKPGMNDSLEECIRELEEMQIIVTQTLASYECEVLSIYDHNGHQFSQFYEFIAYLY 237
SSH16_Fb7           ------PGRYPAYTQWCKESGVYALSKVRFLGE--------------------------- 163
SSH07_36f           ------VWQYQIWSTVLKEY---------------------------------------- 147


SSH38_187b          RTLRRRIKI---------KTRIKIRGRLFLE----------------------------- 130
SSH06_56a           ---GRRISS---------GRPGRYLYRIIL------------------------------ 98
SSH49_912c          NGFWERVPVTSLPLFQVVQTSALHHGYKLLETRFPNGGNRYSAFFDLPRPRPRYTGKIQP 297
SSH16_Fb7           --LERCVPKFR------NKRVARTVDGKRRE----------------------------- 186
SSH07_36f           --LAGCF----------------------------------------------------- 152
                      .

SSH38_187b          ---VSDAMCRPSVPVIR--- 144
SSH06_56a           --------------------
SSH49_912c          PVRVTVSFCHVLVIYIHQYG 317
SSH16_Fb7           -LVVIQGIGLIDAGI----- 200
SSH07_36f           --AVTDQI------------ 158
```

GOE #36

[return to main table]

```
SSH68_912c          MPQSTKENNKRYKPSSGADVALSKLRKLTGTHLIHTSSLIHLNKQAPTKNDQVPRPRPRY 60
SSH10_187b          MPQSTKENNKRYKPSSGADVALSKLRKLTGTHLIHTSSLIHLNKQAPTKNDQVPRPRPRY 60
                ************************************************************

SSH68_912c          S----------------------SGRPGRYTVHPEHVASRHQKITN------- 84
SSH10_187b          SVVAAEVFDLLLMRWAFLCDGWKQEKAKKSKPQPPQNLLQQKKYPNIISALNI 113
                *                          . :. : . :* :   :::* .*
```

GOE #37

[return to main table]

```
SSH27_187b           MVSVSLRLPDEVTQRLENLAALTGRSKTFYMVEAIREHIDDLEDLYLAECELEAIRSGKS 60

SSH27_187b      QTVPRPRPRYMLPAAWPRDSAARASQLGSVPHQGA 95
```

GOE #38

[return to main table]

```
SSH40_187b          MTIRKPSSSRFSLTDYADSGRLRPVLAGLSDEIQPWEAELLQLVQKGDFVRFFELAVEHR 60
SSH29_36f           MTIRKPSSSRFSLTDYADSGRLRPVLAGLSDEIQPWEAELLQLVQKGDFVRFFELAVEHR 60
SSH37_Cv21          MTIRKPSSSRFSLTDYADSGRLRPVLAGLSDEIQPWEAELLQLVQKGDFVRFFELAVEHR 60
                    ************************************************************

SSH40_187b          LNIVTVGGTGSGKTTFSKCLIDLYPTSCRLFTIEDAHELTTPKHPNSVPRPRPR------ 114
SSH29_36f           LNIVTVGGTGSGKTTFSKCLIDLYPTSCRLFTIEDAHELTTPKHPNSVPRPRPRYSSGRP 120
SSH37_Cv21          LNIVTVGGTGSGKTTFSKCLIDLYPTSCRLFTIEDAHELTTPKHPNS-------YS---- 109
                    ***********************************************

SSH40_187b          ------------------------------------------------------------
SSH29_36f           GRYRCVRHHLDEFVRQFRWALNSRAEWLREQRVRAETLTDIQRAARFYYLQKLAFGGKVV 180
SSH37_Cv21          -------------------------------VVAAEVLP----AGQWR------------ 122
```

```
SSH40_187b           ---------------------------------
SSH29_36f            NQHFGFVTCGTSAATTLFERPPGQVPTIFLISL 213
SSH37_Cv21           --TYGFKPLPTSR----YAAKP-------QLSL 142
```

GOE #39

return to main table

```
SSH26_Fb7            MPSMVFPPGFFVKLFSILLSACLVIRVFPARLIFAASFSWLIPDL-GRDHAKYTNTPIHQ 59
SSH43_912c           MPSMVFPPGFFVKLFSILLSACLVIRVFPARLIFAASFSWLIPDLPGRPLAN----PVAM 56
                     ********************************************* **  *:    *:

SSH26_Fb7            YTNTPIHQYTNTPIHQYTNTPIHQYTNTR 88
SSH43_912c           AVERPPGQV-------------------- 65
                     .:  *  *
```

GOE #40

return to main table

```
SSH32_56a            ---------------------------------------------------------VGA 3
SSH38_Cv21           ---------------------------------------------------------VGA 3
SSH09_Fb7            ---------------------------------------------------------VGA 3
SSH08_187b           VSTCPGGRSNHAPGRLAAG-LAWSRPRYFRLASGEPTGLNPFQLPDTPQNRNFLYDLVGA 59
SSH21_36f            ---------------MRGFIEAVGG-TYFRLASGEPTGLNPFQLPDTPQNRNFLYDLVGA 44
                                                                             ***

SSH32_56a            CGRKVGQESTAEDTKDIKQAVDNVFAMPFKLRRFGVLLQSIPDHGENCLARRLADWCYGE 63
SSH38_Cv21           CGRKVGQESTAEDTRDIKQAVDNVFAMPFKLRRFGVLLQSIPDHGENCLARRLADWCYGE 63
SSH09_Fb7            CGRKVGQESTAEDTKDIKQAVDNVFAMPFKLRRFGVLLQSIPDHGENCLARRLADWCYDE 63
SSH08_187b           CGRKVGQESTAEDTKDIKQAVDNVFAMPFKLRRFGVLLQSIPDHGENCLARRLADWCYGE 119
SSH21_36f            CGRKVGQESTAEDTKDIKQAVDNVFAMPFKLRRFGVLLQSIPDHGENCLARRLADWCYGE 104
                     **************:*******************************************.*

SSH32_56a            IEGRYAYALDNPQNNFDWENLKRVGFDVSDFLVAGHPATEP-ILAYLFHLKTLMQRDGEL 122
SSH38_Cv21           IEGRYAYALDNPQNNFDWENLKRVGFDVSDFLVAGHPATEP-ILAYLFHLKTLMQRDGEL 122
SSH09_Fb7            IEGRYAYALDNPQNNFDWENLKRVGFDVSDFLVAGHPATEP-ILAYLFHLKTLMQRDGEL 122
SSH08_187b           IEGRYAYALDNPQNNFDWENLKRVGFDVSDFLVAGHPATEP-ILAYLFHLKTLMQRDGEL 178
SSH21_36f            IEGRYAYALDNPQNNFDWENLKRVGFDVSDFLVAGHPATEP-ILAYLFHLKTLMQRDGEL 163
                     *********************** *..***********: .   :***************

SSH32_56a            LATVVEEFWLPLQYPTTADQILDSLKTGRRRGEFILLVSQSPEDVIKSPLLPAVLQQTPT 182
SSH38_Cv21           LATVVEEFWLPLQYPTTADQILDSLKTGRRRGEFILLVSQSPEDVIKSPLLPAVLQQTPT 182
SSH09_Fb7            LATVVEEFWLPLQYPTTADQILDSLKTGRRRGEFILLVSQSPEDVIKSPLLPAVLQQTPT 182
SSH08_187b           LATVVEEFWLPLQYPTTADQILDSLKTGRRRGEFILLVSQSPEDVIKSPLLPAVLQQTPT 238
SSH21_36f            LATVVEEFWLPLQYPTTADQILDSLKTGRR------------------------------ 193
                     ******************************

SSH32_56a            KIFLPNPDAEYTTPDGGGYSRFTQIKEFQKLKKLGLQSRMFIVKQGSQSSLVKLNLSGMA 242
SSH38_Cv21           KIFLPNPDAEYTTPDGGGYSRFTQIKEFQKLKKLGLQSRMFIVKQGSQSSLVKLNLSGMA 242
SSH09_Fb7            KIFLPNPDAEYTTPDGGGYSRFTQIKEFQKLKKLGLQSRMFIVKQGSQSSLVKLNLSGMA 242
SSH08_187b           KIFLPNPDAEYTTPDGGGYSRFTQIKEFQKLKKLGLQSRMFIVKQGSQSSLVKLNLSGMA 298
SSH21_36f            ------------------------------------------------------------

SSH32_56a            DDIAVPRPRPRY------------------------- 254
SSH38_Cv21           DDIAVPRQ----------------------------- 250
SSH09_Fb7            DDIAVVAAE---------------------------- 251
SSH08_187b           DDIAVPGRAAATDTSRLAITLAWSRPRYNQPQLQEIY 335
SSH21_36f            -------------------------------------
```

GOE #41

return to main table

```
SSH29_56a            MSLTLLNDVPQKQVSQVDRSLSVRELMRKYGISEALMANITEFAINRPGSYWVEDAAGWR 60
SSH12_36f            MSLTLLNDVPQKQVSQVDRSLSVRELMRKYGISEALMANITEFAINRPGSYWVEDAAGWR 60
SSH32_Cv21           ------------------------------------MANITEFAINRPGSYWVEDAAGWR 24
SSH24_912c           --------------------------MRKYGISEALMANITEFAINRPGSYWVEDAAGWR 34
                                                         ************************

SSH29_56a            EVPNESLSLSHLQGLATATAVFNQKKLDRDNPIASLTLPDGERCQVVPR----------- 109
SSH12_36f            EVPNESLSLSHLQGLATATAVFNQKKLDRDNPIASLTLPDGERCQVVPARAAAR---IAW 117
SSH32_Cv21           EVPNESLSLSHLQGLATATAVFNQKKLDRDNPIASLTLPDGERCQVVPR----------- 73
SSH24_912c           EVPNESLSLSHLQGLATAIAWSTGRGLDRDNPIASLTLPDGERCQVVLPPACENGTVSMT 94
                     ****************** *  . : *********************

SSH29_56a            PRPR-------------------------------------------------------- 113
```

```
SSH12_36f           SRPRYSKQFRAAARAGTSSSGAVVR-FWRYLRHIPASVVPAVAIRRKKTACHKADSSALS 176
SSH32_Cv21          PRPR-------------------------------------------------------- 77
SSH24_912c          IRKPSSSRFSLTDYADSGRLRPVLAGLSDEIQPWEAELLQLVQKGDFVRFFETSGRTSLE 154
                     *

SSH29_56a           -------------------
SSH12_36f           A-DIPRTPIS--TVLVTF- 191
SSH32_Cv21          -------------------
SSH24_912c          HRDGGRHRVRENDVLKVPD 173
```

GOE #42

return to main table

```
SSH25_56a           MKKLPVLLLACVTHFAYANDPRIRTEIYDKSSVYNLHTQIGRASLIQ-LEADESLEISPS 59
SSH13_Cv21          MKKLPVLLLACVTHFAYANDPRIRTEIYDKSSVYNLHTQIGRASLIQ-LEADESLEISPS 59
SSH76_912c          MKKLPVLLLACVTHFAYANDPRIRTEIYDKSSVYNLHTQIGRASLIQTCPGDESLEISPS 60
                    ***********************************************   .*********

SSH25_56a           SLLGIGDAKAWDLG------VLRGR----------------------------------R 79
SSH13_Cv21          SLLGIGDAKAWDLGVRGNNIVLPRP----------------------------------R 85
SSH76_912c          SLLGIGDAKAWDLGVRGNNIVLKPTQKMPQTNVIVVTNKRTYSFELLATAKDSMPTYILR 120
                    **************      **                                     *

SSH25_56a           HLFSTASSDDQ-----HGADY------------------ 95
SSH13_Cv21          PRYSVVAAEVKRLRLLHPLQIVIWP-------------- 110
SSH76_912c          FRYPDTEASKADLGRDHATDNCRSPRRKNDHQYRLLLAW 159
                      :. . :.       *  :
```

GOE #43

return to main table

```
SSH13_912c          MHTTNLRKVGGSIMLAVPPAFLDQLHLEVGATVGLAVTDGRLVIEPVLHPQYLPGRPLDS 60
SSH28_56a           MHTTNLRKVGGSIMLAVPPAFLDQLHLEVGATVGLAVTDGRLVIEPVLHPQYLGRDHATS 60
                    *****************************************************      *

SSH13_912c          VVAAEVYLFATPNRTPIYYKNINI 84
SSH28_56a           VVAAEVHR--KRGR---------- 72
                    ******:   . .*
```

GOE #44

return to main table

```
SSH05_187b          -------------LRSRT---------SAATTL--------------------------- 11
SSH11_Cv21          MPRRSL-----SFFHSVA---------NVQRTFNPLVAG-----SIPARPTKLGREH--A 39
SSH11_36f           MHTTNLRKVGGSIMLAVPPAFLDQLHLEVGATVGLAVTDGRLVIEPVLHPQYLGRDHAIA 60
                                 : : .         ..  *.

SSH05_187b          FERPPGQVRPTNPAEAKISETKAKTLETWSQVVRNLEVTGQTDLAKSVMALMRDADKSTS 71
SSH11_Cv21          FERPPGQVRPTNPAEAKISETKAKTLETWSQVVRNLEVTGQTDLAKSVMALMRDADKSTS 99
SSH11_36f           WSRPRSGVRPTNPAEAKISETKAKTLETWSQVVRNLEVTGQTDLAKSVMALMRDADKSTS 120
                    :.** . *****************************************************

SSH05_187b          SRTQELFDLAKSSHSKDKGIEQEL 95
SSH11_Cv21          SRTQELFDLAKSSHSKDKGIEQEL 123
SSH11_36f           SRTQELFDLAKSSHSKDKGIEQEL 144
                    ************************
```

GOE #45

return to main table

```
SSH13_56a           MYWRISKEKFAELDADNRIWWGKDGNNTPRLKRFLNEVKQGIVPQTLWTYSNVGHTQDAK 60
SSH22_187b          MYWRISKEKFAELDADNRIWWGKDGNNTPRLKRFLNEVKQGIVPQTLWTYSNVGHTQDAK 60
SSH31_36f           MYWRISKEKFAELDADNRIWWGKDGNNTPRLKRFLNEVKQGIVPQTLWTYSNVGHTQDAK 60
SSH07_Fb7           MYWRISKEKFAELDADNRIWWGKDGNNTPRLKRFLNEVKQGIVPQTLWTYSNVGHTQDAK 60
                    ************************************************************

SSH13_56a           KQLLDVLQFESSSDVFSTPKPV-------------------PARA-FERPPGQVQAGSSI 100
SSH22_187b          KQLLDVLQFESSSDVFSTPKPV-------------------PARASVVAAEVLLEALSKG 101
SSH31_36f           KQLLDVLQFESSSDVFSTPNSS-------------------GRPGRYYVADTVRHGLTLP 101
SSH07_Fb7           KQLLDVLQFESSSDVFSTPIAWSRPRYIRQHLRELEERLNFGVRQEVILAELKAKGYKTT 120
                    ******************* .                            .    .. .

SSH13_56a           CEDTR------------------------------------------------------- 105
SSH22_187b          LSQLRARPVTW------------------------------------------------- 112
SSH31_36f           LRSACC------------------------------------------------------ 107
SSH07_Fb7           LKGFRNLLYRARIRAAQNPEKFQTKVEQKNEKEDAQLLAKKSENKKEEVSKNPLKKKAGF 180
```

```
SSH13_56a           --------------
SSH22_187b          --------------
SSH31_36f           --------------
SSH07_Fb7           DFKGTNSFDESDLI 194
```

GOE #46 return to main table

```
SSH58_912c          MSQSIIPFDFHSHAVRVVMRDGNPWFVATDVMGALDYAATSNPARVTEHIPGEWIRVNPI 60
SSH38_Fb7           ------------------MIDGEAWFVAKDVMDALDYAETSNPARVTEHIPAEWVCVNPI 42
                                      * **:.****.***.***** ************.**: ****

SSH58_912c          HTLGGEQKLLCLAEPGLYLGRDHAIAWSRPRYIHFHADDYFHGEHSGGYP 110
SSH38_Fb7           HTNA---------------------------------------------- 46
                    ** .
```

GOE #47 return to main table

```
SSH43_187b          MKSIFALLAFMVFSTVASAQQNELVRVENLSTVESTSAATTLRPPMAAGIAWSRPRSIAI 60
SSH70_912c          ------------------------------------------------------------

SSH43_187b          SITSSNALASVGGLGGLTRAKTAAENIKTGLYALVGVIAMIYLIYLGVMAFTEKKSWADF 120
SSH70_912c          ---------------------------------------MIYLIYLGVMAFTEKKSWADF 21
                                                           *********************

SSH43_187b          GWGVVYVSLVGGAVALGGWAWTLFA 145
SSH70_912c          GWGVVYVSLVGGAVALGGWAWTLFA 46
                    *************************
```

GOE #48 return to main table

```
SSH20_36f           MNKTIDFSNAGALPTLDGRTEVFGEPTSKAKSKTNVGKFLVLGLVLMAFLFMVGGLLFYQ 60
SSH30_Fb7           MNKTIDFSNAGALPTLDGRTEVFGEPTSKAKSKTNVGKFLVLGLVLMAFLFMVGGLLFYQ 60
SSH31_56a           MNKTIDFSNAGALPTLDGRTEVFGEPTSKAKSKTNVGKFLVLGLVLMAFLFMVGGLLFYQ 60
SSH48_187b          -NKTIDFSNAGALPTLDGRTEVFGEPTSKAKSKTNVGKFLVLGLVLMAFLFMVGGLLFYQ 59
                     ***********************************************************

SSH20_36f           KYKTSAATTLFERPPGQVSQRNRNGIPWQSAVASSPQEPPQQQQLSNSFTKSTWKLTATH 120
SSH30_Fb7           KY---------------------------------------------------------- 62
SSH31_56a           KYKTSAATTLL-RAAARAGTR-------------SPR----------------------- 83
SSH48_187b          KYKTSAATTL-------------------------------------------------- 69
                    **

SSH20_36f           ALTSPTHVQVYYAMTATP 138
SSH30_Fb7           ------------------
SSH31_56a           -------------MIAT- 87
SSH48_187b          ------------------
```

GOE #49 return to main table

```
SSH07_187b          MKMYLRSLTLAFVLLPIMASAQTISEANVAKVKLKIVESEDRFTGLKTITAKSCIHLNQS 60
SSH25_187b          MKMYLRSLTLAFVLLPIMASAQTISEANVAKVKLKIVESEDRFTGLKTITAKSCIHLNQS 60
SSH11_56a           MKMYLRSLTLAFVLLPIMASAQTISEANVAKVKLKIVESEDRFTGLKTITAKSCIHLNQS 60
SSH12_Fb7           ------------------------------------------------------------

SSH07_187b          GMLTDLYLCPAIASKPNQPLVMYARGDYTGYGWAFLTGQAQFLIDGKQYSAQGTARPEEK 120
SSH25_187b          GMLTDLYLCPAIASKPNQPLVRYARGDYTGYGWAFLTGQAQFLIDGKQYSAQGTARPEEK 120
SSH11_56a           GMLTDLYLCPAIASKPNQPPGMYARGDYTGYGWAFLTGQAQFLIDGKQYSAQGTARPEEK 120
SSH12_Fb7           ------------------------------------------------------------

SSH07_187b          RVATCSGNVGCINKETARFSMTEELATAIANARDAEVRFVGRQGSVTGRLNAKHVAYFRE 180
SSH25_187b          RVATCSGNVGCINKETARFSMTEELATAIANARDAEVRFVGRQGSVTGRLNAKHVAYFRE 180
SSH11_56a           RVATCSGNVGCINKETARFSMTEELATAIANARDAEVRFVGRQGSVTGRLNAKHVAYFRE 180
SSH12_Fb7           --------------------MTEELATAIANARDAEVRFVGRQGSVTGRLNAKHVAYFRE 40
                                        ****************************************

SSH07_187b          MLRRYKTLGGVFESASTANEGTLEVNTSESENKVKN 216
SSH25_187b          MLRRYKTLGGVFESASTANEGTLEVNTSESENKVKN 216
```

```
SSH11_56a           MLRRYKTLGGVFESASTANEGTLEVNTSESENKVKN 216
SSH12_Fb7           MLRRYKTLGGVFESASTANEGTLEVNTSESEQ---- 72
                    *******************************:
```

GOE #50

[return to main table]

```
SSH46_187b          VPPQCSGDPVMCGQARIQWRIECSLRRDVKIKGGTCDAQPICTGEKCNAMEYASLLLQWR 60
SSH40_56a           -------------------------------------------------MEYASLLLQWR 11
SSH27_Fb7           -------------------------------------------------MEYASLLLQWR 11
SSH39_187b          ----------MCGQTRIQWRIECSLRRDVKIKGGTCDAQPICTGEKCNAMEYASLLLQWR 50
                                                                     ***********

SSH46_187b          TACALEKGSAGAVLGGDKNQGNADVGAIKDALTGKDGTVDTGEEGDPSGAFSDESGYGRE 120
SSH40_56a           TACALEKGSAGAVLGGDKNQGNADVGAIKDALTGKDGTVDTGEEGDPSGAFSDESGYGRE 71
SSH27_Fb7           TACALEKGSAGAVLGGDKNQGNADVGAIKDALTGKDGTVDTGEEGDPSGAFSDESGYGRE 71
SSH39_187b          TACALEKGSAGAVLGGDKNQGNADVGAIKDALTGKDGTVDTGEEGDPSGAFSDESGYGRE 110
                    ************************************************************

SSH46_187b          GYPKVKIDDKGRGYSRVCPKSIPINVFGNQIVISATPVCQFLQIGGKLVLIL-------- 172
SSH40_56a           GYPKVKIDDKGRGYSRVCPKSIPINVFGNQIVISATPVCQFLQIGGKLVLIL-------- 123
SSH27_Fb7           GYPKVKIDDKGRGYSRVCPKSIPTNVFGNQIVISATPVCQFLQIGGKLVLIL-------- 123
SSH39_187b          GYPKVKIDDKGRGYSRVCPKSIPINVFGNQIVISGTPVCQFLQIGGKLVLILDLDRDHAN 170
                    *********************** **********.*****************

SSH46_187b          ------------------------------------AALACLRIMGGRLEG--------- 187
SSH40_56a           ------------------------------------AALACLRIMGGRLEG--------- 138
SSH27_Fb7           ------------------------------------AALACLRIMGGRLEG--------- 138
SSH39_187b          LRGHGAGSMFERPLGQVPWSDFCILFRGNFQSRPLEKALQLLRIPSSGRPGRYCLLPAKM 230
                                                         **  *** ..   *

SSH46_187b          ----
SSH40_56a           ----
SSH27_Fb7           ----
SSH39_187b          ARSA 234
```

GOE #51

[return to main table]

```
SSH02_912c          VRGLIGQHTFIKRRFGTRWIDVWTWGELIDNVKSSSNRDLGEHVLRTLPSQVYGLYKSAE 60
SSH04_187b          ------------------------------------------------------------


SSH02_912c          VHTIKARIPFKVYLLALLMVLVVVCVVIAVRTLRPSSFSSRVLGTQAANGAERARPEAPA 120
SSH04_187b          --------------------------VVAA--------------------------EAPA 8
                                              *:*.                          ****

SSH02_912c          SHHPVGVENGDALRWETETAYAKDHLPRFASMPWTAPIYDSRSPTADPQLICMSGGEGLD 180
SSH04_187b          SHHPVGVENGDALRWETETAYAKDHLPRFASMPWTAPIYDSRSPTADPQLICMSGGEGLD 68
                    ************************************************************

SSH02_912c          AQGMYKGMSCTCYTEQGTLYEIPDGECRRIARRGPVYNPYRERTQELGGASQGQVTALPS 240
SSH04_187b          AQGMYKGMSCTCYTEQGTLYEIPDGECRRIARRGPVYNPYRERTQELGGASQGQVTALPS 128
                    ************************************************************

SSH02_912c          SRSTPGHVLVTSGHALP 257
SSH04_187b          SRSTPGHVLVTSGHALP 145
                    *****************
```

GOE #52

[return to main table]

```
SSH17_187b          ---------MREAVMQFCQEEFANRRYVAALHDDTDHTHVHVCVGTRDIDRADEPRLSPR 51
SSH03_Cv21          MPPGTPEKAMREAVMQFCQEEFANRRYVAALHDDTDHTHVHVCVGTRDIDRADEPRLSPR 60
SSH04_56a           ---------MREDVMQFCQEEFGNRRYVAGLHDDTDHAHVHVCVGTQDIDRADEPRLSPR 51
                             *** *********.******.*******:********:*************

SSH17_187b          KADLFRWRQGFADKLRENGIDAAASERRHRFNHRKPENPVVRQIRADNPKSAVYNERRAK 111
SSH03_Cv21          KADLFRWRQGFADKLRENGIDAAASERRHRFNHRKPENPVVRQIRADNPKSAVYNERRAK 120
SSH04_56a           KADLFRWRQGFADKLRENGIDAAASERRHRFNHRKPENPVVRQIRADNPKSAVYNERRAK 111
                    ************************************************************

SSH17_187b          EKALERAMKATARQEDAYVSPPLPPRDPCPGGRSIRAAARAGTFTANDR-VVRNNNIISN 170
SSH03_Cv21          EKALERAMKATARQEDAYVSPPLPPRVPRPRPR--------------------------- 153
SSH04_56a           EKALERAMKATARQEDAYVSPPLPPRVP-KVYEALKSDLQAALKTARAGPPLGTSPYPLD 170
```

```
                    **************************  *    .

SSH17_187b          A 171
SSH03_Cv21          -
SSH04_56a           A 171
```

GOE #53

return to main table

```
SSH10_Cv21          ------------------------------------------------------------
SSH32_912c          MSFVLGAKTELLVAAYSFTSKDIAFALTEAKARGIDVRVVVDHAQNTDDQGGYKAVDYLS 60


SSH10_Cv21          --------------MHHKFMVADGLHVQLGSFNYTSSANLRNAETAVAFRNAPELASLYR 46
SSH32_912c          SQGIPVFRCENYAAMHHHKFMVADGLHVQLGSFNYTSSANLRNAETAVAFRNAPDRAAAR- 119
                                  ***************************************: *:

SSH10_Cv21          TEWLRLSTEPKASVETVMAVDRGLAILKEFGI 78
SSH32_912c          ---AGLLQPPLP-------------------- 128
                         *   * .
```

GOE #54

return to main table

```
SSH27_36f           -MKSIFAL--------------LAFMVFSTVASAQQNELVRVENLS-TVESTTPQVPDIT 44
SSH63_912c          --MGIYEIEWPPA---------RSFMVFSTVASAQQNELVRVENLS-TVESTTPQVPDIT 48
SSH21_Cv21          LSVNLADLGRDHASPGRVAILISTFMVFSTVASAQQNELVRVENLAWSRPSTTPQVPDIT 60
                       .:  :               :*********************: :  **********

SSH27_36f           GNVINIANKKLRHVTVEFNLYDEQNNLVGNAIDVVTNLEPNGKWKFKATTTTPYQRFKLT 104
SSH63_912c          GNVINIANKKLRHVTVEFNLYDEQNNLVGNAIDVVTNLEPNGKWKFKATTTTPYQRFKLT 108
SSH21_Cv21          GNVINIANKKLRHVTVEFNLYDEQNNLVGNAIDVVTNLEPNGKWKFKATTTTPYQRFKLT 120
                    ************************************************************

SSH27_36f           NVEAY-----------LE---- 111
SSH63_912c          NVEDLGRDHAIVVRGAMEQRTD 130
SSH21_Cv21          NVEAY-----------LE---- 127
                    ***             :*
```

GOE #55

return to main table

```
SSH16_Cv21          MCALSVSEVSEVLGGNALTDSKSIGMDAGVFVGVMDGFTSPMFLGAFAGAERLIGAAWGG 60
SSH42_912c          MCALSVSEVSEVLGGNALTDSKSIGMDAGVFVGVMDGFTSPMFLGAFAGAERLIGAAWGG 60
                    ************************************************************

SSH16_Cv21          GWMTGTFIHKRFISMYPTIFYLGRDHANPAARRPGALAWSRPRYENDPRWTNATGAVVQL 120
SSH42_912c          GWMTGTFIHKRFISMYPTIFYLGRDHA---------IAWSRPR----------------- 94
                    ***************************         :******

SSH16_Cv21          EGLQECWPTKPISTATTDHDCLDRKGYKR 149
SSH42_912c          -----------------------------
```

GOE #56

return to main table

```
SSH23187b         MECTLFDKDQFVHIRINEKRDFETLPRKFFGSSFPKNVRPETILGGNNRPQHFDTIVITR
SSH39_36f          MECTLFDKDQFVHIRINEKRDFETLPRKFFGSSFPKNVRPETILGGNNRPQHFDTIVITR
SSH10_56a          MECTLFDKDQFVHIRINEKRDFETLPRKFFGSSFPKNVRPETILGGNNRPQHFDTIVITR
SSH08912c         MECTLFDKDQFVHIRINEKRDFETLPRKFFGSSFPKNVRPETILGGNNRPQHFDTIVITR
SSH24_Fb7          MECTLFDKDQFVHIRINEKRDFETLPRKFFGSSFPKNVRPETILGGNNRPQHFDTIVITR
SSH44_Cv21         MECTLFDKDQFVHIRINEKRDFETLPRKFFGSSFPKNVRPETILGGNNRPQHFDTIVITR
```

GOE #57

return to main table

```
SSH24_187b         MAAYPIIMLSIELVCSIFIKVQETFNNATLPDENVYKTAIEAALCLRIVSK
SSH22_36f          MAAYPIIMLSIELVCSIFIKVQETFNNATLPDENVYKTAIEAALCLRIVSK
SSH16_56a          MAAYPIIMLSIELVCSIFIKVQETFNNATLPDENVYKTAIEAALCLRIVSK
SSH22_Cv21         MAAYPIIMLSIELVCSIFIKVQETFNNATLPDENVYKTAIEAALCLRIVSK
SSH61_912c         MAAYPIIMLSIELVCSIFIKVQETFNNATLPDENVYKTAIEAALCLRIVSK
                   ***************************************************
```

GOE #58

return to main table

```
SSH22_36f          MAAYPIIMLSIELVCSIFIKVQETFNNATLPDENVYKTAIEAALCLRIVSK
SSH22_Cv21         MAAYPIIMLSIELVCSIFIKVQETFNNATLPDENVYKTAIEAALCLRIVSK
                    ***************************************************
```

GOE #59

[return to main table]

```
SSH08_36f       VRQEVILAELKAKGYKTTLKGFRNLLYRARIRAAQNPEKFQTKVEQKNEKEDAQLLAKKS
SSH15_56a       VRQEVILAELKAKGYKTTLKGFRNLLYRARIRAAQNPEKFQTKVEQKNEKEDAQLLAKKS
SSH45_Cv21      VRQEVILAELKAKGYKTTLKGFRNLLYRARIRAAQNPEKFQTKVEQKNEKEDAQLLAKKS
SSH69_912c      VRQEVILAELKAKGYKTTLKGFRNLLYRARIRAAQNPEKFQTKVEQKNEKEDAQLLAKKS
                ************************************************************

SSH08_36f       ENKKEEVSKNPLKKKAGFDFKGTNSFDESDLI
SSH15_56a       ENKKEEVSKNPLKKKAGFDFKGTNSFDESDLI
SSH45_Cv21      ENKKEEVSKNPLKKKAGFDFKGTNSFDESDLI
SSH69_912c      ENKKEEVSKNPLKKKAGFDFKGTNSFDESDLI
                ********************************
```

GOE #60

[return to main table]

```
SSH26_36f       MNKNQAAMHVEASKFIATNMQALQNQPNMADKSVEDLTKLAYWRGIVAEENKLQPKAVQD
SSH62_912c      MNKNQAAMHVEASKFIATNMQALQNQPNMADKSVEDLTKLAYWRGIVAEENKLQPKAVQD
SSH20_56a       MNKNQAAMHVEASKFIATNMQALQNQPNMADKSVEDLTKLAYWRGIVAEENKLQPKAVQD
                ************************************************************

SSH26_36f       EAIARFDKQAADPQFLKRLNQETEPKIHDRTTERVQQRDTHEQSL
SSH62_912c      EAIARFDKQAADPQFLKRLNQETEPKIHDRTTERVQQRDTHEQSL
SSH20_56a       EAIARFDKQAADPQFLKRLNQETEPKIHDRTTERVQQRDTHEQSL
                *********************************************
```

GOE #61

[return to main table]

```
SSH33_187b      MLLNTNNIAVNRPAAEAGLEMLEAGGDFADGVIAYDGTWLGAETFVSFDKKAVSLLTKHGHAARLL
SSH32_36f       MLLNTNNIAVNRPAAEAGLEMLEAGGDFADGVIAYDGTWLGAETFVSFDKKAVSLLTKHGHAARLL
                ******************************************************************
```

GOE #62

[return to main table]

```
SSH25_36f       VNKRQLIAAIVEKSGIKLTEDDPAFLLVDLNLMMLEKQTSEAAKQLEAATEKFNAVTTHN
SSH06_912c      VNKRQLIAAIVEKSGIKLTEDDPAFLLVDLNLMMLEKQTSEAAKQLEAATEKFNAVTTHN
                ************************************************************

SSH25_36f       VDDFVSVANEALS-KFMQRTNEIKSSLDDLSKKLAQVANVTSTSSAHEVTKISHRSELLW
SSH06_912c      VDDFVSVANEALS-KFMQRTNEIKSSLDDLSKKLAQVANVTSTSSAHEVTKISHRSELLW
                 ************************************************************

SSH25_36f       WLVPRPRPR
SSH06_912c      WLVPRPRPR
                 ********
```

GOE #63

[return to main table]

```
SSH05_36f       MSFTGILKKTRVPISAFVFIFAGTAYAQLQVYDAANISTSIQNHVESIAKWKQQFEQLKQ
SSH39_Cv21      MSFTGILKKTRVPISAFVFIFAGTAYAQLQVYDAANISTSIQNHVESIAKWKQQFEQLKQ
                ************************************************************

SSH05_36f       QIDQHQKQYEAITGSRGMGALLDNSALKASLPSDWKQVLSDVKKTSAYATERSKYPTSGN
SSH39_Cv21      QIDQHQKQYEAITGSRGMGALLDNSALKASLPSDWKQVLSDVKKTSAYATERSKYPTSGN
                ************************************************************

SSH05_36f       LQKTNAMYDVIASQDVIMSDLYSKANRRLNLIQSLTAQIDSANDPAAKADLANRLINEQN
SSH39_Cv21      LQKTNAMYDVIASQDVIMSDLYSKANRRLNLIQSLTAQIDSANDPAAKADLANRLINEQN
                ************************************************************

SSH05_36f       AIQANQNLVTILQAKQKQELEIASQAAVEELSCKEFKRSGC
SSH39_Cv21      AIQANQNLVTILQAKQKQELEIASQAAVEELSCKEFKRSGC
                *****************************************
```

GOE #64

[return to main table]

```
SSH07_187b      MSDSEMKIQANALMPELVAELVALKQEAERFLSTQKIHTKVIYDVFDGAELLTPETLARV
SSH29_Cv21      MSDSEMKIQANALMPELVAELVALKQEAERFLSTQKIHTKVIYDVFDGAELLTPETLARV
                ************************************************************

SSH07_187b      NNAVMIAHQMTANVQKRINALRGKLPEGYKDADGHFWNTNTFNLLRDTHKDFDQAVKIAK
```

```
SSH29_Cv21          NNAVMIAHQMTANVQKRINALRGKLPEGYKDADGHFWNTNTFNLLRDTHKDFDQAVKIAK
                    ************************************************************

SSH07_187b          KISEWASVKIDGTSYSK
SSH29_Cv21          KISEWASVKIDGTSYSK
                    *****************
```

GOE #65 [return to main table]

```
SSH03_187b          VVAAEVLSDVKKTSAYATERSKYPTSGNLQKTNAMYDVIASQDVIMSDLYSKANRRLNLI
SSH26_56a           VVAAEVLSDVKKTSAYATERSKYPTSGNLQKTNAMYDVIASQDVIMSDLYSKANRRLNLI
                    ************************************************************

SSH03_187b          QSLTAQIDSANDPAAKADLANRLINEQNAIQANQNLVTILQAKQKQELEIASQAAVEELS
SSH26_56a           QSLTAQIDSANDPAAKADLANRLINEQNAIQANQNLVTILQAKQKQELEIASQAAVEELS
                    ************************************************************

SSH03_187b          CKEFKRSGC
SSH26_56a           CKEFKRSGC
                    *********
```

GOE #66 [return to main table]

```
SSH13_36f           VWTRIDNPNGVFANIDSPATDQMGYSGIPGYVDTHFWQRFGSAIMLSLIKDFSQAYSQRV
SSH19_912c          VWTRIDNPNGVFANIDSPATDQMGYSGIPGYVDTHFWQRFGSAIMLSLIKDFSQAYSQRV
                    ************************************************************

SSH13_36f        ANRSNTGTTVIQPYANTTQATQDMGAEALRNSINIPPTLVVLPATAVNVMVARDVSFENV
912c_19          ANRSNTGTTVIQPYANTTQATQDMGAEALRNSINIPPTLVVLPATAVNVMVARDVSFENV
                 ************************************************************

SSH13_36f           FNLVE
SSH19_912c          FNLVE
                    *****
```

GOE #67 [return to main table]

```
SSH09_187b          MTELVAIVNDLASRGVTFESLTEHIDTSSASGKFAFHLFSALAEFERNTIKERTRAGLAA
SSH67_912c          MTELVAIVNDLASRGVTFESLTEHIDTSSASGKFAFHLFSALAEFERNTIKERTRAGLAA
                    ************************************************************

SSH09_187b          ARARGRMGGRPAKVTPKAKREMKALYTSQEVSVKDICTRYNITRSTFYRVVLERDYTANG
SSH67_912c          ARARGRMGGRPAKVTPKAKREMKALYTSQEVSVKDICTRYNITRSTFYRVVLERDYTANG
                    ************************************************************

SSH09_187b          KA
SSH67_912c          KA
                    **
```

GOE #68 [return to main table]

```
SSH34_912c          MKHIFNNFLDKYHNVILAIAISITSSNALASVGGLGGLTRAKTAAENIKTGLYALVGVIA
SSH19_Cv21          MKHIFNNFLDKYHNVILAIAISITSSNALASVGGLGGLTRAKTAAENIKTGLYALVGVIA
                    ************************************************************

SSH34_912c          MIYLIYLGVMAFTEKKSWADFGWGVVYVSLVGGAVALGGWAWTLFA
SSH19_Cv21          MIYLIYLGVMAFTEKKSWADFGWGVVYVSLVGGAVALGGWAWTLFA
                    **********************************************
```

GOE #69 [return to main table]

```
SSH41_912c          MKMRELSKVEIEQVSGASTSTTIWDDVVAATNGIWSGLVTATSNIFGGAVTATNNILGGT
SSH25_Cv21          MKMRELSKVEIEQVSGASTSTTIWDDVVAATNGIWSGLVTATSNIFGGAVTATNNILGGT
                    ************************************************************

SSH41_912c          AAGTNNIFNQFINSYFFRNYISISSLFSNDSTVVDKKS
SSH25_Cv21          AAGTNNIFNQFINSYFFRNYISISSLFSNDSTVVDKKS
                    **************************************
```

GOE #70 [return to main table]

```
SSH02_36f           MAILVHQRNEIFSKLKYFETSGDEAALERIQSELRSIDREISLISDTQNEDKYSIYNGNK
SSH24_Cv21          MAILVHQRNEIFSKLKYFETSGDEAALERIQSELRSIDREISLISDTQNEDKYSIYNGNK
```

```
                   ************************************************************

SSH02_36f          ESQQSKSEEITYKRWDIFKNFGLKGN
SSH24_Cv21         ESQQSKSEEITYKRWDIFKNFGLKGN
                   **************************
```

GOE #71 return to main table

```
SSH27_912c         MIPVIQPGEVVLVDTGIQSFDGDGIYLINIGHGQQIKALQDRGDAVYVVSANPLYQPVPF
SSH01_Cv21         MIPVIQPGEVVLVDTGIQSFDGDGIYLINIGHGQQIKALQDRGDAVYVVSANPLYQPVPF
                   ************************************************************

SSH27_912c         PSEGFIGGKIYIKNKIERFN
SSH01_Cv21         PSEGFIGGKIYIKNKIERFN
                   ********************
```

GOE #72 return to main table

```
SSH47_187b         VFFKVLPDGTEALLNSNIDPEHKQTVVLHEVIRTVRARLGDQVIEIINRAYKLPKFNETG
SSH77_912c         VFFKVLPDGTEALLNSNIDPEHKQTVVLHEVIRTVRARLGDQVIEIINRAYKLPKFNETG
                   ************************************************************

SSH47_187b         TSVPGAVRADRDI
SSH77_912c         TSVPGAVRADRDI
                   *************
```

GOE #73 return to main table

SSH34_56a
MNKKKIAIAVLVLLLLAMVSVLVMLYMSSGLLVKYLNLGDLTPHLSLPLDLAKFGTKKEKGFGIIAMVVSVGLPVMLFGIIGYAALAPKKRE
LHGSARFATRRELVKSGLLQSDKPDDQYPSILVGKQDKDFLFFRGQQFMFLAAPTRSGKGVGIVIPNLLHYRDSVVVLDIKGENFEITSGFR
AKCGQEVHKFAPDDENFQTACWNPLAYVRNDPRFRISDLMSITNILYPPSDDVWASTAERPVSWSGALHHGDV

GOE #74 return to main table

SSH03_912C
VSHEGVLILQTTEADTHFLDRRPRPRPRYSVVAAEVWNDKALLQNWREAWEVHTNQALEQAGHSVRIDHRTLAEQGIERIPQIHIGPKVLEM
ERRGIKTEIGGQALAIEHKNKAITALQSDLETVRNERNHEDLPGRPLNSVVAAEVFAQWFMCNPVRSCHYFST

GOE #75 return to main table

SSH06_912C
VNKRQLIAAIVEKSGIKLTEDDPAFLLVDLNLMMLEKQTSEAAKQLEAATEKFNAVTTHNVDDFVSVANEALSKFMQRTNEIKSSLDDLSKK
LAQVANVTSTSSAHEVTKISHRSELLWWLVPRPRPR

GOE #76 return to main table

SSH09_912C
MYQNYVARSQVTAGLAEITPGKVQAEILFSDAGTKTAITTPETIGLRTATTRCSSIAVNLTPSAGTGTIVCTITGNSQVNGQTITWTRSADN
TSGQGGTNNGGLWSCTTTVATTLSPSTCTSTAKNG

GOE #77 return to main table

SSH011_912C
MIVMKRLQAFKFQLRPNGQQERDMRRFAGACRFVFNRALALQNENHEARNKYIPYTQMASWLIAWKSAPETQWLKESPSQPLQQSLKDLERG
YRNFFQKRDLGRDHATEHVSR

GOE #78 return to main table

SSH14_912C
LSNEYSPDCAAAKECHQLTVGDYEDWHVPTLEEALSVIGADEFWYWGESGHWIWTCTPDLPGFERPPGQVLRSLIWLLLWKISSKRMLYPAM
YADAFSMMAMRPMAGNSSSSNRHWYLKTSAATTLQLVHVQYKKLLKKKIQKLTELVNIGRLHHHIDAHALFAHVAQIEDLPGRPLIRAAARA
GLALLVWSEFRG

GOE #79 return to main table

SSH15_912C
MHFIKNYDARQITKMISENGIKITLKEIKSILQNKEKKSQ

GOE #80 return to main table

SSH16_912C
LKPELASNNASLESDLIEKKKAEIKKKQEEEQAQIKAIEEDLPGHAPGRMAALASARPRSVDLTDMQS

GOE #81 [return to main table]
SSH21_912C
MLHFNAVGRTPFVERYKCALHDAIRAAARAGTRITQKRRKVRQYSQFNDALEGFPCEKYLGRDHAIICALLLMGACTKEKVPSTSEYLGRTT
I

GOE #82 [return to main table]
SSH26_912C
MPISLLTGIPGGGKTALMMERLVEESKRAERPLFAAGIDGLQPGLATVLEDPRQWNAKDAEGNYLVPNGAMIFIDEAWKWFGHLHDASRQPT
PHYVLETCPGGRSKPGQVFLQCRVCLVENGIYYQTKRF

GOE #83 [return to main table]
SSH30_912C
MDATEKQVANFAKVRTKIIEPAVKELTEKDGWIIQWVPVKAGRKVKALRFTFMRDPQGSLQF

GOE #84 [return to main table]
SSH31_912C
MATKEERLKALELKQAQIKAQIQAIKARDTAQERKDDTRRKVLIGSVVLKTSAATTL

GOE #85 [return to main table]
SSH33_912C
MTTSRALRLGENPTSNVAPVGNACSSIAPAKSRVGEGGDLGRDHANAPGRMAAGLAWSRPRSPSTSTAKDIAVKERVDRMAILVHQRNEIFS
KLKYFETSGDEAALERIQSELRSIDREISLISDTQNEDKYSIYNGNKESQQSKSEEIT

GOE #86 [return to main table]
SSH36_912C
LDLDISLRRQWTVDRERNFYLYEGGLTGNPAYEGVVYYCFYLYLNGTKFIVELDDEGGGSLVFEDNPYVVVWNKLVSIKVMPHDQVSSLKRV
PPAAWDTPDAPQPLLQNYSLNQFISIFKEAVTVNGAGNSNRNIHHPIVVRFGF

GOE #87 [return to main table]
SSH39_912C
VIGDFMLTQEQIDEICEMLGGPFSFLWGYIENAYATLPQQPDPASFEERKKDFLFLIGKLLDEGRLKLAKKGKFMTGTTEEQVEMFRKSFPA
SDEGMLLGAWFFADERPAGAVWVFKGEGENGEDYYEWT

GOE #88 [return to main table]
SSH40_912C
MRSLPVATTMPHDASMEARIVQLETIIPTLATKADVESLRADLPRPRPRYRAAARAGK

GOE #89 [return to main table]
SSH53_912C
LKVFVIRRELVNLLTALCTEAGSNLEVLALDIQHDHRVTVVEQVGNHDANALTRACRCGQEHELLATEEQKILVLFSYKNGWVLIIRFVRL

GOE #90 [return to main table]
SSH65_912C
MYLPGRPLEPGQVPAFGRIGVDALVEVERCLAVFLGIAK

ORF91 [return to main table]
SSH71_912C
VYWLWRRENITRRLKLQDIKQLFFCILCVSHIAVSPKRLRNNALLDFIKKTFEPWRVITILSPPDSSGRPGRSQQIFLLISANTSQVAGLKL
PDLKDR*

GOE #92 [return to main table]
SSH72_912C
MTQHGNKLASKDTAQRGVDMWNEFFGKSASVADITITRQEDFIKWLAEHGYTEGYCRRILGIGKSALNRSWKRGEITQVPFVELPRIGEPYP
HYASREQIVCLLNTDMPEHIWAYFLIRLCTACRGDAARGLQRFQIDTDASVVAAEVPRLYAMV

GOE #93 [return to main table]
SSH73_912C
VEARPHSIIEADIIIKEGAKIDEFSAISTGAVIGKKAYIGRNVSIGKNVSIGEATQIYNSVEIGEDTTIGSNVVIYINCKIGKGVIISDNVR
IGTDVEVYPHSSIANNIIVPDNAIVSGECPRPRPRYSSGRPGRSMH

GOE #94 return to main table
SSH75_912C
MSTKEQAPVLNKLARLPQTFVEAAVDFEKSKIEEIKRSRKIAWIIASVATVICSVSILAFLVALLTRSEPEPTILQVDKSTCPGGRSNCVPC
VTPKTTTMKSSTSIGSSGRPGRYLRRLRLVHD

GOE #95 return to main table
SSH01_Fb7
VTAYKKKPSEVGTWLKDEDQEITRGSVKLLREFLEEKMDQKETIDAEPEGNDAQQWVDNDSDANGKVVKAAKEVDPNKFERPPGQVLRTPCS
ISSDEASQ

GOE #96 return to main table
SSH02_Fb7
MKHRVLVMNGQKIVQNEQTPGKWHTEHVDKAGLLKPGIYNIYAATVADKAKEHEGTIVHVDKQAIYQQIGKQFVKHECIDFYKIPEIGGVKR
IAYDQSTGRAQVDVASEKLAKKRSR

GOE #97 return to main table
SSH05_Fb7
MEAIAEALKVPLTILLEYTDLDRNNLDMIDSRTQGLPPGYERVFAVLPEHQAFIVKKWSKAAQKKLAGD

GOE #98 return to main table
SSH06_Fb7
MQDEENHGSVSARAIEKLRRDLGPVVMGALNDPETGEVLLKTFF

GOE #99 return to main table
SSH08_Fb7
MDAISVVAAEVSSSGRPGRYLGLKPDNITKAGTVIYRVGSTAIRDDGDLLNVSHGAGDYGVEAALRMAMYLGRDHATVKGSDEFKKRVVQIA
AAARLNISFDDEVLDKRRKQLVSDAAVIVKLTDAMLSDKTAQFTQAQASRVGAFQEDALSEYDAAISRSINLANQEQINEQQRTRAKHYFVQ
RWSDRGGPNSRRDGHATRIRSGGNCQHDQSDILYDCL

GOE #100 return to main table
SSH11_Fb7
MRMQLDNTALAVMAGLKSVVAAEVPPNEMETVQVLGRVIDRGGSGGL

GOE #101 return to main table
SSH14_Fb7
MSKHEDFKGTKASMITLTKQEEKLLEAATAI

GOE #102 return to main table
SSH15_Fb7
MDYYATEQPLPNDWKELYILTSAITAVEKAAVRKIIERFFTIAEDGLHHNERVDKEIAKAQRRMVASRENGKKGGCPKKHSKNPAGSENTVF
SEPKDNPAKTQQPPYSGVLRPRPR

GOE #103 return to main table
SSH17_Fb7
MPYSPATIANYFLQRASKEGRALTPMQVFKLVYIAHGWHLGFRKEPLIDEVVEAWRHGPVISSLYRKMKQYGSGGITELLPVNPFSWATASW
SNPVSKIDEKSAEILDSVWNGYGHFGGIQLSEMTHKEGSPWWQVWNGPKRKETGVINLTINDDLIQEFYEQKIKAHKHGELYEKYRSNPDLG
RDHASIHQYSHRDGTE

GOE #104 return to main table
SSH19_Fb7
MSISQVGLGLFRVWRGTSAATTLHAPGRLAAEISVVAAQVLRGRRHLFSTASSDDQHGADY

GOE #105 return to main table
SSH23_Fb7
MIKVETRKPGEGMSFKSLGGPFAVSSVPVTDPAVLGNAYRKKRLLLASIRKLAKDLNLVKSKFEDLDYEECGIEMYLERRYVARCIQEAHEY
MARFLGDIEGGVNV

GOE #106 return to main table
SSH31_Fb7
MSFTGILKKTRVPISAFVFIFAGTAYAQLQVYDAANISTSIQNHVESIAKWKQQFEQLKQQIDQHQKQYEAITGSRGMGALLDNSALKASLP
SDWKQVPARASVVAAEVLSAG

GOE #107 return to main table
SSH32_Fb7
MGYEIQGILDHRRVHELAKVNGDLGRLGGLLKLWLTNDARVVDFTPATIRNLLRKIEVTQDEIRAVMTQVVCK

GOE #108 return to main table
SSH33_Fb7
VVAAEVLDAALEVQATQALNTRSEADKTYHLLISFREGENPAPEILEVIESRVCAALGYADYQRVSVVHHDTDNLHIHVAINKIHPKRYLPG
PHPLHELPAVWPRE

GOE #109 return to main table
SSH37_Fb7
MDYFKMMAIQSKTAPEDFTLQTAIHALEDLLNEGKLTEKQIATLVGIGAIIYREGFKQFQAGMHTNMFLSKFKNQE

GOE #110 return to main table
SSH39_Fb7
MHESVQHCAHAPGRLAAGFRAAARAGTAAAPRHDMNKNQAAMHVEASKFIATNMQALQNQPNMADKSVEDLTKLAYWRGIVAEENKLQPKAV
QDEAIARFDKQAADPQFLKRLNQETEPKIHDRTTERVQQRDTHEQSL

GOE #111 return to main table
SSH25_36f
VNKRQLIAAIVEKSGIKLTEDDPAFLLVDLNLMMLEKQTSEAAKQLEAATEKFNAVTTHNVDDFVSVANEALSKFMQRTNEIKSSLDDLSKK
LAQVANVTSTSSAHEVTKISHRSELLWW
LVPRPRPR

GOE #112 return to main table
SSH32_36f
MLLNTNNIAVNRPAAEAGLEMLEAGGDFADGVIAYDGTWLGAETFVSFDKKAVSLLTKHGHAARLL

GOE #113 return to main table
SSH35_36f
LQRSKCLEKCPDVTGTKVGTREEKRREDIKTKNNKIPDGILSASLDSEAPDIGEEHQNQKKPPLSDRIPYLGRDHAIAWSRPRYPAYTQWCK
ESGVYALSKVRFLGELERCVPKFRNKRVARTVDGKRRELVVIQGIGLIDAGI

GOE #114 return to main table
SSH36_36f
MLNFRFMVDDGFRADSHVLANENGISDTGILSKDGIVKNLDKVPDGYGFAYGNRIINVTMTTYI

GOE #115 return to main table
SSH38_36f
VVFADFTGELVKVVAPDIGNVRVQILDFPLLFLPVCAELYFAA

GOE #116 return to main table
SSH11_187b
MGESRVNLDYPDVIRSVEYTEAFIRSLIGFVPQKGRLKPITGCGDSMIPVIQPGEVVLVDTGIQSFDGDGIYLINIGHGQQIKALQDRGDAV
YVVSANPLYQPVPFPSEGFIGGKIYIKNKIERFN

GOE #117 return to main table
SSH18_187b
MWINLNISADTDVIRNNDAFADLAININNHITADGRVFSYFDAVVDLCRFTNRDVFANGDVAPNIGFFTDHCACADRGKFINLGSFFNDDVR
LND

GOE #118 return to main table
SSH22_187b
MYWRISKEKFAELDADNRIWWGKDGNNTPRLKRFLNEVKQGIVPQTLWTYSNVGHTQDAKKQLLDVLQFESSSDVFSTPKPVPARASVVAAE
VLLEALSKGLSQLRARPVTW

GOE #119 return to main table
SSH32_187b
VQFERVRVADRSGEVLYDSDIQSNKQPNALEALAASRKLTDIKLAEITKAWSAVEAVARQRNASFAYLEAVSGHVQRLDDMQKERIHGHAMN
KLDENAATLVHDARYAQHTRSELAKAATLGPGGRSNAIAWSRPRYRTQCWWCCCFA

GOE #120
return to main table

SSH03_56a
MGDGYESDAPLEIIDYLEVKESWALLNLPRPYDRVKIIIGRGNSNAPLINDGDLVFVDTSINSFIGEDIYVFNWNGKALIKRLAVNLKTNTL
RIISANPDWVPARAAARTSVVAAEVPTSIVLNATRYTGKSDA

GOE #121
return to main table

SSH12_56a
MPYPASRYAVMDSISIKYLGRDHANPSASSVVAAEVFQKYSQGSPMDKLHKVGLSCSVVAAEVQEKR

GOE #122
return to main table

SSH21_56a
MALMRDADKSTSSRTQELFDLAKSSHSKDKGIEQEL

GOE #123
return to main table

SSH22_56a
MKKLNVTLKIMCNTKLQHTQKQASRISYKNQNAIPPMRKIVDLKIVFPQYKLMP

GOE #124
return to main table

SSH27_56a
METIGSRVRKEREAQNINRSDFAKKTGIGYSTIAELERGGMQTTTKLRLIADALGVSLRWLETGKGEKIEATTATKSIATEKISQHYIRVEH
LDAEAGMGESRVNLDYPDVIRSVEYTEAFIRSLIGFVPARTAARKLAAIRAGSMITWSRPRY

GOE #125
return to main table

SSH34_56a
MNKKKIAIAVLVLLLLAMVSVLVMLYMSSGLLVKYLNLGDLTPHLSLPLDLAKFGTKKEKGFGIIAMVVSVGLPVMLFGIIGYAALAPKKRE
LHGSARFATRRELVKSGLLQSDKPDDQYPSILVGKQDKDFLFFRGQQFMFLAAPTRSGKGVGIVIPNLLHYRDSVVVLDIKGENFEITSGFR
AKCGQEVHKFAPDDENFQTACWNPLAYVRNDPRFRISDLMSITNILYPPSDDVWASTAERPVSWSGALHHGDV

GOE #126
return to main table

SSH04_Cv21
MCASGVTRFNISKKRFLKVCIPIPSTAIQYEIVKVLDTFTTLEARRRQYQYYRDALLRFGEGRDAATRVRWVTLGEIGSFIRGVGIQKSDFI
EFGAGCIHYGQIHTHYGTWADKTKSFIRSDFAARLRKANTGDLVMALRAKMMMHSVVVAEVC

GOE #127
return to main table

SSH05_Cv21
MVAVSLQSLCELVWVLRSRYGAARTDIAAALRMLLNTNNIAVNRPAAEAGLEMLEAGGDFADGVIAYDGTWLGAETFVSFDKKAVSLLTKHG
HAARLL

GOE #128
return to main table

SSH07_Cv21
MVDYKPLTDNEVRDTAVAYYTERQGKSLAKENPIIVLVGGQPGAGKSTASSMVKAETCPGGRSNSVVAAEVCVVMKHGITSWP

GOE #129
return to main table

SSH12_Cv21
LALIRSILRRAFDIWLWIDRCPHISLFPEPTKRVRWLTPVQARSLLSELPLHQRAIVIFALATGLRQANVLKLRWGQVDLVRKVLRIPADQA
KGRQAIRIPLSFHALQVLQAQRGQHHEWVFTYCGRPIRWVNTRAWHQALQRAGIQDFRWHDLRHTWASWHAQAGTPLYVLQDLGGWQSESMV
RRYAHLTPSHYSAYAEAVTEFLP

GOE #130
return to main table

SSH20_Cv21
MKKAIGGAVAMTILLAGCGRKTDANEKNFGTAVGQYLDKKGDLCLNITKWPVDVNDMDLRLQKSMPTGTAGRMAALEAVGLAKSEYTEVQGT
GFDGKPTGVKFKVKRYALTDAAKPFERQQGVERVDLNGRTKETQTDLCWGKKALDKVVKWEGPMKFGDYHGRGRGTCTRSTAWPIGRKSRKS
WQPSPMSDKSRKMLARKSSSTA

GOE #131
return to main table

SSH26_Cv21
MAGLRPVQIWVPDTRRPDFAEECRRQCLLVAQADSADTAIHQFMDEALADVDCWAE

GOE #132
return to main table

SSH27_Cv21

VVAAEVNKSAGELLADLNKSAGELRADFEKAQKENRTWMLATAIGLFAGILGVGGFVVGSIKGSYQALPAQSPPIIIQVPAQALQPPPQAAK
QP

GOE #133

return to main table

SSH33_Cv21
LNAAQRSLANSNRSLQQGQERTFIVWTRIDNPNGVFANIDSPATDQMGYSGIPGYVDTHFWQRFGSAIMLSLIKDFSQAYSQRVANRSNTGT
TVIQPYANTTQATQDMGAEALRNSINIPPTLVVLPATAVNVMVARDVSFENVFNLVE

GOE #134

return to main table

SSH36_Cv21
MQCVLPIVPINNHAPRPIFNDDVCQAHMHGTEFFFNAQQVQTGSTTGDKAEVLVNNLPTKR

GOE #135

return to main table

SSH45_Cv21
VRQEVILAELKAKGYKTTLKGFRNLLYRARIRAAQNPEKFQTKVEQKNEKEDAQLLAKKSENKKEEVSKNPLKKKAGFDFKGTNSFDESDLI
