# Supplementary material for: Comparative genomic characterization of citrus-associated Xylella fastidiosa strains
Source: BMC Genomics. 2007 Dec 21;8:474. doi: 10.1186/1471-2164-8-474 (PMC2262912; doi:10.1186/1471-2164-8-474)
Supplement: Additional File 3 — Most relevant BLAST hits found for the consensus sequences of each GOE. [file 1471-2164-8-474-S3.PDF]

Blast hit analysis of the 135 newly identified GOEs throughout the genomes of the *Xylella fastidiosa* strains used in this study

Update: 17 October 2007

Query id	subject ids	organism	description	% identity	% positives	alignment length	Mismat-ches	gap opens	query start..end	subject start..end	E-value	Bit score
**GOE #01** SSH33_56a_GOE_01 SSH40_Cv21_GOE_01 (276aa) SSH21_187b_GOE_01 SSH16_36f_GOE_01	ZP_00682678.1	Xylella fastidiosa Ann-1	Plasmid encoded RepA protein	99.12	99.56	227	2	0	29..255	52..278	2.00E-129	462
	YP_361657.1	Xanthomonas campestris pv. vesicatoria str. 85-10	putative replication protein A	75.69	84.40	218	52	1	39..255	102..319	3.00E-89	329
	NP_644784.1	Xanthomonas axonopodis pv. citri str. 306	replication protein A	75.69	84.40	218	52	1	39..255	63..280	3.00E-89	329
**GOE #02** SSH18_36f_GOE_02 SSH15_Cv21_GOE_02 (268aa) SSH02_56a_GOE_02 SSH16_187b_GOE_02 SSH28_Fb7_GOE_02 SSH57_912c_GOE_02 SSH22_912c_GOE_02	ZP_00682688.1	Xylella fastidiosa Ann-1	TrbL/VirB6 plasmid conjugal transfer protein	71.25	75.84	327	33	3	1..268	1..325	5.00E-100	365
	YP_980121.1	Verminephrobacter eiseniae EF01-2	TrbL/VirB6 plasmid conjugal transfer protein	77.12	89.83	236	54	0	1..236	1..236	2.00E-80	300
	ZP_01506051.1	Burkholderia phymatum STM815	TrbL/VirB6 plasmid conjugal transfer protein	30.36	50.00	224	145	2	18..241	26..238	1.00E-19	98.6
**GOE #03** SSH74_912c_GOE_03 SSH01_56a_GOE_03 (153aa) SSH23_912c_GOE_03	ZP_00682688.1	Xylella fastidiosa Ann-1	TrbL/VirB6 plasmid conjugal transfer protein	88.42	94.74	95	11	0	1..95	209..303	3.00E-37	156
	YP_980121.1	Verminephrobacter eiseniae EF01-2	TrbL/VirB6 plasmid conjugal transfer protein	65.98	77.32	97	33	0	1..97	211..307	6.00E-25	115
**GOE #04** SSH35_187b_GOE_04 (195aa) SSH25_Fb7_GOE_04	ZP_00682693.1	Xylella fastidiosa Ann-1	TRAG protein	97.84	99.46	185	4	0	1..185	1..185	4.00E-90	332
	YP_980116.1	Verminephrobacter eiseniae EF01-2	TRAG family protein	84.86	94.05	185	28	0	1..185	1..185	2.00E-81	303
	NP_660238.1	Haemophilus influenzae biotype aegyptius	TraK-like protein	43.81	62.37	194	96	6	1..184	5..195	8.00E-30	132
**GOE #05** SSH36_56a_GOE_05 (244aa) SSH15_36f_GOE_05 SSH12_187b_GOE_05 SSH50_912c_GOE_05	ZP_00682686.1	Xylella fastidiosa Ann-1	CagE. TrbE. VirB component of type IV transporter system	97.11	99.42	173	5	0	57..229	165..337	2.00E-97	356
	YP_980123.1	Verminephrobacter eiseniae EF01-2	CagE. TrbE. VirB component of type IV transporter system	87.86	95.95	173	21	0	57..229	142..314	6.00E-90	332
	ZP_01506049.1	Burkholderia phymatum STM815	CagE. TrbE. VirB component of type IV transporter system	35.68	51.54	227	124	7	24..229	99..324	2.00E-25	117
**GOE #06** SSH17_Cv21_GOE_06 (467aa) SSH36_187b_GOE_06 SSH37_56a_GOE_06	ZP_00682686.1	Xylella fastidiosa Ann-1	CagE. TrbE. VirB component of type IV transporter system	94.36	96.53	461	21	1	1..461	58..513	0.0	893
	YP_980123.1	Verminephrobacter eiseniae EF01-2	CagE. TrbE. VirB component of type IV transporter system	83.08	90.24	461	73	1	1..461	35..490	0.0	789
	ZP_00682673.1	Xylella fastidiosa Ann-1	probable conjugal transfer protein TraL	98.25	99.12	114	2	0	109..222	101..214	2.00E-57	223

**GOE #07**<br>SSH07_56a_GOE_07<br>SSH23_36f_GOE_07<br>SSH30_187b_GOE_07<br>SSH20_Fb7_GOE_07<br>SSH04_912c_GOE_07<br>(232aa)	YP_980149.1	Verminephrobacter eiseniae EF01-2	transfer origin protein. TraL	85.09	92.98	114	17	0	109..222	101..214	5.00E-50	199
	ZP_00682686.1	Xylella fastidiosa Ann-1	CagE. TrbE. VirB component of type IV transporter system	88.76	93.26	89	10	0	1..89	389..477	8.00E-42	172
**GOE #08**<br>SSH01_187b_GOE_08<br>(233aa)<br>SSH31_Cv21_GOE_08<br>SSH10_36f_GOE_08	ZP_00683687.1	Xylella fastidiosa Ann-1	phage-related integrase	93.98	96.39	166	10	0	1..166	106..271	6.00E-84	311
	ZP_00683687.1	Xylella fastidiosa Ann-1	phage-related integrase	96.30	98.52	135	5	0	24..158	81..215	2.00E-67	257
**GOE #10**<br>SSH04_36f_GOE_10<br>SSH28_912c_GOE_10<br>(55aa)	ZP_00682686.1	Xylella fastidiosa Ann-1	CagE. TrbE. VirB component of type IV transporter system	94.55	96.36	55	3	0	1..55	773..827	9.00E-24	112
	YP_980123.1	Verminephrobacter eiseniae EF01-2	CagE. TrbE. VirB component of type IV transporter system	85.45	90.91	55	8	0	1..55	750..804	2.00E-21	103
	CAM06592.1	Bartonella schoenbuchensis	VirB4-homolog	30.00	65.00	40	28	0	3..42	747..786	2.0	34.7
**GOE #11**<br>SSH31_187b_GOE_11<br>SSH13_Fb7_GOE_11<br>(185aa)	NP_454711.1	Salmonella enterica subsp. enterica serovar Typhi str. CT18	putative IS element transposase	82.35	92.44	119	21	0	13..131	166..284	5.00E-48	192
	YP_001463237.1	Escherichia coli E24377A	transposase. IS605 orfB family	81.51	89.92	119	22	0	13..131	160..278	1.00E-45	184
	YP_310889.1	Shigella sonnei Ss046	putative virulence protein	81.51	89.92	119	22	0	13..131	140..258	1.00E-45	184
**GOE #12**<br>SSH15_912c_GOE_12<br>SSH23_Cv21_GOE_12<br>(82aa)	YP_980133.1	Verminephrobacter eiseniae EF01-2	hypothetical protein Veis_5036	61.25	82.50	80	31	0	2..81	4..83	7.00E-17	89.4
**GOE #13**<br>SSH08_56a_GOE_13<br>SSH05_912c_GOE_13<br>SSH46_Cv21_GOE_13<br>(132aa)<br>SSH24_36f_GOE_13	ZP_00682673.1	Xylella fastidiosa Ann-1	probable conjugal transfer protein TraL	99.24	100.00	132	1	0	1..132	119..250	3.00E-65	249
	YP_980149.1	Verminephrobacter eiseniae EF01-2	transfer origin protein. TraL	84.85	93.18	132	20	0	1..132	119..250	3.00E-56	219
	AAP82043.1	Stenotrophomonas maltophilia	putative transfer protein	52.67	73.28	131	61	1	1..130	119..249	2.00E-32	140
**GOE #14**<br>SSH14_56a_GOE_14<br>(265aa)<br>SSH34_Cv21_GOE_14	ZP_00682691.1	Xylella fastidiosa Ann-1	conjugation TrbI-like protein	89.85	90.36	197	12	2	1..196	78..267	4.00E-65	249
	YP_980118.1	Verminephrobacter eiseniae EF01-2	conjugation TrbI family protein	63.55	69.63	214	54	4	1..196	78..285	2.00E-33	144
	ZP_00651851.1	Xylella fastidiosa Dixon	Phage/plasmid primase P4.C-terminal	79.03	85.48	62	13	0	204..265	782..843	1.00E-21	105
**GOE #15**<br>SSH41_56a_GOE_15<br>(113aa)<br>SSH26_912c_GOE_15	NP_779130.1	Xylella fastidiosa Temecula1	hypothetical protein PD0914	69.03	85.84	113	35	0	1..113	1..113	1.00E-37	158
	YP_451171.1	Xanthomonas oryzae pv. oryzae MAFF 311018	hypothetical protein XOO_2142	47.71	66.97	109	57	0	1..109	1..109	1.00E-19	98.6
	YP_364185.1	Xanthomonas campestris pv. vesicatoria str. 85-10	hypothetical protein XCV2454	47.71	66.97	109	57	0	1..109	1..109	1.00E-19	98.6
**GOE #16**<br>SSH28_Cv21_GOE_16	YP_980134.1	Verminephrobacter eiseniae EF01-2	Resolvase. N-terminal domain	91.24	94.16	137	12	0	45..181	65..201	5.00E-60	232

(181aa)<br>SSH19_56a_GOE_16	ZP_00682679.1	Xylella fastidiosa Ann-1	Resolvase. N-terminal:Resolvase helix-turn-helix region	97.81	97.81	137	3	0	45..181	64..200	5.00E-54	212
	YP_973981.1	Polaromonas naphthalenivorans CJ2	Resolvase. N-terminal domain	60.32	73.02	126	50	0	45..170	57..182	8.00E-34	145
**GOE #17**<br>SSH18_56a_GOE_17<br>SSH14_Fb7_GOE_17<br>SSH34_Fb7_GOE_17<br>SSH28_187b_GOE_17<br>(88aa)	ZP_00684052.1	Xylella fastidiosa Ann-1	Transcriptional regulator AbrB	100.00	100.00	88	0	0	1..88	1..88	1.00E-35	151
	YP_243669.1	Xanthomonas campestris pv. campestris str. 8004	hypothetical protein XC_2600	63.29	78.48	79	28	1	1..79	48..125	2.00E-19	97.4
	NP_637001.1	Xanthomonas campestris pv. campestris str. ATCC 33913	hypothetical protein XCC1631	63.29	78.48	79	28	1	1..79	17..94	2.00E-18	94.4
**GOE #18**<br>SSH20_912c_GOE_18<br>SSH09_36f_GOE_18<br>(210aa)<br>SSH35_Cv21_GOE_18	ZP_00682693.1	Xylella fastidiosa Ann-1	TRAG protein	100.00	100.00	155	0	0	17..171	424..578	3.00E-86	319
	YP_980116.1	Verminephrobacter eiseniae EF01-2	TRAG family protein	90.97	94.19	155	14	0	17..171	424..578	2.00E-78	293
	YP_558364.1	Burkholderia xenovorans LB400	Putative Type IV secretory pathway protein VirD4	48.18	67.88	137	65	3	17..150	322..455	9.00E-27	122
**GOE #19**<br>SSH19_56a_GOE_19<br>SSH20_187b_GOE_19<br>(136aa)	YP_001463097.1	Escherichia coli E24377A	transposase. IS605 family	81.48	91.67	108	20	0	28..135	30..137	8.00E-49	195
	NP_311304.1	Escherichia coli O157:H7 str. Sakai	putative transposase TnA	80.73	90.83	109	21	0	27..135	34..142	1.00E-48	194
	NP_313209.1	Escherichia coli O157:H7 str. Sakai	putative transposase	81.65	89.91	109	20	0	27..135	34..142	2.00E-48	194
**Query id**	**subject ids**	**organism**	**description**	**% identity**	**% positives**	**alignment length**	**Mismat-ches**	**gap opens**	**query start..end**	**subject start..end**	**E-value**	**Bit score**
**GOE #20**<br>SSH46_187b_GOE_20<br>(303aa)<br>SSH43_Cv21_GOE_20<br>SSH39_56a_GOE_20<br>SSH60_912c_GOE_20<br>SSH41_Fb7_GOE_20	ZP_00653045.1	Xylella fastidiosa Dixon	helix-turn-helix motif	98.82	98.82	85	1	0	1..85	1..85	2.00E-35	150
	ZP_00679904.1	Xylella fastidiosa Ann-1	helix-turn-helix motif	98.77	98.77	81	1	0	5..85	1..81	4.00E-34	146
**GOE #21**<br>SSH55_912c_GOE_21<br>SSH26_187b_GOE_21<br>(68aa)	No hit											
**GOE #22**<br>SSH28_36f_GOE_22<br>(107aa)<br>SSH52_912c_GOE_22<br>SSH06_187b_GOE_22	ZP_00652000.1	Xylella fastidiosa Dixon	Adenylate kinase. subfamily	95.74	95.74	47	2	0	1..47	119..165	3.00E-18	93.6
	Q9PGM3		Adenylate kinase (ATP-AMP transphosphorylase)	95.74	95.74	47	2	0	1..47	119..165	3.00E-18	93.6
**GOE #23**<br>SSH51_912c_GOE_23<br>SSH42_187b_GOE_23<br>(232aa)	gZP_00682694.1	Xylella fastidiosa Ann-1	hypothetical protein XfasoDRAFT_2670	98.28	98.71	232	4	0	1..232	137..368	2.00E-95	350
	YP_980115.1	Verminephrobacter eiseniae EF01-2	hypothetical protein Veis_5018	89.83	92.80	236	20	1	1..232	193..428	2.00E-90	333
	YP_067827.1	Aeromonas punctata	DNA primase TraC4	35.92	59.86	142	90	1	1..141	671..812	2.00E-17	91.3
**GOE #24**<br>SSH18_Fb7_GOE_24<br>SSH29_912c_GOE_24	ZP_00682687.1	Xylella fastidiosa Ann-1	plasmid-related exported protein	91.96	93.75	224	14	3	1..223	1..221	6.00E-88	325
	YP_980122.1	Verminephrobacter	type IV secretion	69.20	78.12	224	65	3	1.. 223	1..221	5.00E-60	232

(223aa)		eiseniae EF01-2	system family protein									
	YP_001257140.1	Brucella ovis ATCC 25840	P-type DNA transfer protein VirB5	30.84	48.90	227	129	6	16..222	9..227	2.00E-15	84.7
**GOE #25**<br>SSH41_Cv21_GOE_25<br>SSH18_912c_GOE_25<br>(117aa)	ZP_00682678.1	Xylella fastidiosa Ann-1	Plasmid encoded RepA protein	100.00	100.00	88	0	0	30..117	280..367	8.00E-45	181
	YP_361657.1	Xanthomonas campestris pv. vesicatoria str. 85-10	putative replication protein A	79.55	93.18	88	18	0	30..117	321..408	5.00E-35	149
	NP_644784.1	Xanthomonas axonopodis pv. citri str. 306	replication protein A	79.55	93.18	88	18	0	30..117	282..369	7.00E-35	149
**GOE #26**<br>SSH64_912c_GOE_26<br>SSH30_Cv21_GOE_26<br>SSH37_187b_GOE_26<br>(288aa)	ZP_00651189.1	Xylella fastidiosa Dixon	transferase hexapeptide repeat	75.00	82.74	168	40	1	16..181	5..172	6.00E-59	228
	YP_980124.1	Verminephrobacter eiseniae EF01-2	type IV secretory pathway. VirB3 family protein	84.15	92.68	82	13	0	207..288	46..127	1.00E-34	148
	ZP_00652852.1	Xylella fastidiosa Dixon	transferase hexapeptide repeat	46.43	60.12	168	88	1	16..181	5..172	1.00E-24	115
	NP_297776.1	Xylella fastidiosa 9a5c	UDP-3-O-3-hydroxymyristoyl glucosamine N-acyltransferase	47.31	60.48	167	85	2	16..179	11..177	1.00E-24	114
**GOE #27**<br>SSH47_Cv21_GOE_27<br>SSH12_912c_GOE_27<br>(108aa)	YP_980113.1	Verminephrobacter eiseniae EF01-2	Hypothetical protein Veis_5018	80.43	86.96	92	17	1	17..108	275..365	4.00E-29	129
**GOE #28**<br>SSH06_Cv21_GOE_28<br>SSH01_912c_GOE_28<br>(86aa)<br>SSH49_187b_GOE_28	No hit											
**GOE #29**<br>SSH54_912c_GOE_29<br>(87aa)<br>SSH33_36f_GOE_29	ZP_00651848.1	Xylella fastidiosa Dixon	Helix-turn-helix motif:Peptidase S24. S26A and S26B	76.09	89.13	46	11	0	42..87	211..256	1.00E-12	75.1
	ZP_00682615.1	Xylella fastidiosa Ann-1	Peptidase S24. S26A and S26B	76.09	84.78	46	11	0	42..87	207..252	1.00E-11	71.6
	NP_779330.1	Xylella fastidiosa Temecula1	hypothetical protein PD1124	76.09	84.78	46	11	0	42..87	207..252	1.00E-11	71.6
**GOE #30**<br>SSH19_187b_GOE_30<br>SSH24_56a_GOE_30<br>SSH14_Cv21_GOE_30<br>(236aa)	ZP_00682689.1	Xylella fastidiosa Ann-1	VirB8	97.03	97.46	236	7	0	1..236	1..236	3.00E-123	442
	YP_980120.1	Verminephrobacter eiseniae EF01-2	VirB8 family protein	85.33	91.56	225	33	0	12..236	14..238	5.00E-104	378
	ABI83644.1	Aeromonas veronii	conjugal transfer protein	38.67	56.89	225	134	3	14..236	24..246	5.00E-35	149
**GOE #31**<br>SSH18_Cv21_GOE_31<br>SSH35_912c_GOE_31<br>SSH05_56a_GOE_31<br>(289aa)	YP_980124.1	Verminephrobacter eiseniae EF01-2	type IV secretory pathway. VirB3 family protein	84.15	92.68	82	13	0	208..289	46..127	6.00E-35	149
	AAX12232.1	Xanthomonas axonopodis pv. glycines	probable conjugal transfer protein	40.91	58.44	154	85	1	8..161	48..195	3.00E-20	100
	ZP_01506048.1	Burkholderia phymatum STM815	type IV secretory pathway. VirB3-like	36.50	54.01	137	76	4	10..136	8..143	4.00E-14	80.1
**GOE #32**<br>SSH10_Fb7_GOE_32	ZP_00682692.1	Xylella fastidiosa Ann-1	type II secretion system protein E	96.67	98.89	90	3	0	1..90	244..333	1.00E-47	191

SSH35_56a_GOE_32<br>SSH37_36f_GOE_32<br>(274aa)<br>SSH29_187b_GOE_32<br>SSH47_912c_GOE_32<br>SSH08_Cv21_GOE_32	YP_980117.1	Verminephrobacter eiseniae EF01-2	type II secretion system protein E	95.56	98.89	90	4	0	1..90	244..333	5.00E-47	189
	ZP_00652971.1	Xylella fastidiosa Dixon	phage-related protein	68.75	78.12	128	40	0	101..228	15.. 142	1.00E-44	181
**GOE #33**<br>SSH66_912c_GOE_33<br>(136aa)<br>SSH03_36f_GOE_33	ZP_00683687.1	Xylella fastidiosa Ann-1	phage-related integrase	87.69	90.00	130	13	1	1..127	282..411	2.00E-56	211
	NP_297770.1	Xylella fastidiosa 9a5c	hypothetical protein XF0480	48.91	64.96	137	67	1	2..135	245..381	2.00E-28	119
	ZP_00651193.1	Xylella fastidiosa Dixon	phage-related integrase	51.16	67.44	129	60	1	2..127	245..373	3.00E-28	118
**GOE #34**<br>SSH72_912c_GOE_34<br>(136aa)<br>SSH03_36f_GOE_34	ZP_00683687.1	Xylella fastidiosa Ann-1	phage-related integrase	87.69	90.00	130	13	1	1..127	282..411	2.00E-56	211
	ZP_00651193.1	Xylella fastidiosa Dixon	phage-related integrase	51.16	67.44	129	60	1	2..127	245..373	3.00E-28	118
**GOE #35**<br>SSH38_187b_GOE_35<br>SSH06_56a_GOE_35<br>SSH49_912c_GOE_35<br>(317aa)<br>SSH16_Fb7_GOE_35<br>SSH07_36f_GOE_35	ZP_00682686.1	Xylella fastidiosa Ann-1	CagE. TrbE. VirB component of type IV transporter system	88.33	91.83	257	29	1	54..309	22.. 278	5.00E-133	474
	YP_980123.1	Verminephrobacter eiseniae EF	CagE. TrbE. VirB component of type IV transporter system	76.86	86.27	255	58	1	56..309	1..255	2.00E-117	423
	ZP_01986916.1	TraE Vibrio harveyi HY01	conjugal transfer protein	29.77	53.95	215	145	3	71..284	14..223	1.00E-24	115
**GOE #36**<br>SSH68_912c_GOE_36<br>(113aa)<br>SSH10_187b_GOE_36	ZP_00682678.1	Xylella fastidiosa Ann-1	Plasmid encoded RepA protein	73.49	77.11	83	15	2	1..83	1..76	2.00E-21	104
**GOE #37**<br>SSH27_187b_GOE_37<br>(95aa)	YP_380330.1	Geobacter metallireducens GS-15	CopG-like DNA-binding protein	54.10	85.25	61	28	0	4..64	2..62	4.00E-11	70.1
	NP_835378.1	Acidithiobacillus caldus	anti-toxin-like protein	64.41	77.97	59	21	0	4..62	2..60	7.00E-11	69.3
	AAG23807.1	Pseudomonas fluorescens	antidote	59.02	75.41	61	25	0	4..64	2..62	1.00E-10	68.2
**GOE #38**<br>SSH40_187b_GOE_38<br>SSH29_36f_GOE_38<br>(213aa)<br>SSH37_Cv21_GOE_38	ZP_00682692.1	Xylella fastidiosa Ann-1	type II secretion system protein E	96.30	99.07	108	4	0	1..108	119..226	4.00E-57	222
	YP_980117.1	Verminephrobacter eiseniae EF01-2	type II secretion system protein E	88.89	95.37	108	12	0	1..108	119..226	2.00E-51	204
	ZP_00651174.1	Xylella fastidiosa Dixon	N6 adenine-specific DNA methyltransferase. D12 class	98.57	98.57	70	1	0	123..192	25..94	3.00E-36	153
**GOE #39**<br>SSH26_Fb7_GOE_39<br>(88aa)<br>SSH43_912c_GOE_39	No hit											
**Query id**	**subject ids**	**organism**	**description**	**% identity**	**% positives**	**alignment length**	**Mismat-ches**	**gap opens**	**query start..end**	**subject start..end**	**E-value**	**Bit score**
**GOE #40**<br>SSH32_56a_GOE_40<br>SSH38_Cv21_GOE_40<br>SSH09_Fb7_GOE_40<br>SSH08_187b_GOE_40<br>(335aa)	ZP_00682686.1	Xylella fastidiosa Ann-1	CagE. TrbE. VirB component of type IV transporter system	90.14	95.07	284	27	1	27..309	502..785	8.00E-152	537
	YP_980123.1	Verminephrobacter eiseniae EF01-2	CagE. TrbE. VirB component of type IV transporter system	91.01	94.96	278	24	1	27..303	479..756	4.00E-150	531

SSH21_36f_GOE_40	BAD83765.1	Moraxella bovis Epp63	VirB4-like protein	52.13	67.73	282	128	3	27..303	483..762	4.00E-80	299
**GOE #41** SSH29_56a_GOE_41 SSH12_36f_GOE_41 (191aa) SSH32_Cv21_GOE_41 SSH24_912c_GOE_41	ZP_00682692.1	Xlella fastidiosa Ann-1	type II secretion system protein E	97.20	97.20	107	3	0	1..107	1..107	4.00E-54	213
	YP_980117.1	Verminephrobacter eiseniae EF01-2	type II secretion system protein E	86.92	95.33	107	14	0	1..107	1..107	2.00E-48	193
	CAM06593.1	Bartonella schoenbuchensis	VirB11-homolog	37.25	50.98	102	60	1	12..113	5..102	2.00E-09	64.7
**GOE #42** SSH25_56a_GOE_42 SSH13_Cv21_GOE_42 SSH76_912c_GOE_42 (159aa)	ZP_00682690.1	Xylella fastidiosa Ann-1	Conjugal transfer protein TrbG/VirB9/CagX	88.10	93.65	126	14	1	1..126	1..125	2.00E-56	220
	YP_980119.1	Verminephrobacter eiseniae EF01-2	Conjugal transfer protein TrbG/VirB9/CagX	82.54	88.10	126	21	1	1..126	1..125	1.00E-51	204
	BAD83760.1	Moraxella bovis Epp63	virB9-like protein	41.74	60.00	115	63	2	16..130	24..134	7.00E-18	92.4
**GOE #43** SSH13_912c_GOE_43 (84aa) SSH28_56a_GOE_43	ZP_00682701.1	Xylella fastidiosa Ann-1	SpoVT/AbrB-like	82.09	85.07	67	12	0	1..67	1..67	3.00E-20	100
	NP_644714.1	Xanthomonas axonopodis pv. citri str. 306	plasmid stable inheritance protein I	80.77	80.77	52	10	0	1..52	1..52	7.00E-15	82.4
	AAX12222.1	Xanthomonas axonopodis pv. glycines	probable plasmid stable inheritance protein I	73.08	82.69	52	14	0	1..52	1..52	1.00E-13	78.6
**GOE #44** SSH05_187b_GOE_44 SSH11_Cv21_GOE_44 SSH11_36f_GOE_44 (144aa)	ZP_00682695.1	Xylella fastidiosa Ann-1	hypothetical protein XfasoDRAFT_2671	92.50	96.25	80	6	0	65..144	255..334	5.00E-32	139
	YP_980114.1	Verminephrobacter eiseniae EF01-2	Relaxase/mobilization nuclease family protein	75.00	81.25	80	19	1	65..144	299..377	1.00E-22	108
	ZP_00682701.1	Xylella fastidiosa Ann-1	SpoVT/AbrB-like	78.67	81.33	75	14	2	1..75	1..73	5.00E-22	106
**GOE #45** SSH13_56a_GOE_45 SSH22_187b_GOE_45 SSH31_36f_GOE_45 SSH07_Fb7_GOE_45 (194aa)	ZP_00682672.1	Xylella fastidiosa Ann-1	hypothetical protein XfasoDRAFT_2648	98.15	99.07	108	2	0	87..194	11..118	9.00E-44	178
	NP_779052.1	Xylella fastidiosa Temecula1	methyltransferase. type III restriction-modification system	100.00	100.00	79	0	0	1..79	260..338	7.00E-42	172
	NP_635609.1	Xanthomonas campestris pv. campestris str. ATCC 33913	methyltransferase	76.92	87.18	78	18	0	2..79	261..338	1.00E-30	134
**GOE #46** SSH58_912c_GOE_46 (110aa) SSH38_Fb7_GOE_46	ZP_00681894.1	Xylella fastidiosa Ann-1	BRO. N-terminal	86.75	89.16	83	9	1	1..81	1..83	3.00E-35	150
	ZP_00652985.1	Xylella fastidiosa Dixon	BRO. N-terminal	84.34	86.75	83	11	1	1..81	1..83	3.00E-33	143
	ZP_00681895.1	Xylella fastidiosa Ann-1	BRO. N-terminal	58.23	65.82	79	28	2	1..78	1..75	1.00E-15	85.1
**GOE #47** SSH43_187b_GOE_47 (145aa) SSH70_912c_GOE_47	ZP_00682699.1	Xylella fastidiosa Ann-1	hypothetical protein XfasoDRAFT_2675	95.51	97.75	89	4	0	57..145	18..106	7.00E-32	139
	YP_980125.1	Verminephrobacter eiseniae EF01-2	hypothetical protein Veis_5028	88.73	95.77	71	8	0	75.. 145	32..102	2.00E-24	114
	ABI83638.1	Aeromonas veronii	conjugal transfer prepropilin	52.86	68.57	70	33	0	75..144	30..99	9.00E-10	65.5
**GOE #48** SSH20_36f_GOE_48 (138aa) SSH30_Fb7_GOE_48	ZP_00682691.1	Xylella fastidiosa Ann-1	conjugation TrbI-like protein	92.06	96.83	63	5	0	1..63	1..63	6.00E-24	112
	YP_980118.1	Verminephrobacter eiseniae EF01-2	conjugation TrbI family protein	72.06	83.82	68	19	0	1..68	1..68	4.00E-19	96.3

SSH31_56a_GOE_48<br>SSH48_187b_GOE_48	XP_001670867.1	Caenorhabditis briggsae	Hypothetical protein CBG03474	31.15	54.10	61	42	0	70..130	734..794	1.5	35.0
**GOE #49**<br>SSH07_187b_GOE_49<br>SSH25_187b_GOE_49 (216aa)<br>SSH11_56a_GOE_49<br>SSH12_Fb7_GOE_49	No hit											
**GOE #50**<br>SSH46_187b_GOE_50<br>SSH40_56a_GOE_50<br>SSH27_Fb7_GOE_50<br>SSH39_187b_GOE_50 (234aa)	NP_779129.1	Xylella fastidiosa Temecula1	hypothetical protein PD0913	58.02	75.31	162	66	1	1..162	301..460	4.00E-49	196
	ZP_00684138.1	Xylella fastidiosa Ann-1	phage-related protein	58.02	77.78	162	66	2	1..162	334..493	1.00E-45	184
	YP_364186.1	Xanthomonas campestris pv. vesicatoria str. 85-10	hypothetical protein XCV2455	52.47	69.14	162	73	2	1..162	297..454	7.00E-40	165
**GOE #51**<br>SSH02_912c_GOE_51 (257aa)<br>SSH04_187b_GOE_51	ZP_00684137.1	Xylella fastidiosa Ann-1	Zonular occludens toxin	59.46	70.66	259	103	2	1..257	126..384	2.00E-77	290
	NP_779131.1	Xylella fastidiosa Temecula1	hypothetical protein PD0915	63.09	74.68	233	85	1	1..232	137..369	3.00E-77	289
	YP_451170.1	Xanthomonas oryzae pv. oryzae MAFF 311018	hypothetical protein XOO_2141	53.78	68.00	225	97	2	1..225	123..340	1.00E-62	241
**GOE #52**<br>SSH17_187b_GOE_52 (171aa)<br>SSH03_Cv21_GOE_52<br>SSH04_56a_GOE_52	ZP_00682695.1	Xylella fastidiosa Ann-1	hypothetical protein XfasoDRAFT_2671	99.22	99.22	128	1	0	1..128	102..229	5.00E-61	235
	YP_980114.1	Verminephrobacter eiseniae EF01-2	Relaxase/mobilization nuclease family protein	88.49	92.09	139	16	0	1..139	146..284	8.00E-58	225
	NP_444535.1	Plasmid pIPO2T	TraR protein	37.42	51.53	163	88	3	1..163	146..294	5.00E-13	76.3
**GOE #53**<br>SSH10_Cv21_GOE_53 (128aa)<br>SSH32_912c_GOE_53	ZP_00682693.1	Xylella fastidiosa Ann-1	TRAG protein	100.00	100.00	78	0	0	1..78	681..758	7.00E-39	162
	YP_980116.1	Verminephrobacter eiseniae EF01-2	TRAG family protein	98.72	100.00	78	1	0	1..78	681..758	1.00E-38	161
	YP_256999.1	Sodalis glossinidius	hypothetical protein pSG1GA_70	47.37	60.53	76	36	2	1..73	117..191	1.00E-08	61.6
**GOE #54**<br>SSH27_36f_GOE_54<br>SSH63_912c_GOE_54 (130aa)<br>SSH21_Cv21_GOE_54	YP_826191.1	Solibacter usitatus Ellin6076	hypothetical protein Acid_4949	39.06	59.38	64	39	0	47..110	71..134	6.00E-06	52.8
	YP_001110588.1	Burkholderia vietnamiensis G4	hypothetical protein Bcep1808_6898	27.08	48.96	96	69	1	15..110	58..152	3.00E-04	47.4
**GOE #55**<br>SSH16_Cv21_GOE_55 (149)<br>SSH42_912c_GOE_55	ZP_00681382.1	Xylella fastidiosa Ann-1	hypothetical protein XfasoDRAFT_2812	62.50	67.50	80	30	0	1..80	1..80	8.00E-12	72.4
**GOE #56**<br>SSH23187b_GOE_56 (60aa)<br>SSH39_36f_GOE_56<br>SSH10_56a_GOE_56<br>SSH08912c_GOE_56<br>SSH24_Fb7_GOE_56<br>SSH44_Cv21_GOE_56	ZP_00683691.1	Xylella fastidiosa Ann-1	hypothetical protein XfasoDRAFT_1950	77.55	83.67	49	11	0	11..59	120..168	4.00E-15	83.6
**GOE #57**<br>SSH24_187b_GOE_57 (51aa)<br>SSH22_36f_GOE_57	No hit											

SSH16_56a_GOE_57<br>SSH22_Cv21_GOE_57<br>SSH61_912c_GOE_57												
**GOE #58**<br>SSH22_36f_GOE_58<br>(51aa)<br>SSH22_Cv21_GOE_58	No hit											
**GOE #59**<br>SSH08_36f_GOE_59<br>(92aa)<br>SSH15_56a_GOE_59<br>SSH45_Cv21_GOE_59<br>SSH69_912c_GOE_59	ZP_00682672.1	Xylella fastidiosa Ann-1	hypothetical protein XfasoDRAFT_2648	97.83	98.91	92	2	0	1..92	27..118	8.00E-30	132
	YP_980150.1	Verminephrobacter eiseniae EF01-2	hypothetical protein Veis_5053	63.04	76.09	92	34	0	1..92	26..117	5.00E-18	92.8
**Query id**	**subject ids**	**organism**	**description**	**% identity**	**% positives**	**alignment length**	**Mismat-ches**	**gap opens**	**query start..end**	**subject start..end**	**E-value**	**Bit score**
**GOE #60**<br>SSH26_36f_GOE_60<br>(105aa)<br>SSH62_912c_GOE_60<br>SSH20_56a_GOE_60	ZP_00682694.1	Xylella fastidiosa Ann-1	hypothetical protein XfasoDRAFT_2670	99.05	100.00	105	1	0	1..105	264..368	2.00E-38	160
	YP_980115.1	Verminephrobacter eiseniae EF01-2	hypothetical protein Veis_5018	91.43	94.29	105	9	0	1..105	324..428	1.00E-36	154
**GOE #61**<br>SSH33_187b_GOE_61<br>(66aa)<br>SSH32_36f_GOE_61	ZP_00684053.1	Xylella fastidiosa Ann-1	conserved hypothetical protein	98.48	100.00	66	1	0	1..66	66..131	2.00E-27	124
	AAW76134.1	Xanthomonas oryzae pv. oryzae KACC10331	conserved hypothetical protein	70.77	81.54	65	19	0	2..66	213..277	9.00E-20	98.6
	YP_988599.1	Bartonella bacilliformis KC583	hypothetical protein BARBAKC583_0275	69.23	84.62	65	20	0	2..66	67..131	1.00E-19	98.2
**GOE #62**<br>SSH25_36f_GOE_62<br>(128aa)<br>SSH06_912c_GOE_62	ZP_00682696.1	Xylella fastidiosa Ann-1	hypothetical protein XfasoDRAFT_2672	96.75	97.56	123	4	0	1..123	1..123	2.00E-53	211
	YP_980148.1	Verminephrobacter eiseniae EF01-2	hypothetical protein Veis_5051	74.80	84.55	123	31	0	1..123	1..123	3.00E-32	140
**GOE #63**<br>SSH05_36f_GOE_63<br>(221aa)<br>SSH39_Cv21_GOE_63	ZP_00682687.1	Xylella fastidiosa Ann-1	plasmid-related exported protein	95.02	96.83	221	11	0	1..221	1..221	6.00E-93	342
	YP_980122.1	Verminephrobacter eiseniae EF01-2	type IV secretion system family protein	71.49	81.00	221	63	0	1..221	1..221	2.00E-65	250
	NP_444522.1	Plasmid pIPO2T	TraF protein	34.74	53.52	213	114	4	20..207	15..227	7.00E-20	99.0
**GOE #64**<br>SSH07_187b_GOE_64<br>(137aa)<br>SSH29_Cv21_GOE_64	NP_644763.1	Xanthomonas axonopodis pv. citri str. 306	hypothetical protein XACb0035	75.74	88.97	136	32	1	1..135	1..136	1.00E-50	201
**GOE #65**<br>SSH03_187b_GOE_65<br>(129aa)<br>SSH26_56a_GOE_65	ZP_00682687.1	Xylella fastidiosa Ann-1	plasmid-related exported protein	96.80	98.40	125	4	0	5..129	97..221	7.00E-47	188
	YP_980122.1	Verminephrobacter eiseniae EF01-2	type IV secretion system family protein	75.20	84.00	125	31	0	5..129	97..221	4.00E-37	156
**GOE #66**<br>SSH13_36f_GOE_66<br>(125aa)<br>SSH19_912c_GOE_66	ZP_00682691.1	Xylella fastidiosa Ann-1	conjugation TrbI-like protein	96.80	98.40	125	4	0	1..125	308..432	1.00E-63	244
	YP_980118.1	Verminephrobacter eiseniae EF01-2	conjugation TrbI family protein	92.00	93.60	125	10	0	1..125	326..450	2.00E-60	234
	NP_444528.1	Plasmid pIPO2T	TraL protein	47.15	65.85	123	61	1	1..123	262..380	9.00E-26	118
**GOE #67**<br>SSH09_187b_GOE_67<br>(122aa)<br>SSH67_912c_GOE_67	YP_980134.1	Verminephrobacter eiseniae EF01-2	Resolvase. N-terminal domain	90.98	94.26	122	11	0	1..122	80..201	3.00E-50	200
	ZP_00682679.1	Xylella fastidiosa Ann-1	Resolvase. N-terminal:Resolvase	98.36	98.36	122	2	0	1..122	79..200	3.00E-44	179

			helix-turn-helix region									
	|YP_973981.1	Polaromonas naphthalenivorans CJ2	Resolvase. N-terminal domain	57.66	72.07	111	47	0	1..111	72..182	1.00E-26	121
**GOE #68** SSH34_912c_GOE_68 (106aa) SSH19_Cv21_GOE_68	ZP_00682699.1	Xylella fastidiosa Ann-1	hypothetical protein XfasoDRAFT_2675	92.45	95.28	106	8	0	1..106	1..106	8.00E-37	155
	ref|YP_980125.1	Verminephrobacter eiseniae EF01-2	hypothetical protein Veis_5028	88.73	95.77	71	8	0	36..106	32..102	2.00E-23	110
	ABI83638.1	Aeromonas veronii	conjugal transfer prepropilin	52.86	68.57	70	33	0	36..105	30..99	3.00E-09	63.9
**GOE #69** SSH41_912c_GOE_69 SSH25_Cv21_GOE_69 (101aa)	No hit											
**GOE #70** SSH02_36f_GOE_70 (86aa) SSH24_Cv21_GOE_70	ZP_00682697.1	Xylella fastidiosa Ann-1	hypothetical protein XfasoDRAFT_2673	91.86	95.35	86	7	0	1..86	74..159	6.00E-32	139
	YP_980126.1	Verminephrobacter eiseniae EF01-2	hypothetical protein Veis_5029	46.00	62.00	100	40	2	1..86	53..152	8.00E-13	75.9
**GOE #71** SSH27_912c_GOE_71 (80aa) SSH01_Cv21_GOE_71	ZP_00652969.1	Xylella fastidiosa Dixon	Helix-turn-helix motif:Peptidase S24. S26A and S26B	98.75	100.00	80	1	0	1..80	145..224	1.00E-39	164
	NP_297986.1	Xylella fastidiosa 9a5c	phage-related repressor protein	63.75	75.00	80	28	1	1..80	143..221	3.00E-22	107
	ZP_00653051.1	Xylella fastidiosa Dixon	Peptidase S24. S26A and S26B	49.37	63.29	79	39	1	1..78	179..257	6.00E-13	76.3
**GOE #72** SSH47_187b_GOE_72 (73aa) SSH77_912c_GOE_72	ZP_00682690.1	Xylella fastidiosa Ann-1	Conjugal transfer protein TrbG/VirB9/CagX	95.89	98.63	73	3	0	1..73	187..259	8.00E-33	142
	YP_980119.1	Verminephrobacter eiseniae EF01-2	Conjugal transfer protein TrbG/VirB9/CagX	91.55	97.18	71	6	0	1..71	191..261	3.00E-30	133
**GOE #73** SSH34_56a_GOE_73 (257aa)	ZP_00682693.1	Xylella fastidiosa Ann-1	TRAG protein	98.35	99.59	243	4	0	1..243	1..243	3.00E-130	465
	YP_980116.1	Verminephrobacter eiseniae EF01-2	TRAG family protein	87.65	95.06	243	30	0	1..243	1..243	2.00E-120	433
	NP_642936.1	Xanthomonas axonopodis pv. citri str. 306	VirD4 protein	51.74	63.18	201	83	5	45..242	49..238	2.00E-44	180
**GOE #74** SSH03_912C_GOE_74 (165aa)	AAK13432.1	Xylella fastidiosa	repB/MobA-like protein	95.00	96.00	100	5	0	37..136	155..254	6.00E-47	189
	NP_065281.1	uncultured eubacterium pIE1115	putative mobilization protein	47.90	59.66	119	62	0	14..132	137.. 255	3.00E-20	100
	NP_862661.1	uncultured bacterium	putative mobilization protein	47.06	58.82	119	63	0	14..132	137..255	3.00E-20	100
**GOE #75** SSH06_912C_GOE_75(128aa)	ZP_00682696.1	Xylella fastidiosa Ann-1	hypothetical protein XfasoDRAFT_2672	96.75	97.56	123	4	0	1..123	1..123	2.00E-53	211
	YP_980148.1	Verminephrobacter eiseniae EF01-2	hypothetical protein Veis_5051	74.80	84.55	123	31	0	1..123	1..123	3.00E-32	140
	YP_720015.1	Trichodesmium erythraeum IMS101	Rho termination factor-like	31.51	46.58	73	35	2	1..73	31..88	1.1	35.4
**GOE #76** SSH09_912C_GOE_76 (127aa)	NP_780107.1	Xylella fastidiosa Temecula1	fimbrial protein	60.16	69.92	123	44	2	1..123	29..146	1.00E-20	102
	ZP_00680431.1	Xylella fastidiosa Ann-1	Fimbrial protein pilin	61.54	74.36	117	38	3	1..117	29..138	1.00E-20	101

**GOE #77** SSH011_912C_GOE_77 (113aa)	ZP_01700173.1	Escherichia coli B	transposase. IS605 OrfB family	85.57	92.78	97	14	0	4..100	20..116	4.00E-41	169
	YP_001463237.1	Escherichia coli E24377A	transposase. IS605 orfB family	84.54	91.75	97	15	0	4..100	1..97	2.00E-40	167
	ZP_00682675.1	Xylella fastidiosa Ann-1	Transposase, IS605 OrfB	58.44	72.73	77	32	0	24.. 100	1.. 77	8,00E-18	92.4
**GOE #78** SSH14_912C_GOE_78 (196aa)	ZP_00684331.1	Xylella fastidiosa Ann-1	conserved hypothetical protein	77.97	86.44	59	13	0	1..59	52..110	7.00E-22	105
	ZP_00652856.1	Xylella fastidiosa Dixon	conserved hypothetical protein	65.52	72.41	58	19	1	2..59	48..104	2.00E-14	81.3
	NP_779334.1	Xylella fastidiosa Temecula1	hypothetical protein PD1128	56.14	71.93	57	25	0	2..58	48..104	3.00E-14	80.5
**GOE #79** SSH15_912C_GOE_79 (40aa)	No hit											
**Query id**	**subject ids**	**organism**	**description**	**% identity**	**% positives**	**alignment length**	**Mismat-ches**	**gap opens**	**query start..end**	**subject start..end**	**E-value**	**Bit score**
**GOE #80** SSH16_912C_GOE_80 (68aa)	No hit											
**GOE #81** SSH21_912C_GOE_81 (93aa)	No hit											
**GOE #82** SSH26_912C_GOE_82 (130aa)	YP_364184.1	Xanthomonas campestris pv. vesicatoria str. 85-10	filamentous phage Cf1c related protein	79.59	91.84	98	20	0	1..98	1..98	4.00E-42	172
	YP_451170.1	Xanthomonas oryzae pv. oryzae MAFF 311018	hypothetical protein XOO_2141	77.55	90.82	98	22	0	1..98	1..98	5.00E-41	169
	NP_779131.1	Xylella fastidiosa Temecula1	hypothetical protein PD0915	66.33	81.63	98	31	1	1..96	12..109	9.00E-33	142
**GOE #83** SSH30_912C_GOE_83 (62aa)	AAK13433.1	Xylella fastidiosa	replication initiator-like protein	90.32	95.16	62	6	0	1..62	206..267	3.00E-23	110
	YP_025704.1	Pseudomonas syringae pv. maculicola	replication protein	65.57	78.69	61	21	0	1..61	223..283	3.00E-14	80.5
	ABC41159.1	Neisseria lactamica	pNL18.2_p1	60.00	73.33	60	24	0	1..60	195..254	8.00E-13	75.9
**GOE #84** SSH31_912C_GOE_84 (57aa)	No hit											
**GOE #85** SSH33_912C_GOE_85 (150aa)	ZP_00682697.1	Xylella fastidiosa Ann-1	hypothetical protein XfasoDRAFT_2673	89.89	94.38	89	9	0	62..150	56..144	4.00E-34	146
	YP_980126.1	Verminephrobacter eiseniae EF01-2	hypothetical protein Veis_5029	41.67	61.46	96	42	2	69..150	42..137	7.00E-08	59.3
	XP_001246860.1	Coccidioides immitis RS	hypothetical protein CIMG_00631	40.43	53.19	47	19	1	40..77	18..64	0.65	36.2
**GOE #86** SSH36_912C_GOE_86 (145aa)	ZP_00682956.1	Xylella fastidiosa Ann-1	conserved hypothetical protein	94.48	96.55	145	8	0	1..145	24..168	9.00E-76	284
	NP_778539.1	Xylella fastidiosa Temecula1	hypothetical protein PD0304	80.00	87.59	145	28	1	1..145	24..167	8.00E-62	238
	NP_298298.1	Xylella fastidiosa 9a5c	hypothetical protein XF1008	79.31	86.90	145	29	1	1..145	24..167	7.00E-61	235
**GOE #87**	NP_780283.1	Xylella fastidiosa	hypothetical protein	85.27	90.70	129	19	0	2..130	5..133	1.00E-54	214

SSH39_912C_GOE_87 (130aa)		Temecula1	PD2113									
	NP_780287.1	Xylella fastidiosa Temecula1	hypothetical protein PD2117	86.40	91.20	125	17	0	6..130	1..125	1.00E-51	204
	NP_779440.1	Xylella fastidiosa Temecula1	hypothetical protein PD1239	80.00	86.15	130	26	0	1..130	1..130	2.00E-50	200
**GOE #88** SSH40_912C_GOE_88 (58aa)	ZP_00681103.1	Xylella fastidiosa Ann-1	conserved hypothetical protein	84.62	88.46	52	8	0	1..52	1..52	2.00E-11	71.2
	NP_779332.	Xylella fastidiosa Temecula1	hypothetical protein PD1126	79.07	83.72	43	9	0	3..45	7..49	5.00E-07	56.6
**GOE #89** SSH53_912C_GOE_89 (91aa)	No hit											
**GOE #90** SSH65_912C_GOE_90 (39aa)	No hit											
**GOE #91** SSH71_912C_GOE91 (98aa)	No hit											
**GOE #92** SSH72_912C_GOE_92 (155aa)	ZP_00682629.1	Xylella fastidiosa Ann-1	Phage integrase	98.61	99.31	144	2	0	1..144	77..220	1.00E-82	307
	ZP_00652696.1	Xylella fastidiosa Dixon	Phage integrase	98.61	99.31	144	2	0	1..144	77..220	2.00E-82	307
	ZP_00683736.1	Xylella fastidiosa Ann-1	Phage integrase	98.61	99.31	144	2	0	1..144	77..220	2.00E-82	306
**GOE #93** SSH73_912C_GOE_93 (138aa)	NP_779954.1	Xylella fastidiosa Temecula1	acyl-ACP-UDP-N-acetylglucosamine	33.33	52.78	108	72	0	7..114	48..155	1.00E-06	54.7
**GOE #94** SSH75_912C_GOE_94 (124aa)	ZP_00682689.1	Xylella fastidiosa Ann-1	VirB8	97.53	98.77	81	2	0	1..81	1..81	4.00E-35	149
	YP_980120.1	Verminephrobacter eiseniae EF01-2	VirB8 family protein	85.71	90.00	70	10	0	12..81	14..83	2.00E-24	114
**GOE #95** SSH01_Fb7_GOE_95 (100aa)	ZP_00652872.1	Xylella fastidiosa Dixon	ParB-like partition protein	84.88	88.37	86	13	0	1..86	211..296	6.00E-30	132
	YP_709193.1	IncP-1 plasmid pKJK5	KorB protein	51.09	63.04	92	37	2	1..86	211..300	6.00E-11	69.3
	NP_990880.1	Achromobacter denitrificans	KorB	45.65	60.87	92	43	2	1..86	220..310	1.00E-08	61.6
**GOE #96** SSH02_Fb7_GOE_96 (117aa)	ZP_00652871.1	Xylella fastidiosa Dixon	hypothetical protein XfasaDRAFT_0177	98.29	98.29	117	2	0	1..117	1..117	1.00E-52	207
	YP_112417.1	uncultured bacterium	KfrB protein	59.83	71.79	117	45	2	1..117	1..115	7.00E-28	125
	YP_195664.1	Azoarcus sp. EbN1	putative conjugation protein TraO	52.99	74.36	117	53	2	1..117	1..115	3.00E-27	123
**GOE #97** SSH05_Fb7_GOE_97 (96aa)	YP_001393241.1	Yersinia pseudotuberculosis IP 31758	conjugal transfer protein TrbA	55.88	76.47	68	28	1	1..66	50..117	7.00E-13	75.9
	YP_195561.1	Azoarcus sp. EbN1	TrbA protein of DNA transfer system	57.35	72.06	68	27	1	1..66	50..117	6.00E-12	72.8
	YP_974109.1	Acidovorax sp. JS42	helix-turn-helix domain protein	62.86	75.71	70	24	1	1..68	50..119	8.00E-11	68.9
**GOE #98** SSH06_Fb7_GOE_98 (44aa)	ZP_00652878.1	Xylella fastidiosa Dixon	type II secretion system protein E	95.00	95.00	40	2	0	1..40	2..41	1.00E-13	78.2
	NP_990919.1	Achromobacter	TrbB	50.00	75.00	40	20	0	1..40	1..40	4.00E-04	47.0

Query id	subject ids	organism	description	% identity	% positives	alignment length	Mismat-ches	gap opens	query start..end	subject start..end	E-value	Bit score
		denitrificans										
**GOE #99** SSH08_Fb7_GOE_99 (221aa)	ZP_00652867.1	Xylella fastidiosa Dixon	Relaxase/mobilization nuclease domain	94.18	95.24	189	11	0	16..204	454..642	2.00E-90	333
	YP_001393287.1	Yersinia pseudotuberculosis IP 31758	conjugal transfer protein TraI	51.82	68.18	110	53	0	23..132	462..571	5.00E-21	102
	NP_990929.1	Achromobacter denitrificans	TraI	53.26	76.09	92	43	0	26..117	425..516	2.00E-18	94.4
**GOE #100** SSH11_Fb7_GOE_100 (47aa)	ZP_00682615.1	Xylella fastidiosa Ann-1	Peptidase S24. S26A and S26B	100.00	100.00	24	0	0	24..47	229..252	1.00E-05	52.0
	ZP_00683692.1	Xylella fastidiosa Ann-1	Helix-turn-helix motif:Peptidase S24. S26A and S26B	95.83	100.00	24	1	0	24..47	383..406	8.00E-05	48.9
**GOE #101** SSH14_Fb7_GOE_101 (31aa)	ZP_00652876.1	Xylella fastidiosa Dixon	Plasmid encoded RepA protein	96.77	96.77	31	1	0	1..31	1..31	9.00E-08	58.9
**GOE #102** SSH15_Fb7_GOE_102 (116aa)	NP_779212.1	Xylella fastidiosa Temecula1	hypothetical protein PD1000	39.81	59.22	103	57	3	3..103	39..138	3.00E-09	63.9
	NP_298849.1	Xylella fastidiosa 9a5c	hypothetical protein XF1560	33.60	54.40	125	67	3	3..114	39..160	4.00E-09	63.2
	YP_001084613.1	Acinetobacter baumannii ATCC 17978	hypothetical protein A1S_1584	32.35	56.86	102	60	2	23..115	22..123	5.00E-06	53.1
**GOE #103** SSH17_Fb7_GOE_103 (200aa)	ZP_00650975.1	Xylella fastidiosa Dixon	similar to Uncharacterized phage-associated protein	58.14	66.86	172	66	1	1..172	1..166	8.00E-48	191
	NP_299081.1	Xylella fastidiosa 9a5c	hypothetical protein XF1793	71.43	80.22	91	26	0	1..91	1..91	3.00E-30	133
	YP_001197194.1	Flavobacterium johnsoniae UW101	Uncharacterized phage-associated protein	40.00	60.61	165	82	4	3..167	2..149	2.00E-28	127
**GOE #104** SSH19_Fb7_GOE_104 (61aa)	No hit											
**GOE #105** SSH23_Fb7_GOE_105 (106aa)	ZP_00652236.1	Xylella fastidiosa Dixon	hypothetical protein XfasaDRAFT_0897	34.62	52.88	104	65	1	1..101	8..111	1.00E-06	55.5
	ZP_00684057.1	Xylella fastidiosa Ann-1	conserved hypothetical protein	35.58	50.00	104	64	1	1..101	1..104	1.00E-05	51.6
	NP_299170.1	Xylella fastidiosa 9a5c	hypothetical protein XF1884	37.18	51.28	78	49	0	24..101	38..115	2.00E-04	47.8
**GOE #106** SSH31_Fb7_GOE_106 (113aa)	ZP_00682687.1	Xylella fastidiosa Ann-1	plasmid-related exported protein	91.84	94.90	98	8	0	1..98	1..98	6.00E-38	159
	YP_980122.1	Verminephrobacter eiseniae EF01-2	type IV secretion system family protein	66.33	77.55	98	33	0	1..98	1..98	5.00E-19	96.3
	YP_001257140.1	Brucella ovis ATCC 25840	P-type DNA transfer protein VirB5	44.58	71.08	83	46	0	16..98	9..91	5.00E-14	79.7
**GOE #107** SSH32_Fb7_GOE_107 (73aa)	AAL25255.1	Legionella pneumophila	TraJ	52.86	70.00	70	33	0	1..70	48..117	4.00E-12	73.6
	YP_001249533.1	Legionella pneumophila str. Corby	TraJ protein	47.95	65.75	73	34	1	1..73	48..116	6.00E-10	66.2

	YP_109861.1	Burkholderia pseudomallei K96243	putative plasmid conjugal transfer protein	67.61	80.28	71	23	0	1..71	52..122	7.00E-10	65.9
**GOE #108** SSH33_Fb7_GOE_108 (106aa)	ZP_00652867.1	Xylella fastidiosa Dixon	Relaxase/mobilization nuclease domain	98.81	98.81	84	1	0	6..89	46..129	5.00E-42	172
	YP_001393287.1	Yersinia pseudotuberculosis IP 31758	conjugal transfer protein TraI	60.42	79.17	96	32	2	4..98	43..133	1.00E-26	121
	YP_112410.1	uncultured bacterium	DNA relaxase	74.03	81.82	77	20	0	12..88	52..128	1.00E-26	121
**GOE #109** SSH37_Fb7_GOE_109 (76aa)	No hit											
**GOE #110** SSH39_Fb7_GOE_110 (139aa)	ZP_00682694.1	Xylella fastidiosa Ann-1	hypothetical protein XfasoDRAFT_2670	97.41	99.14	116	3	0	24..139	253..368	7.00E-46	185
	YP_980115.1	Verminephrobacter eiseniae EF01-2	hypothetical protein Veis_5018	91.15	93.81	113	10	0	27..139	316..428	1.00E-41	171
**GOE #111** SSH25_36f_GOE_111 (128aa)	ZP_00682696.1	Xylella fastidiosa Ann-1	hypothetical protein XfasoDRAFT_2672	96.75	97.56	123	4	0	1..123	1..123	2.00E-53	211
	YP_980148.1	Verminephrobacter eiseniae EF01-2	hypothetical protein Veis_5051	74.80	84.55	123	31	0	1..123	1..123	3.00E-32	140
**GOE #112** SSH32_36f_GOE_112 (66aa)	ZP_00684053.1	Xylella fastidiosa Ann-1	conserved hypothetical protein	98.48	100.00	66	1	0	1..66	66..131	2.00E-27	124
	AAW76134.1	Xanthomonas oryzae pv. oryzae KACC10331	conserved hypothetical protein	70.77	81.54	65	19	0	2..66	213..277	9.00E-20	98.6
	YP_988599.1	Bartonella bacilliformis KC583	hypothetical protein BARBAKC583_0275	69.23	84.62	65	20	0	2..66	67..131	1.00E-19	98.2
**GOE #113** SSH35_36f_GOE_113 (144aa)	ZP_00652972.1	Xylella fastidiosa Dixon	Phage/plasmid primase P4. C-terminal	64.56	74.68	79	23	2	71..144	765..843	4.00E-21	103
	ZP_00651851.1	Xylella fastidiosa Dixon	Phage/plasmid primase P4. C-terminal	65.82	73.42	79	22	2	71..144	765..843	6.00E-21	102
	ZP_00679796.1	Xylella fastidiosa Ann-1	Phage/plasmid primase P4. C-terminal	64.56	73.42	79	23	2	71..144	765..843	2.00E-20	100
**GOE #114** SSH36_36f_GOE_114 (64aa)	No hit											
**GOE #115** SSH38_36f_GOE_115 (44aa)	No hit											
**GOE #116** SSH11_187b_GOE_116 (126aa)	ZP_00652969.1	Xylella fastidiosa Dixon	Helix-turn-helix motif:Peptidase S24. S26A and S26B	98.41	99.21	126	2	0	1..126	99..224	2.00E-66	254
	NP_297986.1	Xylella fastidiosa 9a5c	phage-related repressor protein	61.90	77.78	126	47	1	1..126	97..221	6.00E-40	166
	ZP_00653051.1	Xylella fastidiosa Dixon	Peptidase S24. S26A and S26B	53.60	68.00	125	57	1	1..124	133..257	6.00E-30	132
**GOE #117** SSH18_187b_GOE_117 (95aa)	No hit											
**GOE #118** SSH22_187b_GOE_118 (112aa)	NP_779052.1	Xylella fastidiosa Temecula1	methyltransferase. type III restriction-modification system	100.00	100.00	82	0	0	1..82	260..341	5.00E-43	176
	NP_635609.1	Xanthomonas campestris pv.	methyltransferase	76.54	87.65	81	19	0	2..82	261..341	2.00E-32	140

		campestris str. ATCC 33913										
**GOE #119** SSH32_187b_GOE_119 (148aa)	No hit											
**Query id**	**subject ids**	**organism**	**description**	**% identity**	**% positives**	**alignment length**	**Mismat-ches**	**gap opens**	**query start..end**	**subject start..end**	**E-value**	**Bit score**
**GOE #120** SSH03_56a_GOE_120 (134aa)	ZP_00681945.1	Xylella fastidiosa Ann-1	Peptidase S24. S26A and S26B	38.10	59.05	105	57	4	1..101	153..253	3.00E-10	67.4
	NP_459874.1	Salmonella typhimurium LT2	putative transcriptional regulator	40.40	59.60	99	56	3	2..99	102..198	6.00E-09	62.8
	YP_207629.1	Neisseria gonorrhoeae FA 1090	putative lambda repressor protein cI. putative phage associated protein	32.31	51.54	130	75	3	2..131	112..228	6.00E-09	62.8
**GOE #121** SSH12_56a_GOE_121 (67aa)	No hit											
**GOE #122** SSH21_56a_GOE_122 (36aa)	ZP_00682695.1	Xylella fastidiosa Ann-1	hypothetical protein XfasoDRAFT_2671	100.00	100.00	36	0	0	1..36	299..334	4.00E-08	60.1
**GOE #123** SSH22_56a_GOE_123 (54aa)	No hit											
**GOE #124** SSH27_56a_GOE_124 (154aa)	ZP_00652969.1	Xylella fastidiosa Dixon	Helix-turn-helix motif:Peptidase S24. S26A and S26B	98.48	99.24	132	2	0	1..132	1..132	1.00E-65	251
	NP_297986.1	Xylella fastidiosa 9a5c	phage-related repressor protein	51.18	71.65	127	58	2	4..130	6..128	4.00E-27	123
	ZP_01787679.1	Haemophilus influenzae 22.4-21	hypothetical protein CGSHi22421_00742	30.23	58.14	129	88	2	1..127	1..129	5.00E-13	76.3
**GOE #125** SSH34_56a_GOE_125 (257aa)	ZP_00682693.1	Xylella fastidiosa Ann-1	TRAG protein	98.35	99.59	243	4	0	1..243	1..243	3.00E-130	465
	YP_980116.1	Verminephrobacter eiseniae EF01-2	TRAG family protein	87.65	95.06	243	30	0	1..243	1..243	2.00E-120	433
	NP_642936.1	Xanthomonas axonopodis pv. citri str. 306	VirD4 protein	51.74	63.18	201	83	5	45..242	49..238	2.00E-44	180
**GOE #126** SSH04_Cv21_GOE_126 (154aa)	NP_662757.1	Chlorobium tepidum TLS	type I restriction system specificity protein	42.13	48.73	197	53	4	3..138	124..320	1.00E-31	138
	YP_997187.1	Verminephrobacter eiseniae EF01-2	restriction modification system DNA specificity domain	42.95	58.33	156	61	2	11..138	143..298	7.00E-27	122
	NP_223444.1	Helicobacter pylori J99	putative TYPE I RESTRICTION ENZYME (SPECIFICITY SUBUNIT)	39.31	52.60	173	68	3	3..138	147..319	2.00E-25	117
**GOE #127** SSH05_Cv21_GOE_127 (98aa)	ZP_00684053.1	Xylella fastidiosa Ann-1	conserved hypothetical protein	98.98	100.00	98	1	0	1..98	34..131	3.00E-43	177
	YP_988599.1	Bartonella bacilliformis KC583	hypothetical protein BARBAKC583_0275	62.24	79.59	98	37	0	1..98	34..131	2.00E-28	127
	YP_770534.1	Rhizobium leguminosarum bv.	hypothetical protein pRL100255	61.22	79.59	98	38	0	1..98	34..131	3.00E-26	120

		viciae 3841										
**GOE #128** SSH07_Cv21_GOE_128 (83aa)	YP_980113.1	Verminephrobacter eiseniae EF01-2	hypothetical protein Veis_5018	78.57	83.93	56	12	0	1..56	1..56	4.00E-17	90.1
**GOE #129** SSH12_Cv21_GOE_129 (207aa)	ZP_00684136.1	Xylella fastidiosa Ann-1	Phage integrase	95.65	97.58	207	9	0	1..207	57..263	6.00E-102	371
	ZP_00651242.1	Xylella fastidiosa Dixon	Phage integrase	95.65	97.58	207	9	0	1..207	70..276	3.00E-101	369
	YP_284263.1	Dechloromonas aromatica RCB	Phage integrase	54.59	69.08	207	94	0	1..207	124..330	2.00E-52	207
**GOE #130** SSH20_Cv21_GOE_130 (206aa)	YP_554949.1	Burkholderia xenovorans LB400	hypothetical protein Bxe_B0347	30.46	46.36	151	94	6	9..157	18..159	5.00E-07	56.2
	ZP_00592014.1	Prosthecochloris aestuarii DSM 271	hypothetical protein PaesDRAFT_1019	28.19	42.95	149	84	6	10..155	10..138	4.00E-04	47.0
**GOE #131** SSH26_Cv21_GOE_131 (56aa)	NP_841356.1	Nitrosomonas europaea ATCC 19718	hypothetical protein NE1307	85.71	91.07	56	8	0	1..56	55..110	1.00E-20	101
	ZP_00682677.1	Xylella fastidiosa Ann-1	conserved hypothetical protein	85.71	87.50	56	8	0	1..56	23..78	1.00E-17	91.7
	YP_361540.1	Xanthomonas campestris pv. vesicatoria str. 85-10	hypothetical protein XCVc0024	80.36	85.71	56	11	0	1..56	53..108	8.00E-17	89.0
**GOE #132** SSH27_Cv21_GOE_132 (94aa)	NP_779332.1	Xylella fastidiosa Temecula1	hypothetical protein PD1126	90.11	94.51	91	9	0	4..94	56..146	1.00E-33	145
	ZP_00652684.1	Xylella fastidiosa Dixon	conserved hypothetical protein	90.11	95.60	91	9	0	4..94	26..116	5.00E-33	142
	ZP_00682619.1	Xylella fastidiosa Ann-1	conserved hypothetical protein	90.11	94.51	91	9	0	4..94	37..127	7.00E-33	142
**GOE #133** SSH33_Cv21_GOE_133 (149aa)	ZP_00682691.1	Xylella fastidiosa Ann-1	conjugation TrbI-like protein	96.30	98.52	135	5	0	15..149	298..432	1.00E-67	258
	YP_980118.1	Verminephrobacter eiseniae EF01-2	conjugation TrbI family protein	91.11	94.07	135	12	0	15..149	316..450	1.00E-64	248
	ABS71080.1	Salmonella enterica subsp. enterica serovar Dublin	TrwE	48.12	66.17	133	65	2	15..147	261..389	7.00E-29	129
**GOE #134** SSH36_Cv21_GOE_134 (61aa)	No hit											
**GOE #135** SSH45_Cv21_GOE_135 (92aa)	ZP_00682672.1	Xylella fastidiosa Ann-1	hypothetical protein XfasoDRAFT_2648	97.83	98.91	92	2	0	1..92	27..118	8.00E-30	132
	YP_980150.1	Verminephrobacter eiseniae EF01-2	hypothetical protein Veis_5053	63.04	76.09	92	34	0	1..92	26..117	5.00E-18	92.8
